# Irbesartan overcomes gemcitabine resistance in pancreatic cancer by suppressing stemness and iron metabolism via inhibition of the Hippo/YAP1/c-Jun axis

**DOI:** 10.1186/s13046-023-02671-8

**Published:** 2023-05-04

**Authors:** Tianxing Zhou, Yongjie Xie, Xupeng Hou, Weiwei Bai, Xueyang Li, Ziyun Liu, Quan Man, Jingyan Sun, Danqi Fu, Jingrui Yan, Zhaoyu Zhang, Yifei Wang, Hongwei Wang, Wenna Jiang, Song Gao, Tiansuo Zhao, Antao Chang, Xiuchao Wang, Hongxia Sun, Xiufeng Zhang, Shengyu Yang, Chongbiao Huang, Jihui Hao, Jing Liu

**Affiliations:** 1 Department of Pancreatic Cancer, Tianjin Medical University Cancer Institute and Hospital, National Clinical Research Center for Cancer; Key Laboratory of Cancer Prevention and Therapy, Tianjin’s Clinical Research Center for Cancer, Tianjin, 300060 PR China; 2 Department of Breast Oncoplastic Surgery and Department of Pancreatic Cancer, Tianjin Medical University Cancer Institute and Hospital, National Clinical Research Center for Cancer, Key Laboratory of Cancer Prevention and Therapy, Tianjin’s Clinical Research Center for Cancer, Key Laboratory of Breast Cancer Prevention and Therapy, Tianjin Medical University, Ministry of Education, Tianjin, 300060 PR China; 3 Department of Hepatopancreatobiliary Surgery, Tongliao City Hospital, Tongliao, 028000 Inner Mongolia China; 4 Department of Clinical Laboratory, Tianjin Medical University Cancer Institute and Hospital, National Clinical Research Center for Cancer, Key Laboratory of Cancer Prevention and Therapy, Tianjin’s Clinical Research Center for Cancer, Tianjin, China; 5 Beijing National Laboratory for Molecular Sciences (BNLMS), State Key Laboratory for Structural Chemistry of Unstable and Stable Species, Institute of Chemistry Chinese Academy of Sciences, Beijing, 100190 P. R. China; 6 College of Chemical Engineering, North China University of Science and Technology, Tangshan, 063210 China; 7 Department of Cellular and Molecular Physiology, Penn State College of Medicine, Hershey, PA USA; 8 Tianjin Medical University Cancer Institute and Hospital, National Clinical Research Center for Cancer, Key Laboratory of Cancer Prevention and Therapy, Tianjin, 300060 PR China

**Keywords:** Pancreatic cancer, Organoids, High-throughput drug screening, c-Jun, Irbesartan

## Abstract

**Background:**

Chemoresistance is the main reason for the poor prognosis of pancreatic ductal adenocarcinoma (PDAC). Thus, there is an urgent need to screen out new targets and compounds to reverse chemotherapeutic resistance.

**Methods:**

We established a bio-bank of human PDAC organoid models, covering a representative range of PDAC tumor subtypes. We screened a library of 1304 FDA-approved compounds to identify candidates efficiently overcoming chemotherapy resistance. The effects of the compounds were evaluated with a CellTiter-Glo-3D assay, organoid apoptosis assay and in vivo patient-derived xenograft (PDX), patient-derived organoid (PDO) and LSL-Kras^G12D/+^; LSL-Trp53^R172H/+^; Pdx1-Cre (KPC) genetically engineered mouse models. RNA-sequencing, genome editing, sphere formation assays, iron assays and luciferase assays were conducted to elucidate the mechanism.

**Results:**

High-throughput drug screening of chemotherapy-resistant PDOs identified irbesartan, an angiotensin ‖ type 1 (AT1) receptor antagonist, which could synergistically enhance the ability of chemotherapy to kill PDAC cells. In vitro and in vivo validation using PDO, PDX and KPC mouse models showed that irbesartan efficiently sensitized PDAC tumors to chemotherapy. Mechanistically, we found that irbesartan decreased c-Jun expression by inhibiting the Hippo/YAP1 pathway and further overcame chemotherapy resistance in PDAC. We also explored c-Jun, a potential target of irbesartan, which can transcriptionally upregulate the expression of key genes involved in stemness maintenance (SOX9/SOX2/OCT4) and iron metabolism (FTH1/FTL/TFRC). More importantly, we observed that PDAC patients with high levels of c-Jun expression demonstrated poor responses to the current standard chemotherapy regimen (gemcitabine plus nab-paclitaxel). Moreover, patients with PDAC had significant survival benefits from treatment with irbesartan plus a standard chemotherapy regimen in two-center retrospective clinical cohorts and patients with high c-Jun expression exhibited a better response to combination chemotherapy.

**Conclusions:**

Irbesartan could be used in combination with chemotherapy to improve the therapeutic efficacy in PDAC patients with high levels of c-Jun expression. Irbesartan effectively inhibited chemotherapy resistance by suppressing the Hippo/YAP1/c-Jun/stemness/iron metabolism axis. Based on our findings, we are designing an investigator-initiated phase II clinical trial on the efficacy and safety of irbesartan plus a standard gemcitabine/nab-paclitaxel regimen in the treatment of patients with advanced III/IV staged PDAC and are hopeful that we will observe patient benefits.

**Supplementary Information:**

The online version contains supplementary material available at 10.1186/s13046-023-02671-8.

## Background

Pancreatic ductal adenocarcinoma (PDAC) is a highly aggressive disease with a dismal prognosis [1]. FOLFIRINOX (folinic acid, 5-FU, irinotecan and oxaliplatin) and Gem-Abraxane (Gemcitabine/nab-paclitaxel) chemotherapy are still the mainstay chemotherapy regimens for PDAC. However, the response to both of these chemotherapy regimens is relatively poor, with rapid development of resistance in the majority of patients [2]. As a result, there is an urgent need to screen out new targets and compounds to reverse chemotherapeutic resistance [3]. Although numerous in vitro high throughput drug screens have been conducted using two-dimensional (2D) cancer cell line models, the success rate of the clinical translation of the screened drugs is extremely low [4–6].

Organoids are a kind of 3D self-renewing and self-organizing cultured structure. Patient-derived tumour organoids (PDOs) can undergo indefinitely expansion and accurately recapitulate major genomic and phenotypic features of patients’ primary tumors [7–9]. Pancreatic organoids were first established by Tuveson et al. in 2015 from mouse and human pancreatic adenocarcinoma tissues [10]. Later in the same year, Huang et al. demonstrated that pancreatic tumor organoids maintain the differentiation status, histoarchitecture and phenotypic heterogeneity of the primary tumor and retain patient-specific physiological changes, including hypoxia, oxygen consumption and epigenetic marks, which can be used to model PDAC and for drug screening to identify precision therapy strategies [11]. Recent publications have highlighted the utility of PDOs in drug screening [8, 9]. However, studies on drug screening using pancreatic tumor organoid models are still limited. The study conducted by Christian et al. included the largest set of compounds and was an automated drug-repurposing screen with 1,172 FDA-approved compounds using two PDAC PDOs [12].

In this study, we developed an integrated robotic high-throughput screening platform that combined the functions of automatic organoid culture, drug delivery, and cellular viability analysis. Using this fully automated screening strategy and three GEM-resistant PDAC PDOs, we screened 1304 FDA-approved compounds to identify candidates that could strongly reduce chemotherapy resistance. As a representative of the several compounds screened out, irbesartan was validated, and the potential mechanism of irbesartan in reducing chemotherapy resistance was explored using PDOs, KPC genetically engineered mouse models, PDO xenograft (PDOX) mouse models and patient-derived xenograft (PDX) mouse models, etc.

## Methods

More detailed information is provided in the Supplementary Methods.

### Patients and sample collection

A total of 95 patients who had received radical surgical R0 resection with a histological diagnosis of PDAC at the Tianjin Medical University Cancer Institute and Hospital, China between July 2011 and January 2015 were retrospectively enrolled in this study. Until the last follow-up date of October 23, 2019, the follow-up rate was 100%. Clinicopathological data of the 95 consecutive PDAC patients, including age, sex, histological grade, tumor size, TNM stage and regional lymph node status were obtained. None of the patients had received neoadjuvant chemotherapy or radiotherapy before tissue samples were collected. Systemic gemcitabine/nab-paclitaxel chemotherapy was given to all the patients after surgery.

Another retrospective cohort containing 58 patients who had received radical surgical R0 resection with a histological diagnosis of PDAC at the Tianjin Medical University Cancer Institute and Hospital, China, between April 2012 and December 2020 was enrolled in this study. Until the last follow-up date of December 30, 2021, the follow-up rate was 100%. Clinicopathological data of the 58 PDAC patients, including age, sex, histological grade, tumor size, TNM stage, regional lymph node status were obtained. None of the patients had received neoadjuvant chemotherapy or radiotherapy before tissue samples were collected. Systemic gemcitabine/nab-paclitaxel chemotherapy was given to all the patients after surgery.

Another retrospective cohort containing 104 patients who were pathologically diagnosed with advanced PDAC based on pancreatic needle biopsy at the Tianjin Medical University Cancer Institute and Hospital, China, between Jul 2015 and Jun 2020 was enrolled in this study. Until the last follow-up date of Jan 30, 2022, the follow-up rate was 100%. Clinicopathological data of the 104 PDAC patients, including age, sex, histological grade, tumor size, TNM stage, regional lymph node status were obtained. Systemic gemcitabine/nab-paclitaxel chemotherapy was given to all the patients after diagnosis. The chemotherapy response of PDAC patients were recorded and divided into three categories: partial remission (PR), stable disease (SD) and progressive disease (PD).

Another two retrospective cohorts were also enrolled to validate the effects of irbesartan. One cohort containing 60 patients with hypertension who were pathologically diagnosed with advanced PDAC based on pancreatic needle biopsy at the Tianjin Medical University Cancer Institute and Hospital, China, between Jul 2015 and Jun 2020 was collected in this study. Until the last follow-up date of Jan 30, 2022, the follow-up rate was 100%. Another cohort containing 60 patients with hypertension who were pathologically diagnosed with advanced PDAC based on pancreatic needle biopsy at Tongliao City Hospital, China, between Jan 2018 and Jan 2020 was enrolled in this study. Until the last follow-up date of Jun, 1, 2022, the follow-up rate was 100%. Clinicopathological data of the 60 PDAC patients, including irbesartan usage history, age, sex, histological grade, tumor size, TNM stage, regional lymph node status were obtained. Patients receiving other ARBs were excluded. Systemic gemcitabine/nab-paclitaxel chemotherapy was given to all the patients after diagnosis. GEM chemotherapy response of PDAC patients were recorded and divided into three categories: partial remission (PR), stable disease (SD) and progressive disease (PD).

Between March 2020 and April 2021, 31 consecutive fresh PDAC tissues were prospectively collected during surgery. The PDAC tissue specimens collected were divided into three parts, one part was grinded and digested into a single cell suspension for detection of CSCs (ESA^+^CD24^+^CD44^+^, ALDH^+^ and CD133^+^) by flow cytometry; the second part was fixed, embedded in paraffin and then prepared for IHC detection of c-Jun expression; the third part was used for measurement of iron content of tumor tissues.

The usage of these specimens and the patients’ information were approved by the Ethics Committee of Tianjin Medical University Cancer Institute and Hospital (Tianjin, China) and the Ethics Committee of Tongliao City Hospital (Tongliao, China). All patients provided written consent for the use of their specimens and disease information for future investigations according to the ethics committee and in accordance with recognized ethical guidelines of Helsinki (Approval No. AE-2021021 and 2,021,024).

### Establishment of primary PDAC cell lines

Fresh human PDAC tissue specimens from PDX-bearing mice were obtained during surgery and immediately washed with PBS three times. Blood clots, dead tissues and other connective tissues were removed. PDAC tissues were cut into small pieces (1mm^3^) and then these pieces were transferred into 15 ml conical centrifugal tubes (Corning) and resuspended in a mixture of 5 ml enzymes buffer containing 1 mg/ml collagenase(Sigma-Aldrich,C2799), 2.5 U/ml hyaluronidase (Sigma-Aldrich, H3506) and 0.1 mg/ml DNase (Sigma-Aldrich DN25) in 37 ℃ water bath for 4 ~ 6 h. The mixture was then filtrated in a 30 μm strainer (MACS Smart Strainer) to obtain single cell suspensions. The primary cancer cells were centrifuged, and the cell pellet was resuspended in fresh medium and seeded in 6-well plates. Detailed information on the PDX patients is listed in Supplementary Table 2. Low-passage (< 10 passages) primary cancer cells were used for later experiments.

### Cell culture and transfection

The human PDAC cell lines PANC-1, L3.7, MiaPaca-2 and SW1990 were obtained from the Type Culture Collection Committee of the Chinese Academy of Sciences (Shanghai, China). PDAC-vector, PDAC-c-Jun, PDAC-scramble, and PDAC-c-Jun-KD cell lines were established. PDAC cell lines were cultured in DMEM (Gibco, cat: A4192101) or RPMI 1640 basal medium (Gibco, cat: A1049101) supplemented with 10% fetal bovine serum(FBS) (Gibco, cat: 10,099,141) and 1% penicillin–streptomycin solution (Gibco, cat: 10,378,016) at 37 ℃ in a humidified atmosphere of 95% air and 5% CO_2_.

### The establishment and culture of patient-derived organoids (PDOs)

Organoids derived from human PDAC specimens were isolated and cultured as previously reported [8, 13]. In brief, fresh PDAC specimens were cut into small pieces (< 1 mm^3^) and washed three times with cold PBS supplemented with 10% penicillin and streptomycin. Then, the tissues were digested with digestion buffer with 1% foetal bovine serum, 10% penicillin/streptomycin, 1.5 mg/mL collagenase type II, 500 U/mL type collagenase IV, 0.1 mg/mL dispase type II and 10 mM Y-27,632 (Selleck, Shanghai, China)) for 45 min at 37 °C with vigorous vibration. After digestion, the tumour pellets were washed with cold PBS three times and finally collected through centrifugation at 200 × g for 5 min. Subsequently, tumour cells were embedded in Matrigel (BD) and plated in 96-well plates. After the polymerization of the Matrigel, these organoids were cultured in complete Advanced DMEM/F12 (Thermo Fisher Scientific) supplemented with Noggin (0.1 mg/ml, PeproTech), R-spondin (1 μg/ml, Nuvelo), epidermal growth factor (EGF, 50 ng/ml, PeproTech), Glutamax (Invitrogen), HEPES (Invitrogen), N2 (Invitrogen), B27 (Invitrogen), N-acetyl-L-cysteine (1 mM, Sigma), gastrin (10 nM, Sigma), nicotinamide (10 mM, Sigma), A83-01 (0.5 mM, Tocris Bioscience) and fibroblast growth factor 10( FGF10, 100 ng/ml, PreproTech). The organoid medium was changed approximately every 3 days, and organoids were passaged approximately every 7 days according to their growth conditions. Detailed information on the patients were listed in Supplementary Table 2. To determine the genomic background, single organoids were picked and expanded to generate clonal organoid lines which were characterized by western blotting and prepared for next generation sequencing (NGS).

### FDA-approved compound library

An FDA-approved compound library containing in total 1304 FDA-approved drugs (Cat:L4200, TOPSCIENCE, 100 μl in 10 mM DMSO stock) was acquired from the Perkin-Elmer G3 high-throughput drug screening platform at the Basic Medical Research Center of Tianjin Medical University. Detailed information on the drug library is listed in Supplementary Table 1.

### Fully automated screening of the FDA-approved compound library

Fully automated high-throughput screening in 3D organoids was conducted with a fully automated Perkin Elmer G3 integrated system. In brief, we chose gemcitabine-resistant organoids with high IC50 values for drug screening. Organoids cultured in Matrigel domes were collected and dissociated to single cell suspensions with a density of 5 × 10^5^ cells per ml. Then, the organoids suspensions were seeded in 384-well plates (Perkin Elmer, CellCarrier-384 plates, cat: 6,007,550) with one 10 μl dome per well by an automatic liquid handler integrated into the system. After dome seeding, the plates were incubated in an incubator at 37 ℃ for 30 min. Subsequently, complete organoid medium was added to 384-well plates at a volume of 20 μl per well by an automatic liquid handler integrated into the system. On the second day, gemcitabine, as basic chemotherapeutic regimen, was first added to the 384-well plates at a final concentration of 0.5 μM and then compounds of the FDA-approved drug library (L4200, TOPSCIENCE) as adjuvant chemotherapies were added to the 384-well plates at a final concentration of 1 μM. For each candidate, we established four groups: the DMSO group, GEM group, drug candidate group and combination regimen group. Gemcitabine plus erlotinib was used as a positive control and gemcitabine plus solvent control (DMSO) was used as a negative control. Culture of the 3D PDAC organoids treated with the chemotherapy regimens was continued for 72 h. The cellular viability of the 3D organoids was then evaluated by the CellTiter-Glo-3D assay, and the cell inhibitory rate of each regimen was calculated.

### CellTiter-Glo-3D assay

To evaluate the cell viability of organoids, a CellTiter-Glo 3D assay (Promega, cat: G9618) was performed. The culture medium of the organoids was discarded and 50 μL of prewarmed detection reagent was added according to the procedure. The cells were then incubated for 10 min at room temperature and luminescence was measured at 560 nm by a BioTek plate reader.

### Drug synergy analysis

We analyzed the synergistic effects of the combination therapy of gemcitabine and irbesartan using an organoid apoptosis assay. Synergy scores were calculated by SynergyFinder ver2 (https://synergyfinder.fimm.fi) [14, 15]. The final synergy scores were interpreted as follows: less than -10, the interaction between two drugs is likely to be antagonistic; between -10 and 10, the interaction between two drugs is likely to be additive; and greater than 10, the interaction between the two drugs is likely to be synergistic.

### Organoid apoptosis assay

The organoid apoptosis assay was performed as previously reported [16]. Briefly, the organoids cultured in 50 μl Matrigel were pretreated with gemcitabine, irbesartan and vehicle control and then organoid apoptosis was detected using a green-fluorescent capase3/7 probe reagent (Invitrogen, cat: R3711). Hoechst (Invitrogen, cat: 135,102) was added to the organoid culture system to visualize cells undergoing apoptosis. The apoptotic organoids were continuously monitored by an Operetta CLS high-content analysis system (Perkin Elmer) for 72 h and quantified using Harmony 4.5 software.

### Flow cytometry

PDAC cells were stained with anti-EpCAM, anti-CD133, anti-CD24, anti-CD44 and matched isotype control antibodies. ALDH activity was detected by an ALDEFLUOR kit (STEMCELL Technologies, cat: 01,700). The labile iron pool was evaluated by calcein-AM staining. Data were analyzed using FlowJo.V10.0.

### Q-PCR

Total RNA was extracted from PDAC cell lines using TRIzol reagent (Invitrogen). Then, the mRNA was used for first-strand cDNA synthesis with the Reverse Transcription PCR System (Bimake) according to the manufacturer’s instructions. RT-PCR was performed to measure the mRNA levels of the target genes. Each RT-PCR experiment was repeated independently at least three times. Actin was used as a loading control. Detailed information of the PCR primer sequences used is listed in Supplementary Table 12.

### Western blotting

Whole-cell extracts were prepared by lysing cells with SDS protein lysis buffer supplemented with proteinase inhibitor cocktail (Bimake, B14001). Proteins in the lysates were separated by SDS-PAGE, and then, the target proteins were detected by immunoblotting with primary antibodies. β-Tubulin was used as a loading control. Goat anti-rabbit or anti-mouse secondary antibodies were used at a 1:5000 dilution (Abmart). Detailed information on the antibodies used for western blotting is listed in Supplementary Table 11.

### Sphere formation assay

PDAC cells (5000 cells/ml) were seeded in ultralow-adhesion 6-well plates (Corning, cat: CLS4520) in serum-free medium. After two weeks, tumor spheres with a diameter of > 75 μm were counted.

### Animals

Female NOD/SCID and BALB/c-nude mice were purchased from SPF Biotechnology Company, Beijing, China and maintained in specific pathogen-free conditions. An LSL-Kras^G12D^; LSL-Trp53^R172H^; Pdx1-Cre genetically engineered mouse models was established in house.

### KPC mouse preclinical cohorts

The pancreatic tumor volume in KPC mice was monitored twice a week by MRI scanning. When the pancreatic tumor in KPC mice had developed and grown to 20 ~ 60mm3, the mice were randomized into four groups: (A) vehicle (normal saline), (B) gemcitabine/nab-paclitaxel (gemcitabine purchased from MCE, HY-17026,100 mg/kg intraperitoneally once a week; nab-paclitaxel purchased from HengRui, diluted in normal saline, 300 mg/kg intravenously once a week), (C) irbesartan (purchased from MCE, HY-B0202; predissolved in olive oil; 20 mg/kg twice a week by oral gavage) and (D) gemcitabine/nab-paclitaxel + irbesartan. The mice were then separated into 2 sets. In set 1(8 mice per group), the drug was administrated from the time that he tumors reached 20 ~ 60mm^3^ until death. In set 2 (6 mice per group), the mice were treated as described for set 1 but were sacrificed after 8 weeks of treatment to compare tissues. The volume of the pancreatic tumors was monitored twice a week by MRI scanning. In set 2, pancreatic tumor tissues were harvested and weighed. The tumor tissues were immediately fixed in buffered formalin and embedded in paraffin and another part were stored at -80 ℃ for protein extraction and western blot analysis. Tissue slides (5 μm) were prepared, and haematoxylin & eosin (H&E) staining was performed for histopathological analysis according to instructions. IHC staining of Ki67 (Abcam, ab16667) staining was performed to evaluate the cell proliferation status in the tumor tissues. Tissues proteins from the vehicle group and rosiglitazone group were prepared for western blotting to analyze the expression of c-Jun.

### In vivo limiting dilution tumorsphere formation assay

NOD/SCID mice were randomized into different groups and the indicated cancer cells at various dilutions were subcutaneously transplanted into the contralateral flanks of the mice. The observers and recorders in the study were blinded to the grouping. The stem cell frequency was calculated on website http://bioinf.wehi.edu.au/software/elda/.

### Subcutaneous mouse model

Mice were randomized into different groups and 3D organoids, PDXs and other cancer cell lines were subcutaneously injected. The observers and recorders in the study were blinded to the grouping. Tumor growth was monitored once a week using a caliper and tumor volumes were calculated by the following formula: Volume = 1/2 L1 × (L2)^2^, where L1 is the length of the long axis and L2 is the length of the short axis.

### Orthotopic mouse model

Mice were randomized into different groups and 3D organoids and luciferase-expressing cancer cell lines were orthotopically injected. The observers and recorders in the study were blinded to the grouping Tumor growth was monitored by MRI scanning and bioluminescence imaging (BLI) and mouse survival was also recorded.

### RNA-sequencing

Total RNA from samples was extracted using TRIzol (Invitrogen) according to the manufacturer’s instructions. The quality and integrity of the total RNA were evaluated using the A260/280 ratio and agarose gel electrophoresis. The mRNA samples were prepared for transcriptomic sequencing according to the following process. First, mRNA was enriched and purified from total RNA using oligo (dT) beads. The purified mRNA was cleaved into short fragments using fragmentation buffer and the fragments were used as templates to synthesize first-strand and second-strand cDNA. After end repair and the poly (A) tailing, the cDNA fragments were ligated with Illumina sequencing adapters. The ligation products were enriched by PCR amplification to construct the cDNA library template. Finally, the library was sequenced using an Illumina HiSeqTM 2500 instrument by Gene Denovo Biotechnology Co. (Guangzhou, China). The expression level of each gene was calculated as the fragments per kilobase of transcript per million mapped reads (FPKM) value. Differentially expressed genes (DEGs) between groups were identified by the edgeR package (http://www.rproject.org/) with thresholds of “false discovery rate (FDR) < 0.05 and absolute log2-fold change ≥ 1”.

### Statistical analysis

Statistical analyses were performed with IBM SPSS Statistics software version 21.0. Each experiment was conducted in triplicate. The data values are presented as the means ± SDs, unless otherwise stated. The variance between different groups was statistically compared. Power analysis was conducted on the results. Student’s t test was used to compare the mean values. The median survival time was analyzed using Kaplan–Meier curves and the log-rank test was used to analyze differences in the survival time among the different groups. Spearman rank correlation analysis was performed to evaluate the correlation between different parameters. The differential expression of targeted genes in paired tumour and non-tumour tissues was analyzed by the Wilcoxon signed-rank test. Two-way repeated measures ANOVA (tumour volume × time) and post-hoc analyses were carried out for analysis of mouse tumor growth. The risk factors associated with the prognoses of these patients were evaluated with a Cox proportional hazards regression model. *P < 0.05; **P < 0.01; ***P < 0.001; ****P < 0.001 and n.s., non-significant.

## Results

### Fully automated high-throughput drug screening identifies potential compounds overcoming GEM resistance in PDAC using 3D organoid models

**Fig. 1:**
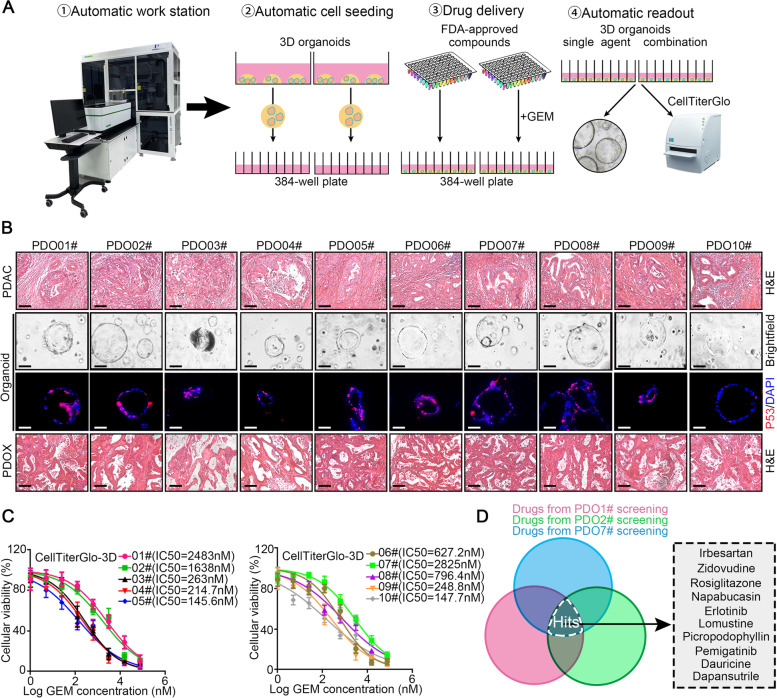
Fully automatic high-throughput drug screening identifies potential compounds overcoming gemcitabine resistance of PDAC using 3D organoid models. **A** Schematic illustration of the fully automatic high-throughput screening strategy. **B** The bright field images and immunofluorescence staining of p53 for 10 human PDAC organoids lines, corresponding with H&E staining images of the parental human PDAC tissues and patient-derived organoid xenografts (PDOX) tissues in mice. Scale bar: 100 μm. **C** The IC50 value of GEM in each PDO line. **D** The identified hits screened out from PDO01#/02#/07# lines were visualized as Venn plot

To identify candidates that could overcome intrinsic GEM resistance in PDAC, we developed a fully automated screening strategy using an integrated robotic screening platform (Fig. 1A). A drug library containing 1304 FDA-approved drugs was used in the screen (Table S1). To improve the screening efficacy, 3D patient-derived organoids (PDOs) with high GEM resistance were obtained from our established biobank (Table S2).

All PDOs were derived from surgically resected specimens from PDAC patients, and systemic GEM-based chemotherapy was given to all patients after surgery. PDAC patients whose organoids exhibited a better response to GEM exhibited longer RFS times during the retrospective clinical follow-up, which validated the strong correlation between drug responses in the cancer organoids and the corresponding patients. The phenotypes of the organoids in our established biobank strongly correlated with the morphology of the primary PDAC tumors in the corresponding patients (Fig. 1B). NGS analyses indicated that the mutation frequency of KRAS/P53/CDKN2A (> 90%) in the PDAC organoids was comparable to that in their matched parental PDAC tumors (data not shown). Furthermore, the structures of the organoids ranged from hollow cysts consisting of a single-layered epithelium with uniform nuclei, to dense multi-layered structures (Fig. 1B). We also observed different expression levels of TP53 in these organoids (Fig. 1B), which indicated the genomic diversity of the organoids. The IC50 of GEM was determined by CellTiter-Glo-3D assays using the ten PDO models in our biobank (Fig. 1C and Table S2). Finally, the GEM-resistant PDOs (PDO01#/02#/07#) with the highest IC50s were recruited for drug screening.

The GEM-resistant PDOs (PDO01#/02#/07#) were treated with GEM alone, the candidate compound X alone, or the candidate compound combined with GEM [GEM + X]. The corresponding inhibitory rates of organoid viability in each treatment group determined by the CellTiter-Glo-3D assay were defined as X_**i**_, GEM_**i**_ and [GEM + X]_**i**_. To confirm the cytotoxic effects and synergistic effects of the combination therapy, we defined the screening criteria for hit compounds: [GEM + X]_**i**_ > 60% and [GEM + X]_**i**_ > GEM_**i**_ + X_**i**_. Based on the screening criteria, 10 hit compounds were identified from the intersection of the screening results using the GEM-resistant PDOs (Table S3 and Fig. 1D). Among the 10 hits, 6 compounds have already been reported to overcome GEM resistance (napabucasin, erlotinib, picropodophyIlin, lomustine, rosiglitazone and zidovudine) [17–23]; notably, erlotinib exhibited success in recent clinical trials of PDAC, which further validated our approaches.

### Irbesartan efficiently overcomes the GEM resistance of PDAC in PDO, PDX and GEM-resistant BxPC-3 cell models in vitro and in vivo

We concentrated our efforts on the remaining candidate drugs (pemigatinib, dapansutrile, dauricine and irbesartan) and the effects of these drugs were validated in vitro/in vivo. Among the remaining four candidate drugs, irbesartan (an AT1 receptor antagonist) presented the most significant cytotoxic effects and synergistic effects with GEM (Fig. S1; additional in vivo validation data about pemigatinib/dapansutrile/dauricine are not shown). In addition, the side effects of irbesartan were minimal [24]. As a result, irbesartan was chosen for further investigation.

**Fig. 2:**
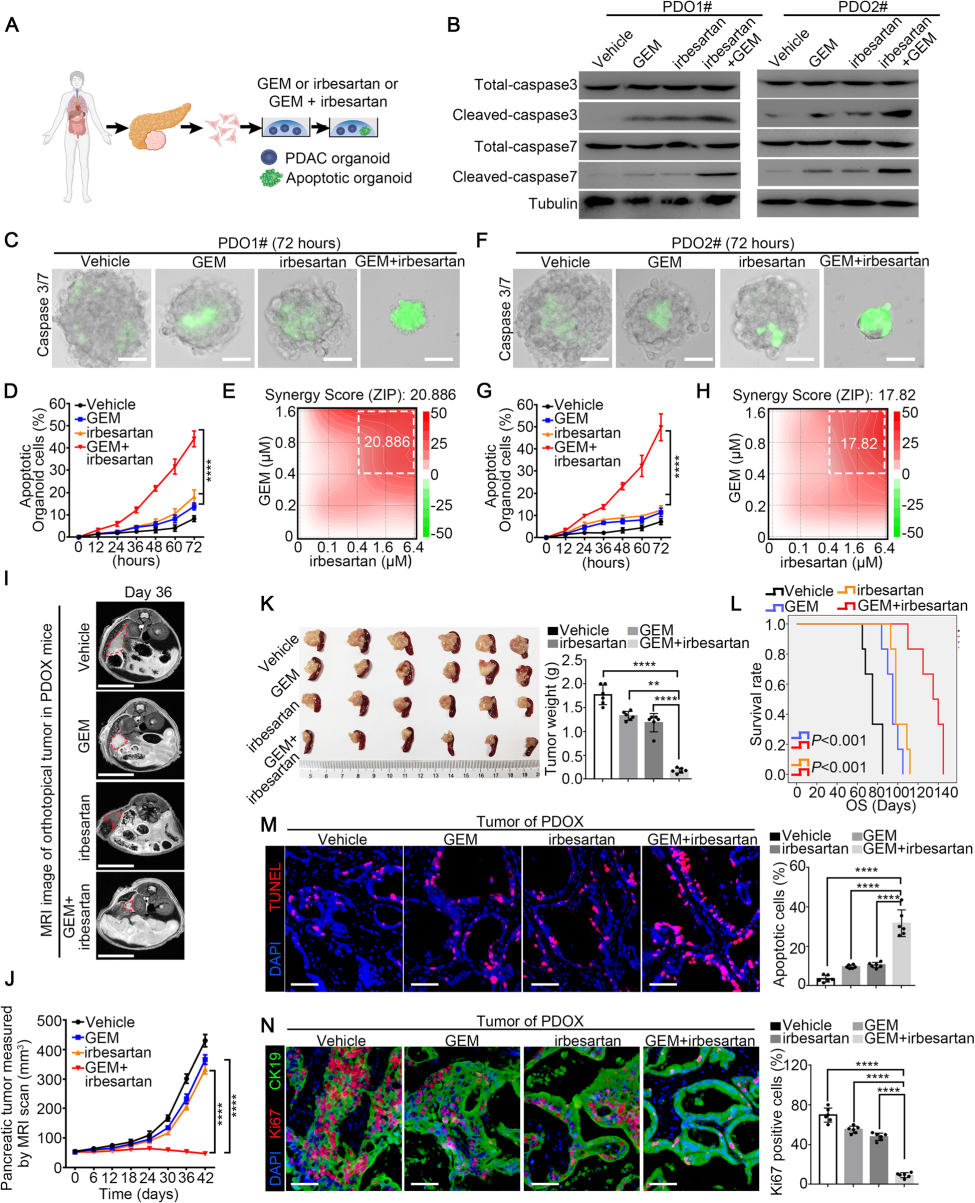
Irbesartan efficiently overcomes GEM resistance of PDAC in PDO, PDX and GEM-resistant BxPC-3 models in vitro and in vivo. **A** The schematic illustration for human PDAC organoid apoptosis evaluation. **B** The apoptosis of organoids treated with GEM (0.8 μM) and irbesartan (1 μM) for 72 h was determined by western blot for cleaved-caspase 3/7. Tubulin was used as loading control. **C-H** Organoids were treated with vehicle, GEM (0.8 μM), irbesartan (1 μM) and GEM (0.8 μM) plus irbesartan (1 μM), followed by continually monitoring PDAC organoid apoptosis by real-time caspase3/7 probe imaging for 72 h. Representative apoptotic images of organoids in 72 h (PDO1#, C; PDO2#, F) and real-time apoptotic imaging analysis (PDO1#, D; PDO2#, G) were shown. For analyzing the synergy score of irbesartan and GEM, organoids were treated with drugs in a constant concentration (irbesartan 0–6.4 μM, GEM 0–1.6 μM) for 72 h using Synergy finder (PDO1#,E; PDO2#,H). **I-J** Tumor volumes of PDOX were monitored by MRI scan. Representative MRI images per group at day 36 were shown (**I**) and tumor volumes were calculated by MRI scan (*n* = 6 per group, **J**). **K** Representative pancreatic tumor images per group at the experimental ending were shown (left) and tumor weight was determined (right). **L** Kaplan–Meier survival curves with log-rank test were used to analyze the effects after drug treatment in another cohort (*n* = 6). **M**–**N** The apoptotic and proliferative level of PDOX tumor in mice were evaluated by TUNEL staining and Ki67 staining. All experiments were repeated three times independently. Paired Student’s t-test were used for in vitro experiments. Un-paired Student’s t-test were used for in vivo experiments

We first tested the effects of irbesartan on inducing organoid apoptosis. PDO01# and PDO02# were treated with vehicle, irbesartan, GEM and the combined regimen of irbesartan and GEM (Fig. 2A). The combined regimen significantly induced the expression of the apoptotic markers of cleaved-caspase3 and cleaved–caspase7 (Fig. 2B). As shown in Fig. 2C, F and Fig. S2A-B, the size of the organoids in GEM group decreased about 40% compared with vehicle group, however, the size of the organoids in the combined regimen group decreased approximately 90%. The intensity of the fluorescence labeled caspase3/7 was much stronger in the combined regimen group than in the monotherapy group (Fig. S2C-D). As shown in Fig. 2C-D and F-G, the combined regimen significantly increased the ability of GEM to induce organoid apoptosis compared with that of monotherapy in a time-dependent manner. Although our data showed that irbesartan combined with GEM had stronger cytotoxic effects than either drug alone, the interaction between irbesartan and GEM was unknown. Therefore, we used SynergyFinder2.0 to further analyze the combinatorial effects of the two drugs. The comprehensive synergy scores and synergy maps of the drug combination are shown in Fig. 2E, H. The comprehensive synergy scores of irbesartan with GEM (20.866 in PDO01# and 17.82 in PDO02#) indicated that irbesartan and GEM exhibited synergistic cytotoxic effects in the tested concentration range. Moreover, compared to the monotherapies, the combined regimen significantly reduced the immunofluorescence intensity of Ki67 staining, which indicated synergistic inhibition of PDAC cell proliferation in PDO models (Fig. S2E-F).

To further evaluate the efficacy of GEM plus irbesartan in vivo, the GEM-resistant PDO01# was used to establish orthotopic patient-derived organoid xenograft (PDOX) mouse models. When the tumor volumes reached approximately 50mm^3^, the mice were randomly assigned to 4 groups: the vehicle, GEM monotherapy, irbesartan monotherapy and combination regimen groups. Animals treated with the combination regimen had a significantly decreased tumor burden (Fig. 2I-J). Compared with the monotherapies, GEM plus irbesartan significantly reduced the weight of pancreatic tumor (Fig. 2K) but did not change the body weight of mice (Fig. S2G). More importantly, significant survival benefits were observed in the combination regimen group (median survival time: 135 days) compared with the monotherapy groups (median survival time: 95 days in GEM group; 98 days in irbesartan group) (Fig. 2L). The results of the TUNEL assay and Ki67 immunofluorescence staining in mouse pancreatic tumor tissues indicated that the combined regimen notably increased the proportion of apoptotic cancer cells and reduced the proportion of proliferative cancer cells when compared with those in the monotherapy groups (Fig. 2M-N). Subcutaneous PDO xenograft mouse models were established using PDO02# to PDO10# (Fig. S3). Moreover, ten subcutaneous PDX mouse models were used to test the therapeutic effect of combination regimen in comparison with the vehicle control and monotherapies (Table S2). As expected, the combination regimen significantly decreased the tumor volumes compared with those in the monotherapy groups (Fig. S4). Consistent results were obtained in the in vitro and in vivo experiments using the GEM-resistant BxPC-3 cell line (Fig. S5).

### Irbesartan synergistically sensitizes PDAC to GEM/nab-paclitaxel (GEM/AB) therapy in the genetically engineered KPC mouse model

**Fig. 3:**
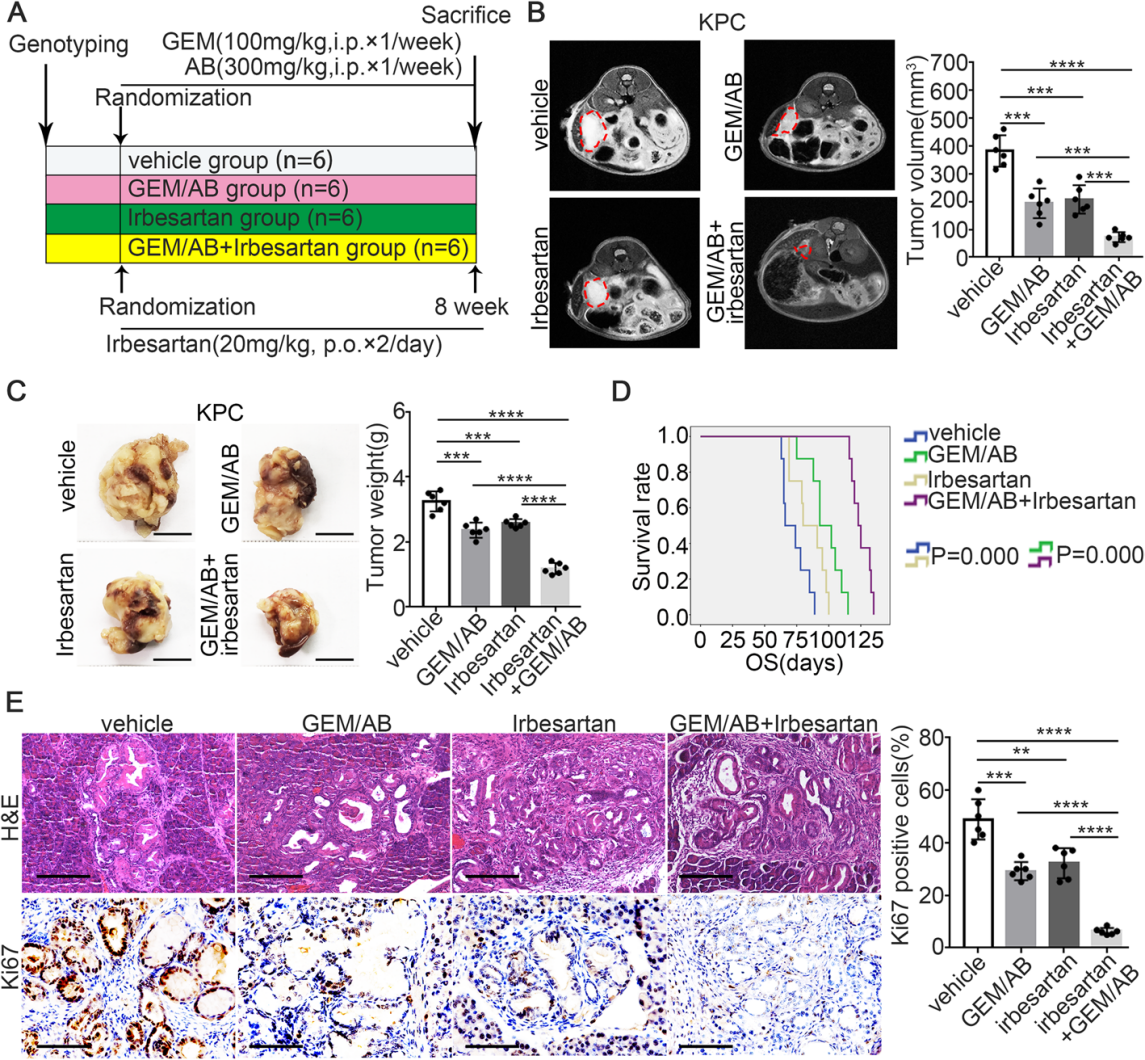
Irbesartan synergistically sensitizes PDAC to GEM/nab-paclitaxel (GEM/AB) therapy in KPC genetic mouse model. **A** Schematic illustration for the experimental design. **B** Representative MRI images of KPC mice treated with vehicle (*n* = 6), GEM/AB (*n* = 6), irbesartan (*n* = 6) and GEM/AB plus irbesartan (*n* = 6) at day 35 after drug treatment (left). Pancreatic tumor volumes were shown (right). **C** Representative macroscopic images of pancreatic tumors in KPC mice (left). Statistical analysis for tumor weight of KPC mice (right). **D** Kaplan–Meier survival curves with log-rank test for KPC mice in each group (*n* = 8). **E** Representative images of H&E slides and Ki67 IHC staining from tumors of four groups (left). Scale bars: 200 µm. Statistical analysis for percentage of Ki-67 positive cells in different groups (right). The mouse experiments were repeated three times independently, and non-paired Student’s t-test was used for statistical analysis

Considering that GEM/nab-paclitaxel (GEM/AB) is the current standard regimen for advanced PDAC treatment recommended by international NCCN guidelines [25], to further improve the clinical translational significance of our findings, we next evaluated the therapeutic effects of irbesartan plus GEM/AB in the genetically engineered KPC mouse model. When the tumor volumes of KPC mice reached 20-60mm^3^ as determined by MRI scanning, the mice were randomized into four groups as shown in Fig. 3A. The in vivo dose of GEM/AB was determined as previously reported [26]. MRI scanning showed that the tumor burden was significantly decreased in the GEM/AB plus irbesartan group compared with the GEM/AB group on Day 45 (Fig. 3B). Consistent with this finding, the combination therapy reduced the weight of pancreatic tumors in comparison to that in the GEM/AB group (Fig. 3C). More importantly, mice in the GEM/AB plus irbesartan group had better survival benefits (Fig. 3D). In addition, IHC stainning of Ki67 indicated that the combination therapy led to a significant decrease in the proportion of proliferative cancer cells compared with that in the GEM/AB group (Fig. 3E). Taken together, the above results suggested that irbesartan synergistically sensitizes PDAC to GEM/AB therapy in the genetically engineered KPC mouse model.

### Irbesartan overcomes chemotherapy resistance in PDAC in a c-Jun dependent manner

**Fig. 4:**
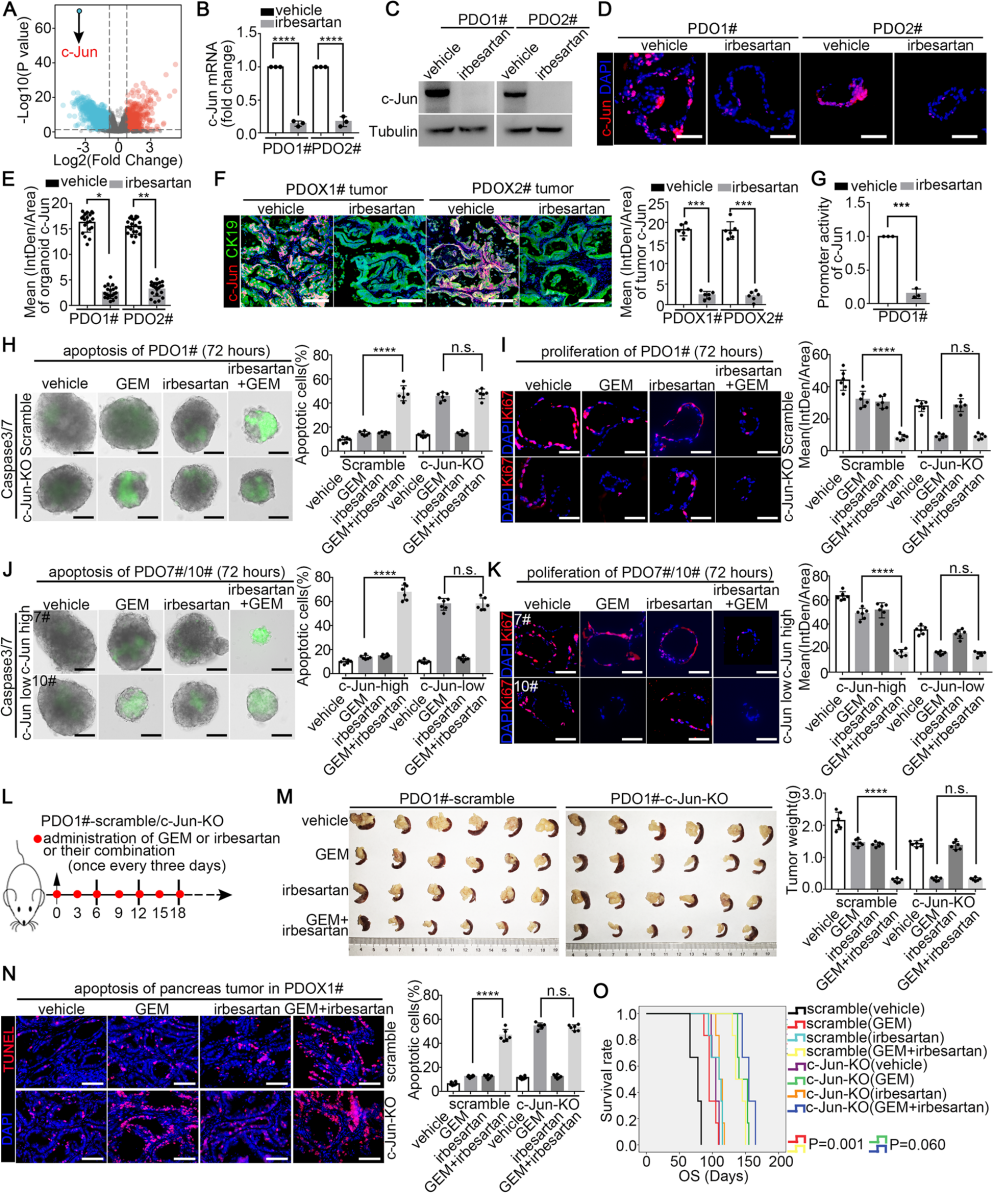
Irbesartan overcomes gemcitabine resistance of PDAC in a c-Jun dependent manner. **A** RNA-sequencing was performed on PDO1# line treated with vehicle and irbesartan and the DEGs were represented as volcano plot. **B**-**E** The expression of c-Jun in organoids treated with vehicle and irbesartan was evaluated by Q-PCR (**B**), western blot (**C**) and immunofluorescence staining (**D**-**E**). **F** The expression of c-Jun in PDOX of mice in vivo was determined by multiplex IHC staining. **G** The promoter activity of c-Jun in PDO1# after treating with irbesartan (1 μM, 72 h) was detected by dual luciferase assay. **H**-**I** PDO1#-scramble/c-Jun-KO lines were treated with vehicle, GEM, irbesartan and GEM plus irbesartan for 72 h (irbesartan 1 μM, GEM 0.8 μM), followed by monitoring organoid apoptosis by real-time caspase3/7 probe imaging (**H**) and evaluating organoid proliferation by Ki-67 immunofluorescence staining (**I**). **J**-**K** PDO7#/PDO10# were treated with vehicle, GEM, irbesartan and GEM plus irbesartan for 72 h (irbesartan 1 μM, GEM 0.8 μM), followed by monitoring organoid apoptosis by real-time caspase3/7 probe imaging (**J**) and evaluating organoid proliferation by Ki-67 immunofluorescence staining (**K**). **L** The experimental setup for in vivo PDOX assay. **M** Representative pancreatic tumor images per group at the experimental ending were shown (left) and tumor weight was determined (right). **N** The apoptotic level of PDOX tumor in mice were evaluated by TUNEL staining. **O** Kaplan–Meier survival curves with log-rank test were used to analyze the effects after drug treatment in another cohort (*n* = 6). All experiments were repeated three times independently. Paired Student’s t-test were used for in vitro experiments. Un-paired Student’s t-test were used for in vivo experiments

To identify the mechanism by which irbesartan increases GEM sensitivity, we used RNA-sequencing to determine the effects of irbesartan on the transcriptome in PDO01#. As shown in Fig. 4A and Table S4, c-Jun, a canonical member of the AP-1 family, ranked first among the downregulated differentially expressed genes (DEGs) after irbesartan treatment (Log_2_ (fold change) = -3.559; DEGs filtering criteria: |Log_2_ (fold change)|> 1, FDR < 0.25 and *P* < 0.05). We found that c-Jun was highly expressed in PDAC (Fig. S6A-F). High c-Jun expression was positively correlated with clinicalpathological factors associated with aggressiveness and was an independent prognostic factor of OS and RFS (Tables S5 and S6 and Fig. S6G-H). The Q-PCR, western blot analysis and immunofluorescence staining results showed that irbesartan significantly reduced the mRNA and protein expression of c-Jun in the PDO, PDX and GEM-resistant tumor models in vitro and in vivo (Fig. 4B-F and Figs. S7 and S8). Considering that irbesartan acts as a specific AT1R antagonist, we also evaluated the expression pattern of AT1R in pancreatic cancer. Single cell-RNA sequencing analysis of PDAC specimens from our laboratory showed that the AT1R positive cells were mainly distributed in the tumor cell and cancer-associated fibroblast (CAF) population (Fig. S9A-C) [27]. In addition, the western blot results for collections of established PDAC cell lines, in vitro organoids, in vivo PDO subcutaneous tumors and primary human CAFs showed that AT1R was expressed in both tumor cells and CAFs (Fig. S9D-G), consistent with previous reports [28]. Further luciferase assays indicated that irbesartan notably repressed the transcriptionally activity of the c-Jun promoter in PDAC (Fig. 4G). Thus, we speculated that irbesartan could be a promising c-Jun inhibitor.

To determine whether irbesartan reduces GEM resistance by suppressing c-Jun expression, we established a c-Jun knockout PDO1# cell line by CRISPR/dCas9 gene editing. The efficiency of c-Jun knockout was validated, as shown in Fig. S9H. As shown in Fig. 4H-I, c-Jun KO markedly increased the GEM sensitivity of PDO01#. However, irbesartan failed to further enhance GEM sensitivity in the c-Jun-KO group, suggesting that irbesartan increased GEM sensitivity through c-Jun. Consistent with this idea, the c-Jun high PDO07#, exhibited a better response to irbesartan plus GEM combination therapy; while the c-Jun low PDO10# line responded poorly to the combination therapy (Fig. S9I, Fig. 4J-K and Fig. S3F,I).

An orthotopic PDOX mouse model was established using PDO01#-c-Jun-KO and PDO01#-scramble tumors for in vivo evaluation (Fig. 4L). In the PDO01#-scramble xenograft tumor model, the combined regimen significantly decreased the tumor burden compared with that in mice treated with GEM alone. However, in the PDO01# c-Jun-KO xenograft tumor model, no differences in tumor weight, tumor apoptosis or survival benefits were observed between the GEM monotherapy group and the combined regimen group (Fig. 4M-O). Taken together, these findings indicated that irbesartan enhanced the effect of GEM in a c-Jun dependent manner.

### Irbesartan decreases c-Jun expression by repressing activation of the Hippo/YAP1 signaling pathway in PDAC

**Fig. 5:**
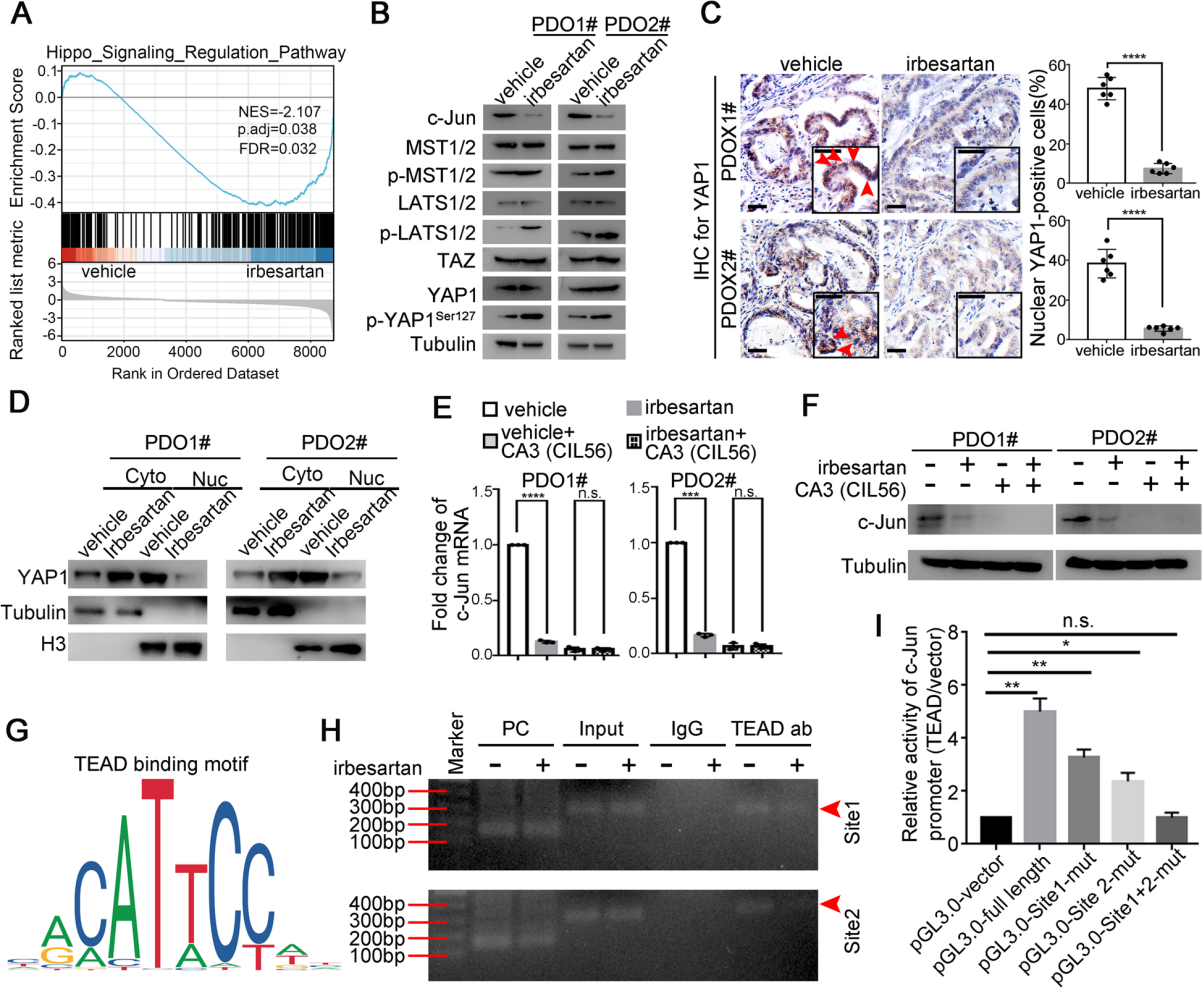
Irbesartan decreases c-Jun expression via repressing activation of Hippo/YAP1 signal pathway in PDAC. **A** Enrichment score profile of the Hippo/YAP1 signal pathway in PDO1# treated with irbesartan (1 μM) (DMSO was used as vehicle control) in GSEA analysis. **B** The effects of irbesartan on the protein expression of Hippo/YAP1 signal pathway (MST1, p-MST1, LATS1, p-LATS1, YAP1, p-YAP1 (Ser127), TAZ) in indicated PDO lines were evaluated by western blot. **C** The expression of cytosol and nuclear YAP1 in subcutaneous PDOX1# and PDOX2# tumors treated with vehicle and irbesartan were evaluated by IHC. Nuclear YAP1-positive tumor cells each group were analyzed. Red arrows indicated nuclear YAP1.Scale bar, 50 μm. **D** The cytosol and nuclear fractions for YAP1 in PDO1# and PDO2# lines were determined by western blot. **E**–**F** The effects of irbesartan on c-Jun mRNA (**E**) and protein (**F**) expression in PDO1# and PDO2# lines could be fully abrogated by Hippo pathway inhibitor, CA3 (CIL56). Actin was used as internal control in Q-PCR and tubulin was used as loading control for western blot. **G** The binding motif of the TEAD from JASPAR. **H** CHIP analysis of PDO1# lines pretreated with or without irbesartan (1 μM) for 24 h. Chromatin was immunoprecipitated with anti-TEAD1 antibodies and then subjected to PCR analysis. **I** Dual luciferase assay was performed to evaluate the effects of YAP1/TEAD on transcriptional activity of c-Jun promoter in PDO1# lines. All experiments were repeated three times independently. Paired Student’s t-test were used for in vitro experiments. Un-paired Student’s t-test were used for in vivo experiments

To further investigate the molecular mechanism by which irbesartan decreases c-Jun expression in PDAC, GSEA analysis using RNA-sequencing data of PDO01# treated with vehicle and irbesartan was conducted. As shown in Fig. 5A, the Hippo/YAP1 signaling pathway was among the pathways with the highest enrichment scores. Since the activity of YAP1 is inhbited by its phosphorylation [29], we first evaluated the effect of irbesartan treatment on YAP phosphorylation. Although irbesartan did not affect the expression of total YAP1, it exerted a profound effect on the level of phosphorylated-YAP (Ser127) (Fig. 5B). We next assessed the phosphorylation status of LATS1 and MST1, a negative regulator of YAP1 activation. Similar to its molecular action on YAP1, irbesartan significantly induced the phosphorylation of LATS1 and MST1 without changing the levels of total LATS1 and MST1 (Fig. 5B). Upon phosphorylation by LATS1 and other kinases, YAP1 is retained in the cytoplasm and becomes transcriptionally inactive form. We therefore evaluated YAP1 levels in both the cytosol and nucleus of PDAC cells. As shown in Fig. 5C-D, upon irbesartan treatment, nuclear YAP1 level decreased, but the cytosolic YAP1 level increased in the indicated cell lines, compared with the vehicle control cells, demonstrating that irbesartan inhibits nuclear translocation of YAP1 in PDAC cells. Furthermore, we found that blockade of the Hippo/YAP1 signaling pathway via the inhibitor, CA3 (CIL56), completely abrogated the effects of irbesartan on c-Jun expression (Fig. 5E-F), indicating that irbesartan decreases c-Jun expression by repressing the activation of the Hippo/YAP1/TAZ signaling pathway in PDAC. Indeed, YAP interacts with the DNA-binding transcription factor, TEAD, to regulate target gene promoter activity, as previously described [30]. Two potential TEAD binding sites in the promoter of YAP1 were identified by the online transcription factor prediction software JASPAR. Our Ch-IP results also revealed that TEAD strongly bound in the two predicted sites of the promoter of c-Jun, and that irbesartan treatment notably decreased the occupancy of TEAD on the promoter of c-Jun in PDAC cells (Fig. 5G-H). Further luciferase assays with constructs harboring mutations in each TEAD binding site confirmed that each site was needed for full promoter activity, while mutation of both sites fully abrogated luciferase activity, demonstrating that TEAD is involved in c-Jun promoter activity (Fig. 5I).

### Tumoral c-Jun induces chemotherapy resistance in PDAC

**Fig. 6:**
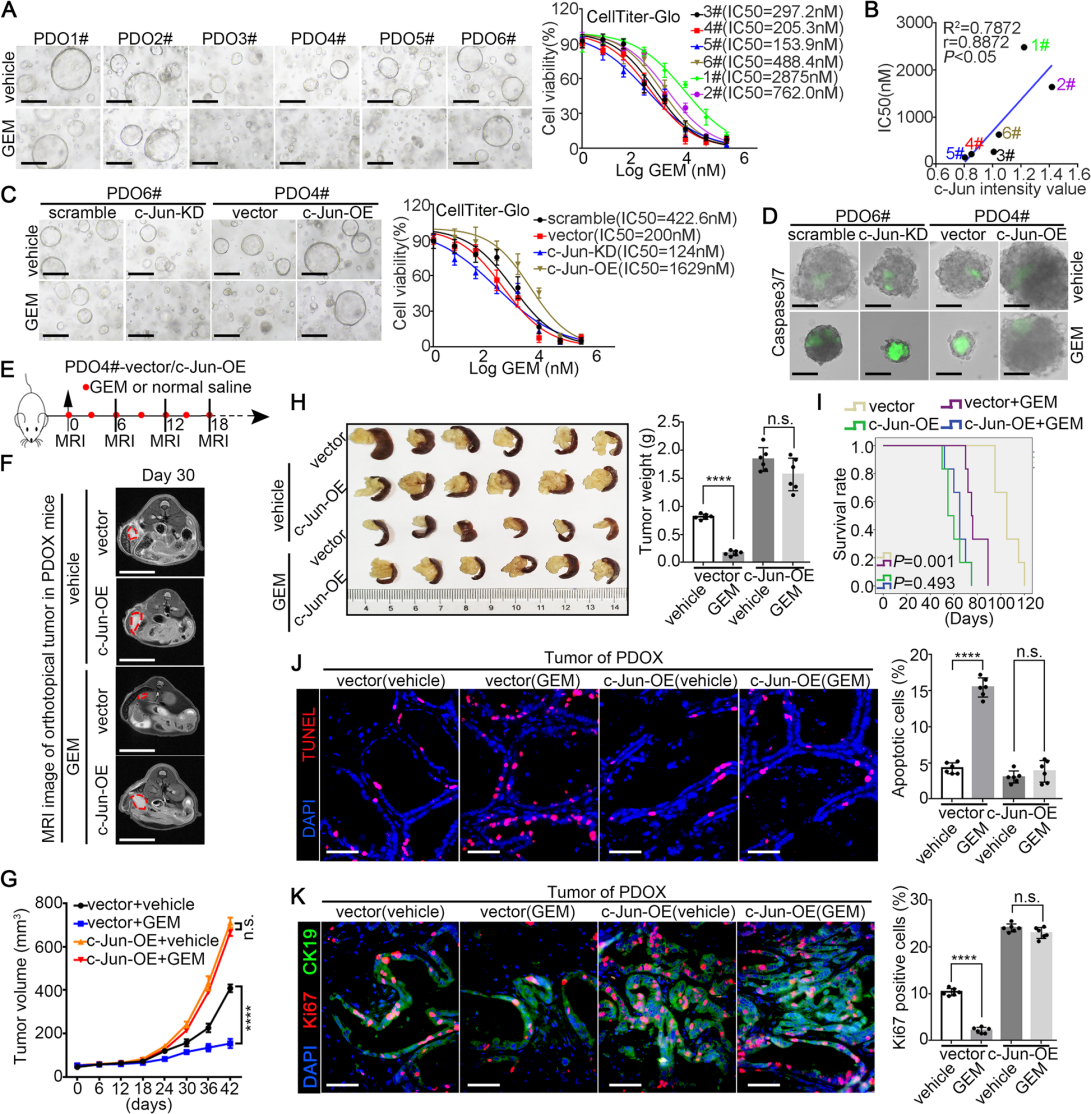
Tumoral c-Jun induces chemotherapy resistance of gemcitabine in PDAC. **A**The representative bright field images of PDOs treated with GEM (400 nM, 72 h) were shown (left).The IC50 values of gemcitabine in each PDO were analysed by CellTiterGlo-3D assay (right). **B** Spearman correlation analysis between c-Jun expression and the IC50 of PDOs. **C** PDO6#-scramble/c-Jun-KD and PDO4#-vector/c-Jun-OE were treated with gemcitabine. The representative bright field images of organoids treated with GEM (400 nM, 72 h) (C, left). The IC50 value of gemcitabine in PDO6#-scramble/c-Jun-KD and PDO4#-vector/c-Jun-OE was analysed by CellTiterGlo-3D assay (C, right). **D** The representative images of apoptotic organoids by caspase3/7 probe labelled were shown and the percentage of apoptotic organoid cells were analysed in Fig. S10F. **E** The experimental design for in vivo PDOX assay. **F**-**G** Tumor volumes of PDOX were monitored by MRI scan. Representative MRI images per group at day 30 were shown (**F**) and tumor volumes were calculated by MRI scan (*n* = 6 per group, **G**). **H** Representative pancreatic tumor images per group at the experimental ending were shown (left) and tumor weight was determined (right). **I** Kaplan–Meier survival curves with log-rank test were used to analyze the effects after drug treatment in another cohort (*n* = 6). **J**-**K** The apoptotic and proliferative level of PDOX tumor in mice were evaluated by TUNEL staining (**J**) and Ki67 staining (**K**). All experiments were repeated three times independently. Paired Student’s t-test were used for in vitro experiments. Un-paired Student’s t-test were used for in vivo experiments

Considering that irbesartan can reduce chemoresistance through suppressing c-Jun expression, we explored whether c-Jun promotes intrinsic resistance to GEM in PDAC. First, we analyzed the correlation between GEM sensitivity and endogenous c-Jun expression in PDX cell lines and organoid models. As shown in Fig. S10A-D and Fig. 6A-B, the endogenous c-Jun levels in 2D PDX cell lines and 3D organoids were significantly positively correlated with their IC-50 values of GEM. Furthermore, c-Jun deletion increased GEM sensitivity in resistant PDOs. Conversely, ectopic c-Jun overexpression in GEM-sensitive PDOs increased resistance to GEM treatment (Fig. S10E-F and Fig. 6C-D). We also generated an orthotopic PDOX model using the PDO-c-Jun-OE line (Fig. 6E). Our data showed an increased tumor burden in the PDO-c-Jun-OE group compared to the PDO-vector group and that overexpression of c-Jun prevented the gemcitabine-induced decrease in the tumor burden (Fig. 6F-H). Besides, worse survival benefits to animals were observed in the c-Jun overexpression group than in the vector group when GEM was administrated (Fig. 6I). The results of the TUNEL assay and Ki67 immunofluorescence staining in mouse pancreatic tumor indicated that a higher proliferative capacity and lower apoptotic rate in PDO-c-Jun-OE tumors than in PDO-vector tumors, and that c-Jun overexpression increased the resistance of PDAC tumors to the cytotoxic effects of GEM (Fig. 6J-K). In addition, we found that the expression of c-Jun was highly elevated in GEM-resistant cells compared with non-GEM-resistant cancer cells (Fig. S11A). Knock down of c-Jun in GEM-resistant BxPC-3 cell lines significantly sensitized these cells to GEM treatment (Fig. S11B). These results were further confirmed in PDX-derived PDAC cell lines (Fig. S11C-I).

### Tumoral c-Jun functionally enhances stemness and iron metabolism pathways activity in PDAC

**Fig. 7:**
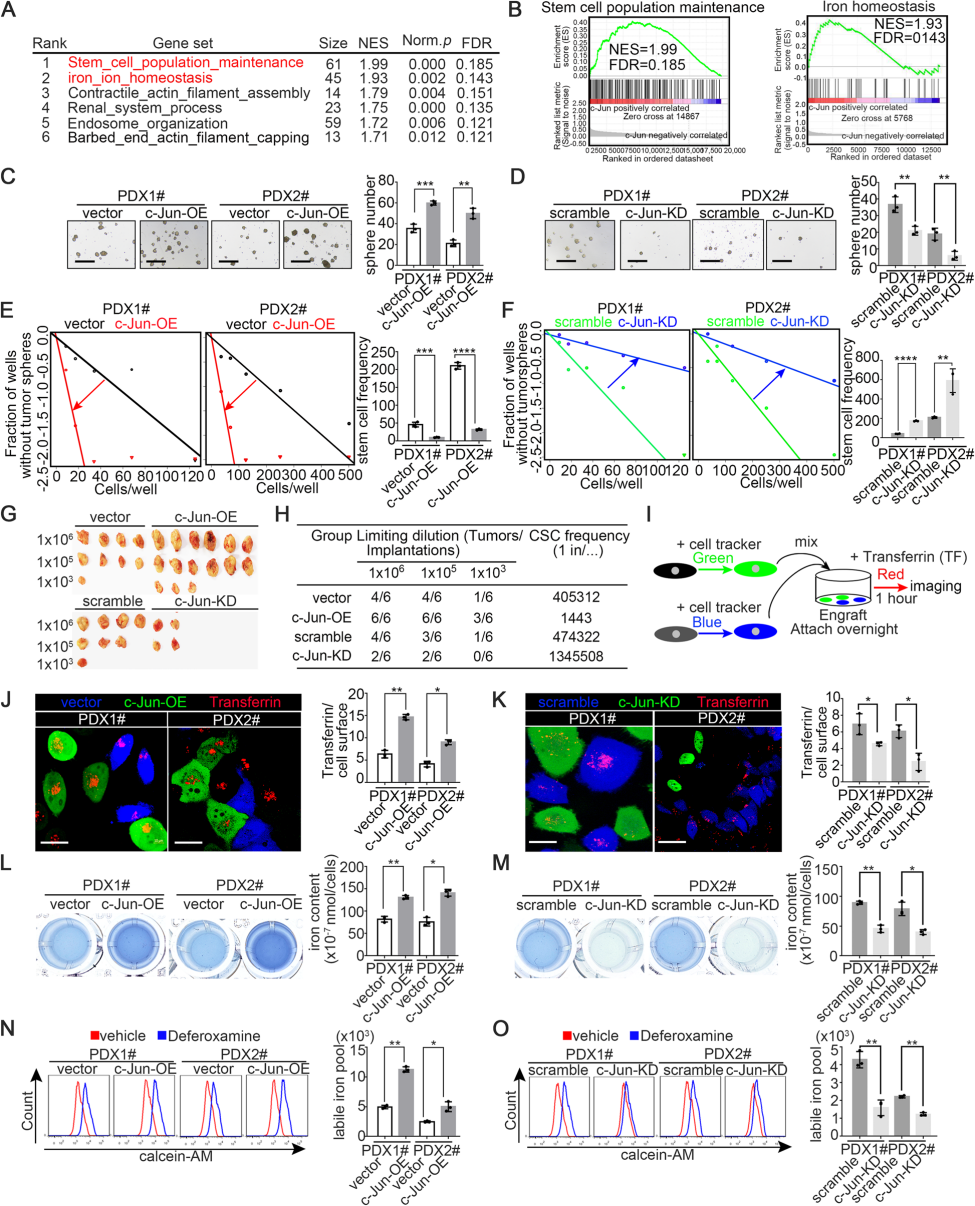
Tumoral c-Jun functionally increases stemness and iron metabolism pathways in PDAC. **A** Top enriched pathways in PDO4#-vector/c-Jun-OE and primary cell line PDX1#-vector/c-Jun-OE based on Gene Set Enrichment Analysis on RNA-sequencing data. NES, normalized enrichment score, Norm. *p*, normalized *P* value. **B** Enrichment score profile of the stemness (left) and iron homeostasis pathway (right) in GSEA. **C**-**D** Sphere formation assays were performed in indicated cell lines. **E**–**F** In vitro limited dilution assays were performed in indicated cell lines. **G**-**H** In vivo limited dilution assays for PDX1# cell lines were performed. Representative tumor images (**G**) and tumor incidence/CSC probabilities (**H**) were shown. **I** Schematic illustration of the experimental procedure for the in vitro transferrin uptake in cancer cells. **J**-**K** One representative lane from the 3D reconstructions of representative fields showing transferrin (red) in vector or scramble cells (blue) and c-Jun-OE or c-Jun-KD cells (green) and quantification of fluorescent-transferrin as measured on the surface of the 3D reconstruction. **L**-**M** Iron ion content was determined in indicated cell lines (**N**–**O**) Relative libel iron pool (LIP) in indicated cell lines were determined by FCM. MFI of calcein in cells with or without defetoxamine (DFO) treatment were analyzed and ΔMFI were calculated as libel iron pool (LIP). All experiments were repeated three times independently. Paired Student’s t-test were used for in vitro experiments

To define the mechanism by which c-Jun induces GEM resistance in PDAC, the effects of c-Jun overexpression on the trancriptome were evaluated in PDO4# and PDX1# were evaluated. GSEA (gene set enrichment analysis) analysis showed that the stemness maintenance and iron homeostasis pathways were among the pathways with the highest enrichment scores (Fig. 7A-B and Tables S7, S8 and S9) [31, 32].

To determine whether c-Jun might play a role in pancreatic cancer stem cells (CSCs) maintenance, we collected fresh PDAC tissues from a prospective cohort of 31 patients. As shown in Fig. S12A-E, the IHC score of c-Jun was positively correlated with the proportion of cells positive for CSC markers, including the CD133^+^, ALDH^+^ and ESA^+^CD44^+^CD24^+^ subsets. In addition, multiplexed staining and TissueFAXS analysis in archived tissues from a retrospective cohort of 95 PDAC patients showed that the counts of CD133^+^ cells and ALDH1^+^ cells per high-power field were notably increased in the high-c-Jun group compared with in the low-c-Jun group (Fig. S12F-G). Ectopic expression of c-Jun in primary PDX lines increased the proportion of CSCs (Fig. S13A, C, E) and the sphere formation capacity (Fig. 7C, E); conversely, c-Jun knockdown decreased the CSC population (Fig. S13B, D, F) and sphere formation capacity (Fig. 7D-F) compared with those in PDX-scramble lines. To further examine the role of c-Jun in pancreatic cancer cell stemness, a limited dilution tumorigenesis assays was performed. Ectopic expression of c-Jun significantly increased, but knockdown of c-Jun decreased, the proportion of CSCs in the limited dilution tumorigenesis assay (Fig. 7G-H). Thus, our results suggested that c-Jun strongly promotes cancer cell stemness in PDAC.

Next, we explored whether c-Jun regulates iron metabolism. The iron content was measured using fresh tumor tissues from the prospective cohort of 31 PDAC patients. As shown in Fig. S12H, the IHC score of c-Jun was positively correlated with the iron content in PDAC tissues. We subsequently evaluated preferential iron uptake ability in PDAC samples with different expression levels of c-Jun, and we conducted an in vitro coculture experiment in which fluorescently labeled cancer cells were subjected to transferrin loading and imaging (Fig. 7I). PDAC-vector/scramble cells were pre-labelled with Cell Tracker Blue and PDAC-c-Jun-OE/c-Jun-KD cell lines were pre-labelled with Cell Tracker Green, respectively. Subsequently, PDAC-vector cells were mixed with PDAC-c-Jun cells and PDAC-scramble cell were mixed with PDAC-c-Jun-KD cells, respectively. Next, the mixed cancer cells were counted and seeded in 6-well plate overnight. The following day, the culture medium was replaced with conditioned medium containing red-conjugate transferrin (TF). Images of red-conjugate transferrin uptake for tumor cells in each mixed system were captured on Laser Scanning Confocal Microscopy (Zeiss) with a Z stack mode and then 3D reconstruction of TF co-localization with the tumour cell were performed. The capacity of transferrin uptake were quantified by measuring the ratio of area of cellular TF and area of cellular surface. As shown in Fig. 7J-K, the single representative plane from the 3D reconstructed image of the co-localization of transferrin with the tumor cell surface demonstrated that the amount of bound transferrin was significantly increased in the primary cell line PDX-c-Jun-OE and significantly decreased in PDX-c-Jun-KD cells compared with their corresponding controls. Furthermore, we measured the cellular iron content in the primary cell lines PDX-c-Jun-OE/KD line and found that c-Jun overexpression significantly increased the cellular iron content, while c-Jun knockdown notably reduced the cellular iron content (Fig. 7L-M). The cellular labile iron pool (LIP) is used for iron mobilization and reflects the level of iron metabolism. c-Jun overexpression significantly elevated the LIP, while c-Jun knockdown notably reduced the LIP (Fig. 7N-O). To further validate our findings in vitro, we also measured the tissue iron content and LIP of subcutaneous tumors, as shown in Fig. 5G, and found that the iron content and LIP were significantly increased in the primary cell line PDX1#-c-Jun-OE, but decreased in PDX1#-c-Jun-KD cells (Fig. S13G-H). Taken together, these findings indicated that tumoral c-Jun could notably enhance tumoral iron metabolism in PDAC.

### Tumoral c-Jun binds to the promoters of stemness and iron metabolism-related genes to upregulate their transcription

**Fig. 8:**
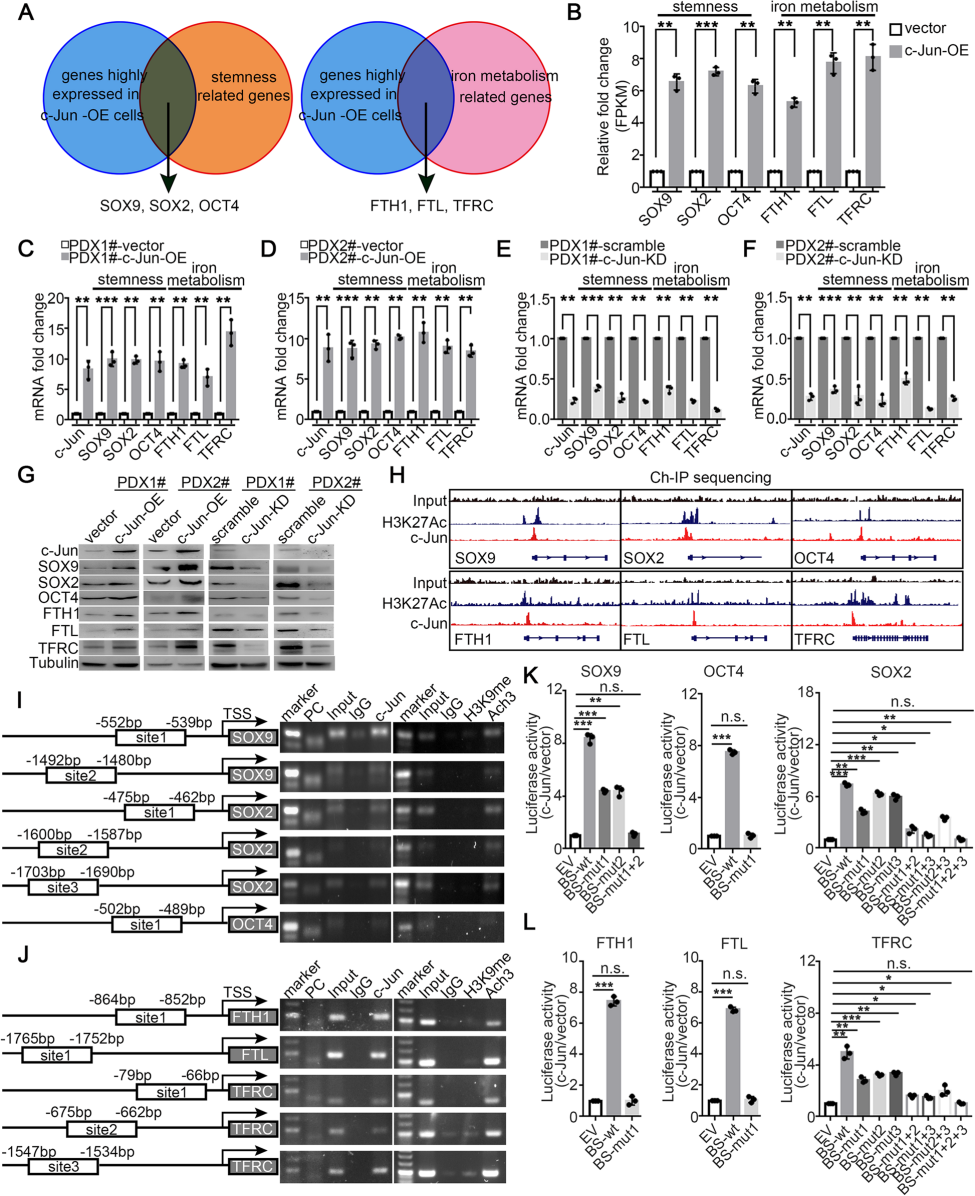
Tumoral c-Jun binds to the promoter of stemness and iron metabolism related genes to up-regulate their transcription. **A** Venn diagram of stemness genes and iron metabolism genes from RNA-sequencing data of PDX1#-vector/c-Jun-OE. **B** RNA-seq expression level for a panel of stemness and iron metabolism gene in the PDX1#-vector/c-Jun-OE cell lines. **C**-**F** Q-PCR for c-Jun/SOX9/SOX2/NANOG/OCT4/FTH1/FTL/ TFRC were performed in indicated cell lines. **G** Western blot for c-Jun/SOX9/SOX2/NANOG/OCT4/FTH1/FTL/ TFRC were performed in indicated cell lines. **H** ChIP-seq on PDX1#-c-Jun cell lines were performed. Representative ChIP-seq input, H3K27ac, and c-Jun peaks for stemness genes (SOX9/SOX2/OCT4) and iron metabolism genes (FTH1/FTL/TFRC). **I**-**J** Ch-IP assay was performed to validate binding of c-Jun on promoter of indicated genes in PDX1# cell lines. **K**-**L** Dual luciferase assay was performed to determine the promoter activity in PDX1#-vector/c-Jun-OE cell lines. Renilla luciferase activity was used as internal control. All experiments were repeated three times independently. Paired Student’s t-test were used for in vitro experiments

To further elucidate the molecular mechanism by which c-Jun regulates stemness and iron metabolism in PDAC, stemness/iron metabolism-related genes from the RNA-sequencing data of the primary cell line PDX1#-c-Jun-OE were screened and SOX9/SOX2/OCT4 and FTH1/FTL/TFRC were determined to be the most significantly upregulated genes in the dataset (Fig. 8A-B). First, Q-PCR and western blotting showed that the expression of SOX9, SOX2, OCT4, FTH1, FTL and TFRC was all positively regulated by c-Jun (Fig. 8C-G). Importantly, tumoral c-Jun expression was positively correlated with SOX9, SOX2, OCT4, FTH1, FTL and TFRC expression in consecutive PDAC tumour tissues (Fig. S14). The c-Jun Ch-IP-sequencing data showed that c-Jun predominantly bound to the promoter regions between the H3K27ac double peaks in stemness and iron metabolism genes, suggesting that c-Jun could transcriptionally upregulate the expression of these genes (Fig. 8H). Based on the CHIP-sequencing data, we surveyed the promoter regions of these genes for potential c-Jun binding sites. Computational analysis showed several high-confidence binding sites corresponding in the promoter regions of stemness genes (SOX9/SOX2/OCT4) and iron metabolism genes (FTH1/FTL*/*TFRC) in the JASPAR database (Fig. 8I-J, left). Ch-IP assays were performed in the PDX-c-Jun-OE cell line and revealed that c-Jun obviously bound to the promoters of these genes (Fig. 8I-J, right). Further luciferase assays using the PDX-c-Jun-OE/vector cell lines were performed to determine whether binding of c-Jun to the promoters of these genes induces their transcription. Our data demonstrated that c-Jun overexpression significantly increased the transcription of stemness-related genes (SOX9/SOX2/OCT4) and iron metabolism-related genes (FTH1/FTL/TFRC). Mutation of binding site 1 in the promoters of OCT4, FTH1 and FTL completely abolished the trans-activation of the promoters of the three genes by c-Jun; mutation of binding site 1 or site 2 in the promoter of SOX9 only partially suppressed promoter activity, while dual mutation of binding site1 and site 2 could fully abrogate the promoter activity; and mutation of binding site 1, site 2, site 3, site 1 + 2, site 1 + 3 or site 2 + 3 in the promoters of SOX2 and TFRC only partially decreased promoter activity, while simultaneous mutation of binding sites1, 2 and 3 could completely abrogate the promoter activity induced by c-Jun (Fig. 8K-L).

### Tumoral c-Jun promotes chemotherapy resistance through dual pathways including stemness and iron metabolism in PDAC

**Fig. 9:**
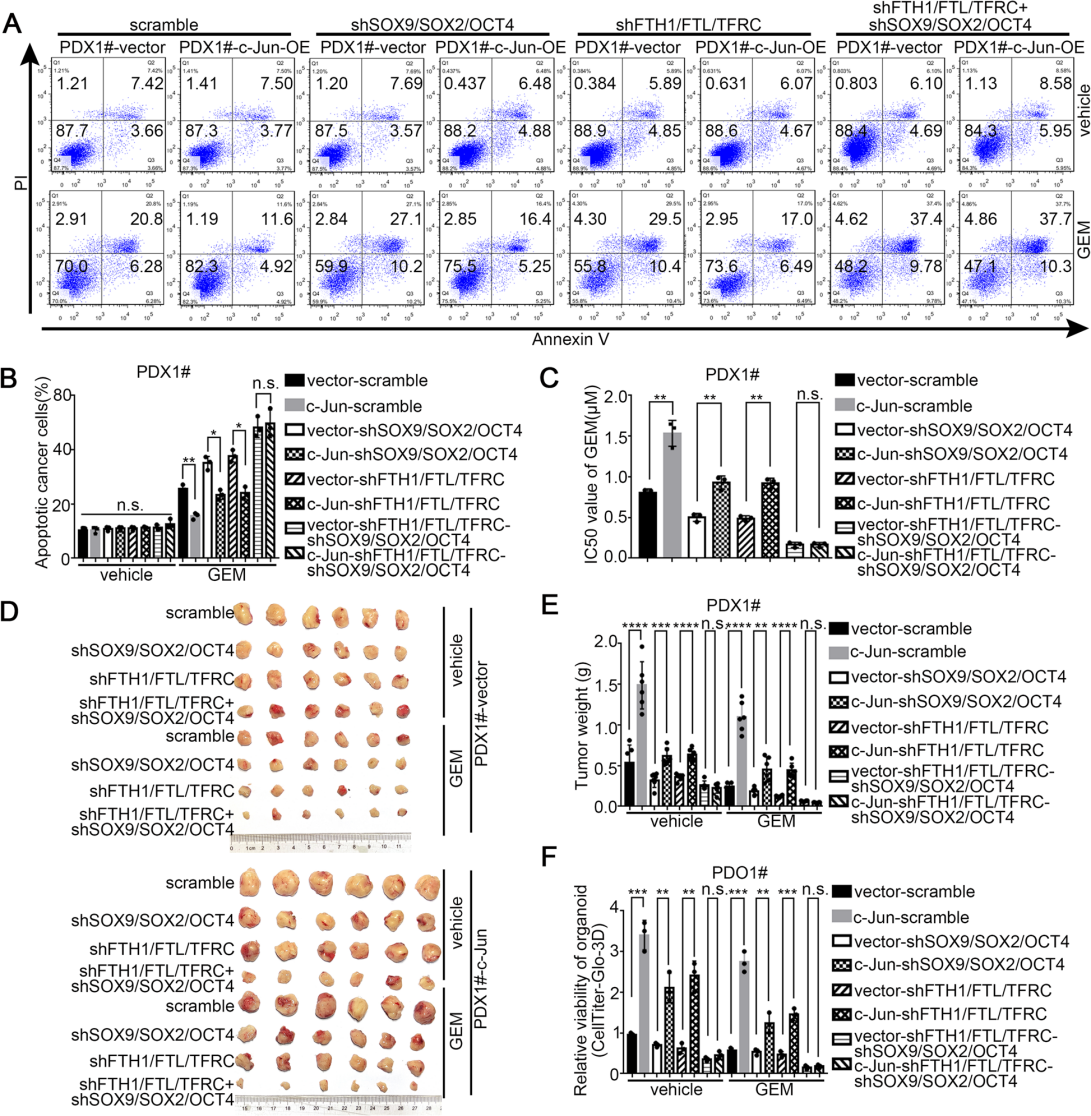
Tumoral c-Jun promotes chemotherapy resistance of GEM through dual pathways including stemness and iron metabolism in PDAC. **A**-**B** Each PDX1# primary cell line was treated with GEM (0.8 μM) for 72 h, and then cellular apoptosis in indicated cell lines were analyzed by flow cytometry. PBS was used as vehicle control. The representative dot plots were shown (**A**) and statistical analysis was shown (**B**). **C** The IC50 value of gemcitabine in indicated cell lines were determined by CCK-8. **D**-**E** Indicated PDX1# primary cell line was subcutaneously transplanted into BALB/C-nude mice and then GEM was administrated. Representative images of tumors were shown (**D**) and tumor weights were monitored (**E**). **F** Indicated PDO1# line was treated with GEM (0.8 μM) for 72 h, and then cellular viability in indicated PDO line was analyzed by CellTiterGlo-3D assay. PBS was used as vehicle control. Statistical analysis of organoid viability was shown. All experiments were repeated three times independently. Paired Student’s t-test were used for in vitro experiments. Un-paired Student’s t-test were used for in vivo experiments

To further determine whether c-Jun promotes PDAC chemotherapy resistance to GEM through up-regulating stemness and iron metabolism genes, in vitro and in vivo gene depletion assays were conducted using PDX cell lines and organoids expressing shRNAs targeting stemness genes (SOX9/SOX2/OCT4), shRNAs targeting iron metabolism genes (FTH1/FTL/TFRC) or the corresponding scrambled shRNA. As shown in Fig. 9A-C, the results of apoptosis assays and CCK8 assays in these PDX cell lines showed that simultaneous depletion of stemness genes (SOX9/SOX2/OCT4) and iron metabolism genes (FTH1/FTL/TFRC) all together could fully abrogate the GEM resistance of PDAC induced by c-Jun. We also observed that the c-Jun-induced GEM resistance in organoids was fully blocked once both stemness and iron metabolism genes were knocked down (Fig. 9D-E). In the orthotopic xenograft tumor mouse models established with PDX cell lines, animals subcutaneously implanted with PDX-vector-shSOX9/SOX2/OCT4-shFTH1/FTL/TFRC or PDX-c-Jun-OE-shSOX9/SOX2/OCT4-shFTH1/FTL/TFRC cell lines exhibited comparable tumor burdens and tumor weights after GEM treatment (Fig. 9F-G). Taken together, our data supported the idea that tumoral c-Jun upregulation increases the expression of stemness genes (SOX9/SOX2/OCT4) and iron metabolism genes (FTH1/FTL/TFRC), which further promotes GEM resistance in PDAC.

### Irbesartan significantly suppresses cancer stemness and iron metabolism in PDAC

**Fig. 10:**
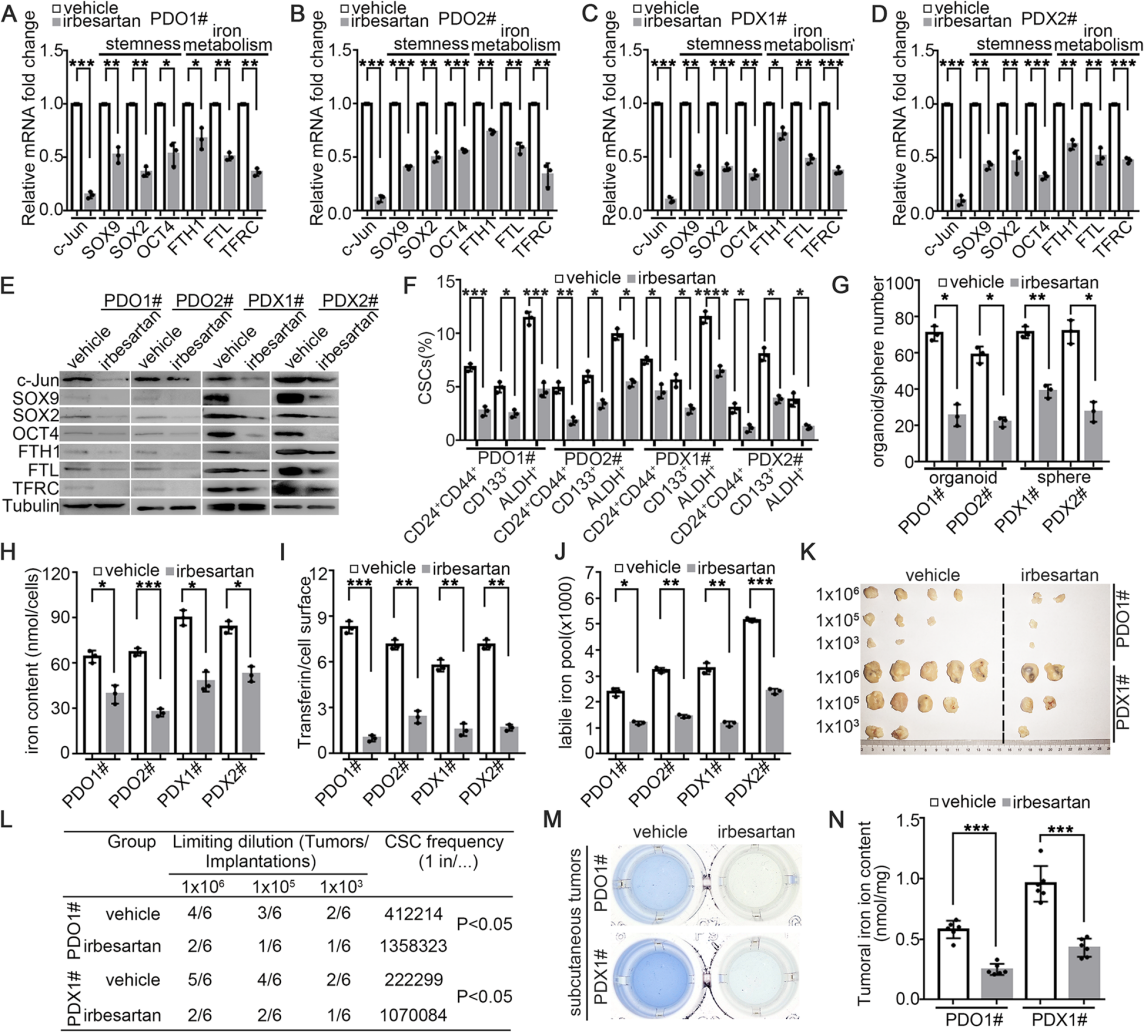
Irbesartan significantly suppressed cancer stemness and iron metabolism in PDAC. **A**-**D** Q-PCR for c-Jun/SOX9/SOX2/NANOG/OCT4/FTH1/FTL/TFRC were performed in indicated PDX and PDO lines treated with irbesartan (1 μM, 72 h). **E** Western blot for c-Jun/SOX9/SOX2/NANOG/OCT4/FTH1/FTL/TFRC were performed in indicated PDX and PDO lines treated with irbesartan (1 μM, 72 h). **F** Percentage of CSCs (CD24^+^CD44^+^ cells, ALDH^+^ cells and CD133^+^ cells) in indicated PDX and PDO lines treated with irbesartan (1 μM, 72 h). **G** Sphere/organoid number in indicated PDX and PDO lines treated with irbesartan (1 μM, 72 h). **H**-**J** Iron metabolism level in indicated PDX and PDO lines treated with irbesartan (1 μM, 72 h) were determined. Statistical analysis of cellular iron content (**H**), transferrin uptake capacity (**I**) and LIP (**J**) was shown. **K**-**L** In vivo limited dilution assays for indicated cell lines were performed. Representative tumor incidence and CSC probabilities were shown. **M**–**N** Tumor iron content each group in mice of Fig. 7K was measured. Representative images of iron assay each group (**M**) and statistical analysis (**N**) were shown. All experiments were repeated three times independently. Paired Student’s t-test were used for in vitro experiments. Un-paired Student’s t-test were used for in vivo experiments

To further explore whether irbesartan decreases GEM resistance in PDAC by suppressing the c-Jun-stemness/iron metabolism axis, we evaluated the expression of SOX9, SOX2, OCT4, FTH1, FTL and TFRC in a 2D PDX primary cell line and a 3D organoid model treated with irbesartan. As shown in Fig. 10A-E, irbesartan significantly suppressed the mRNA and protein expression of stemness and iron metabolism-related genes and markedly reduced the level of tumoral c-Jun. Irbesartan also resulted in a significant decrease in CSC subsets and the sphere/organoid formation capacity (Fig. 10F-G). Furthermore, obvious decreases in the iron content, transferrin uptake capacity and labile iron pool were observed in PDAC samples treated with irbesartan (Fig. 10H-J). To further confirm the effects of irbesartan on stemness and iron metabolism in PDAC, in vivo limited dilution assays and iron colorimetric assays were performed. As expected, irbesartan significantly decreased the tumor incidence (Fig. 10K-L) and tumor iron content (Fig. 10M-N) compared with those in the vehicle group. Moreover, we evaluated the effects of irbesartan in the KPC mouse model and found that irbesartan significantly reduced c-Jun expression, Hippo/YAP1 activity, and the expression levels of stemness and iron metabolism genes in pancreatic tumors in the KPC mouse model (Fig. S15). Overall, our study clarified that irbesartan could overcome GEM resistance in PDAC via inhibition of the c-Jun-stemness/iron metabolism axis.

### The tumoral c-Jun expression level predicts the efficacy of chemotherapy and irbesartan plus chemotherapy regimens in human PDAC patients

**Fig. 11:**
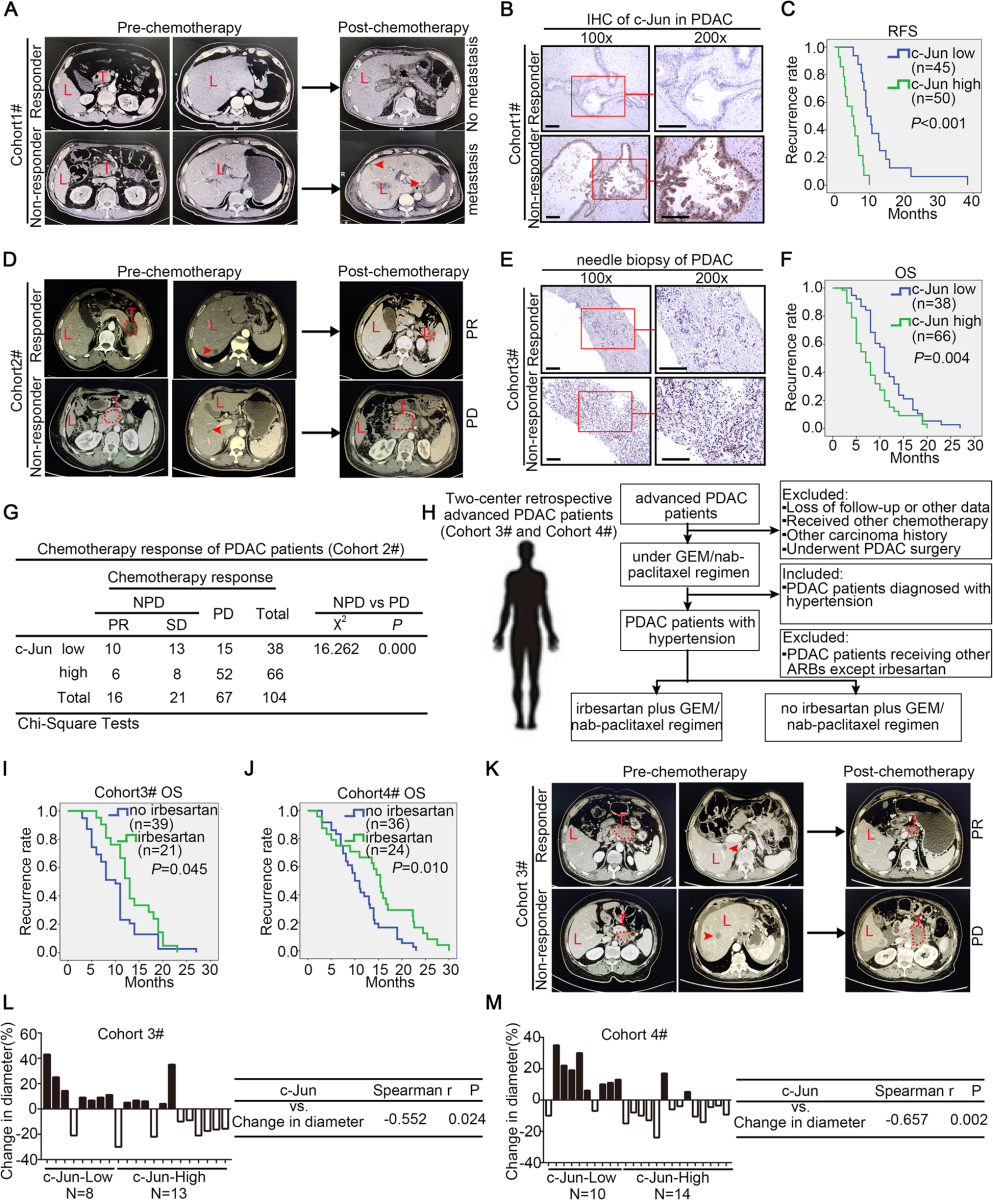
Tumoral c-Jun expression level predicts the efficacy of chemotherapy and irbesartan plus chemotherapy regimens in human PDAC patients. **A**-**C** PDAC patients in Cohort1# received post-operative chemotherapeutic regimen of gemcitabine/nab-paclitaxel. Representative CT-scanning images of the two patients were shown (**A**), representative IHC staining of c-Jun from the two patients were shown (**B**) and the association of c-Jun expression with RFS rate in PDAC patients were analysed (**C**). Red arrows marked the metastases. L, liver; T, tumour. **D**-**G** Advanced PDAC patients in Cohort2# received GEM/nab-paclitaxel chemotherapy. Representative CT-scanning images of the responder and non-responder were shown (**D**), representative IHC staining of c-Jun from the pancreas needle biopsy tissues were shown (**E**), Kaplan–Meier OS for different levels of c-Jun of advanced PDAC patients based on the log-rank statistic test (**F**). Red arrows marked the metastases. Red dash lines indicated the pancreatic tumours. L, liver; T, tumour. Chemotherapy response data of all advanced patients in Cohort2# were collected and the relationship between chemotherapy response and tissue c-Jun expression level were analyzed by Chi-Square T test (**G**). PR, partial remission; SD, stable disease; PD, progressed disease. **H** Flowchart of patients enrolling in Cohort3# and Cohort4#. **I**-**J** Kaplan–Meier OS for advanced PDAC patients receiving GEM/nab-paclitaxel and irbesartan plus GEM/nab-paclitaxel regimen based on the log-rank statistic test in Cohort3# (**I**) and Cohort4# (**J**). **K** Representative CT-scanning images of the responder and non-responder for irbesartan plus GEM/nab-paclitaxel regimen in Cohort3# were shown. Red arrows marked the metastases. Red dash lines indicated the pancreatic tumours. L, liver or lung; T, tumour. **L**-**M**The change in tumor diameter for PDAC patients with irbesartan plus GEM/nab-paclitaxel therapy in Cohort 3# and Cohort4#, those with increased tumor diameter were colored by red (L, left; M, left). Quantitative correlation between the change in tumor diameter and tumoral c-Jun expression levels based on Spearman’s rank correlation test (L, right; M, right)

To further validate the above findings, we first collected and analyzed the IHC and clinical data from PDAC patients in Cohort 1#, who received the current GEM plus nab-paclitaxel regimen as postoperative adjuvant chemotherapy. As shown in Figs. 11A-C, early liver metastasis of PDAC and a decreased relapse-free survival time were found in PDAC patients with high c-Jun expression. Additionally, we collected needle biopsy PDAC tissue samples from 104 patients with advanced PDAC in Cohort2# who had received GEM plus nab-paclitaxel chemotherapy. Similarly, PDAC patients with low c-Jun levels demonstrated a better chemotherapy response and longer overall survival time, and patients with high c-Jun levels demonstrated a worse chemotherapy response and shorter overall survival time (Fig. 11D-G). In summary, these results were highly consistent with our in vitro and in vivo studies showing that tumoral c-Jun expression is negatively correlated with the response to chemotherapy in PDAC patients.

Next, to further confirm the effects of irbesartan on enhancing the efficacy of chemotherapy, we assessed the survival of patients with advanced PDAC in two-center independent retrospective cohorts (Fig. 11H). The Cohort3# contained 60 patients with advanced (stage III/IV) PDAC who received GEM/nab-paclitaxel chemotherapy at Tianjin Medical University Affiliated Cancer Institute and Hospital and all the patients were diagnosed with hypertension. Patients receiving other ARBs were excluded. In this cohort, 21 PDAC patients received irbesartan treatment during chemotherapy. In the stratified analysis, the Kaplan–Meier curves showed that irbesartan treatment was significantly associated with a longer overall survival time (Fig. 11I). Similar results were obtained in Cohort4#, which contained 60 patients with advanced (stage III/IV) PDAC who received GEM/nab-paclitaxel chemotherapy at Tongliao City Hospital and were also diagnosed with hypertension (Fig. 11J). In addition, Fig. 11K shows the change in pancreatic tumor burden in two representative patients receiving the irbesartan plus GEM/nab-paclitaxel regimen. We observed a significant negative correlation between the relative increase in the tumor diameter and the tumoral c-Jun expression level in PDAC patients receiving the irbesartan plus GEM/nab-paclitaxel regimen in Cohort3# and Cohort4# (Fig. 11M).

Taken together, these preclinical results further confirmed that PDAC patients with high expression levels of c-Jun showed less potential benefit from GEM-based chemotherapy, while those with high c-Jun expression might benefit from an irbesartan plus GEM-based combinational regimen. Thus, irbesartan has the potential to become an important anti-tumor drug and be used to synergistically enhance the efficacy of GEM-based chemotherapy in PDAC.

## Discussion

There is convincing evidence that PDOs cultured in a 3D matrix are superior to 2D cell lines and PDXs in terms of stability and fidelity as a drug screening model [33]. In our study, we established a biobank of genetically distinct human PDAC organoid lines and showed that the morphology, genetic background and drug response of the PDAC organoids were significantly correlated with those observed in the corresponding primary PDAC tissues and patients. Our data confirmed the results of recent studies [9, 12, 33–36]. Taking advantage of the integrated robotic screening platform, we screened a total of 1304 FDA-approved drugs. The candidate drug, GEM, and the combined regimen were tested. To the best of our knowledge, our research is the first to use GEM-resistant PDOs for large scale drug screening. Among the drugs we identified, irbesartan had the most significant effect on reducing GEM chemoresistance, which was further validated using a PDX mouse model, PDOs and PDO xenografts. All three models retained the genetic alterations and histopathological features of the primary tumors [37, 38]. Actually, several previous researches also pointed out that other member of ARB families demonstrated synergistic inhibitory effects with chemotherapy on PDAC. A retrospective cohort led by Y Nakai et al. reported that the ACEIs/ARBs in combination with gemcitabine might improve clinical outcomes in patients with advanced pancreatic cancer [39]. Besides, Ryuichi Noguchi et al. found that losartan could also sensitize PDAC to gemcitabine regimen via anti-angiogenic activities [40]. In pancreatic cancer, cancer cell-derived cytokines activate pancreatic stellate cells (PSCs) forming the CAF phenotype and contributing to extensive pancreatic desmoplasia. The stroma in PDAC contributes to poor vascularisation and high intratumoural pressure that decreases drug diffusion [41]. A parallel preclinical trial using patient-derived xenograft models of PDA, showed that exposure to nab-paclitaxel collapsed the PDA stroma, increasing the intratumor concentration of gemcitabine by approximately three folds [42]. Feng, R et al. and R Alvarez et al. proved the stromal disrupting effects of nab-paclitaxel in PDAC [43, 44]. In addition, the AT1 inhibitor (AT1 blocker, ARB) losartan can reduce PSC activation, desmoplasia and solid stress in PDAC which further potentiates chemotherapy [28, 45]. More importantly, we also found that irbesartan can also synergistically sensitize PDAC to the current standard chemotherapy regimen (GEM/nab-paclitaxel therapy) in KPC genetically engineered mouse models, confirming the promising clinical application value of the irbesartan plus GEM/nab-paclitaxel regimen. Considering that the KPC genetically engineered mouse model also highly mimicked the extensive demoplastic status of PDAC, we hypothesized that the AT1 inhibitor irbesartan can sensitize PDAC to chemotherapy by affecting both stromal and tumor cells in KPC genetically engineered mouse model. On the one hand, irbesartan suppressed the Hippo/YAP1/c-Jun axis and further directly enhanced the responsiveness of tumour cells to chemotherapy in KPC genetically engineered mouse model; on the other hand, irbesartan also reduced desmoplasia and solid stress in PDAC tumors and further increased the therapeutic drug diffusion. Taken together, our findings indicated that gemcitabine, nab-paclitaxel and irbesartan combination therapy showed better synergistic effects than the monotherapies in the KPC genetically engineered mouse model.

Irbesartan is an angiotensin II type 1 receptor (AT1R) blocker (ARB) that is extensively used for the treatment of hypertension. Recent studies have also explored some of the effects of irbesartan on tumor suppression. Kensuke et al. demonstrated that Irbesartan suppressed MCP-1 production, and downregulated the expression of type 1 collagen and matrix metalloproteinase 9, which inhibited the development of fibrosis and tumours [46]. Moreover, Irbesartan reduced the expression of survivin, and increased the level of cleaved caspase 3 [47]. However, the role of irbesartan in reducing chemotherapy resistance in PDAC has not been reported.

To explore the target of irbesartan, we performed RNA-sequencing and found that c-Jun, a canonical member of the AP-1 family, was significantly repressed by irbesartan in PDAC. Further GSEA analysis and in vitro/ *vivo* assays indicated that irbesartan significantly suppressed c-Jun expression by inhibiting the activation of the Hippo/YAP1 signaling pathway. Angiotensin II type 1 receptor (AT1R), is a G protein-coupled receptor (GPCR) that is thought to modulate the Hippo/YAP pathway [48]. Recent research has revealed that losartan, another ARB, attenuates cholangiocarcinoma cell growth by inhibiting the oncogenic activity of YAP1, which also supports our findings [49]. Stratification of patients based on the heterogeneity of PDAC will facilitate a personalized approach to optimally treat each patient. Based on several large-scale transcriptomic profiling studies recently reviewed in detail elsewhere, two major subtypes of malignant epithelial cells have been defined, and are referred to as classical (CLA) and basal-like (BL) (or squamous) [50–54]. These two clinically relevant PDAC subtypes differ not only in their transcriptional and histological profiles but also in their response to therapy. The basal-like subtype is associated with poor differentiation, a worse outcome, and resistance to therapy, while the classical subtype tends to be more differentiated and to show a better outcome and chemo-responsiveness. While the majority of tumors are predominantly one of these two defined subtypes, individual tumors can also present a ‘mixed’ subtype phenotype containing neoplastic cells with characteristics of each of the subtypes. Lineage transcription factors (TFs) are the major determinants that shape PDAC subtype identity. Recently, Mengyu Tu et al.also uncovered TF-heterogeneity in the JUN/AP-1 family [55]. After analyzing the gene expression patterns in tumors from a KrasG12D; p53R172H; Pdx-Cre (KPC) genetically engineered mouse model (GEMM), orthotopic, patient-derived xenografts (PDXs), and PDAC patient specimens, Mengyu Tu et al. found that high expression of c-Jun/AP-1 was linked to poorly differentiated/basal subtype lesions, which predicted an overall unfavorable prognosis and resistance to therapy in PDAC. In addition, another recent study has analyzed the expression profiles of the distinct molecular subtypes of human PDAC from the TCGA database and found that tumors of the basal-like/squamous subtype exhibited elevated expression of genes that are known to be associated with YAP1 activation [56]. Moreover, expression of the YAP1 activation signature was significantly correlated with that of the squamous subtype signature, underscoring the tight association between YAP1 activation and basal-line/squamous subtype tumors [56]. Thus, PDAC patients with the basal-like/squamous subtype tend to harbour high expression levels of c-Jun/AP-1, YAP1 activation signature molecules, and poor responsiveness to chemotherapy, and patients are suitable for treatment with irbesartan plus chemotherapy regimens in the clinic.

As organoids are well suited for in vitro manipulations, we also developed PDO lines with CRISPR/Cas9 genome editing to explore the target and mechanism of irbesartan in reducing GEM resistance. Among chemoresistance-related pathways, the stemness maintenance and iron metabolism pathways were the most significantly activated by c-Jun. SOX9/SOX2/OCT4 are the key regulators of tumor stemness maintenance [57–59]. Cheng-Chi Chang et al. reported that c-Jun directly activates the transcription of the pluripotency genes SOX2 and OCT4 in head and neck squamous cancer [60]. Jiayan Lang et al. reported that PDAC cells showed elevated iron metabolism levels compared to adjacent normal cells, and that the iron chelator deferoxamine (DFO) significantly reduced the tumour burden of PDAC by decreasing iron metabolism [61]. In addition, Badgley, M. A. found that cysteine depletion induced pancreatic tumor ferroptosis [62]. Several key genes control the regulation of cellular iron homeostasis, such as TFR, FTH1 and FTL etc. TFR is essential for cellular iron uptake via its interaction with the extracellular transferrin-Fe^3+^ complex. In complex, FTH1 and FTL form ferritin, which is responsible for cellular Fe^3+^ storage [63]. Giovanna Marziali et al. found that the upstream region of the transcription start site (TSS) of the TFRC gene contains an overlapping consensus recognition sequence for AP1/CREB/ATF transcription factors (TGACGCA) [64]. Consistent with this finding, we finally confirmed that c-Jun directly bound to the promoter regions of SOX9/SOX2/OCT4/FTH1/FTL/TFRC to activate their transcription in PDAC.

According to our chemotherapy response analysis using one retrospective cohort in Fig. 11G, approximately 77.6% (52/67) of patients with advanced PDAC exhibiting progressive disease (PD) had high expression levels of c-Jun, which indicated that patients with chemotherapy-resistant PDAC and high levels of c-Jun could be screened for suitability for irbesartan plus chemotherapy combination regimen in the future.

## Conclusion

**Fig. 12:**
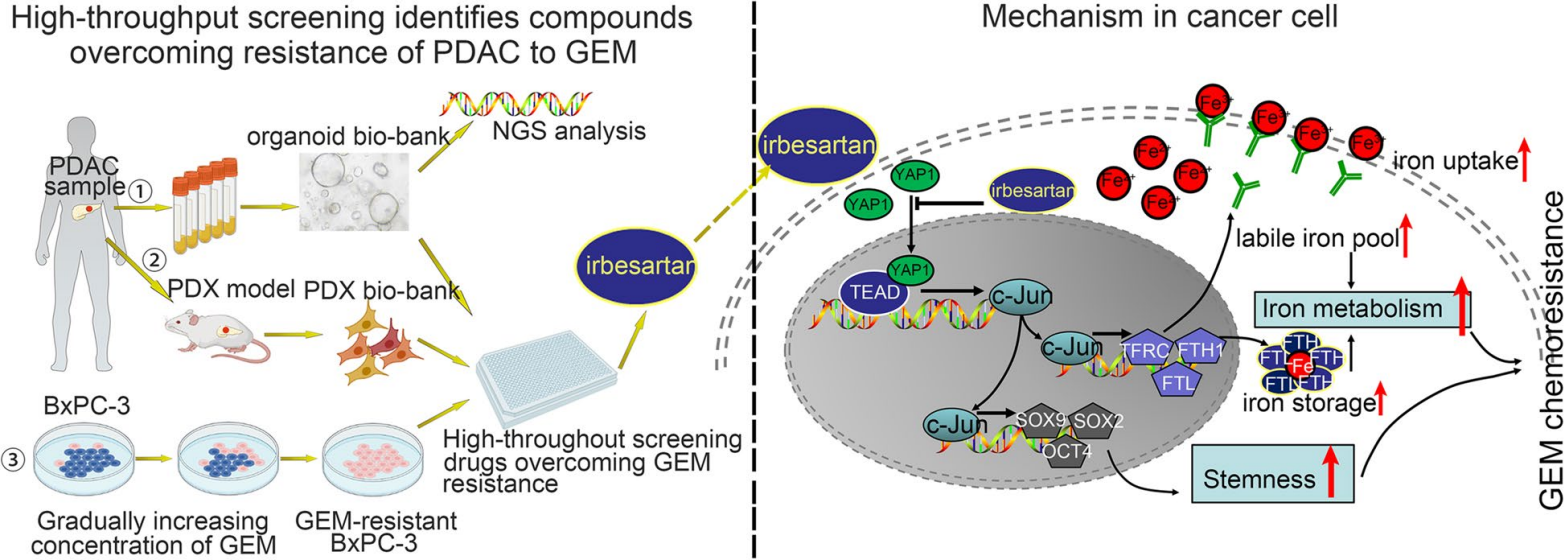
Schematic illustration for the whole project

In conclusion, we established a biobank of heterogeneous human PDAC organoids with different degrees of chemosensitivity to GEM. High-throughput drug screening with a panel of 1304 FDA-approved drugs identified several compounds that effectively reduced GEM chemoresistance in PDAC in organoid cultures. By utilizing genome edited PDO lines, PDOs xenografts and PDX mouse model, the effect of irbesartan on reducing chemosensitivity, as well as its target and mechanism, were validated. Irbesartan effectively reduced chemoresistance in PDAC by targeting the Hippo/YAP1/c-Jun-stemness/iron metabolism axis. Based on the promising effects of irbesartan, we are designing an investigator-initiated phase II clinical trial on the efficacy and safety of irbesartan plus GEM/nab-paclitaxel chemotherapy in the treatment of patients with advanced (stage III/IV) PDAC and are hopeful that we will observe patient benefits (Fig. 12).

## Supplementary Information

**Additional file 1: Figure S1.** Related to Fig. 1. The effects of combination of GEM and the candidates’ drugs (irbesartan, Dapansutrile, Pemigatinib and Dauricine) on GEM-resistant PDO1# (A-D), PDO2# (E-H) and PDO7# (I-L) lines by CelltiterGlo-3D assays. Paired Student’s t-test were used for in vitro experiments. **Figure S2.** Related to Fig. 2. (A-B) The relative area of organoids per group in Fig. 2C, F were measured by Image J software. (C-D) The fluorescence intensity of Caspase3/7 of organoids per group in Fig. 2C, F were determined by software Image J. (E-F) The mean intensity of Ki67 of organoids per group. (G) Mice body weight per group in Fig. 2I-K were monitored. All experiments were repeated three times independently. Paired Student’s t-test were used for in vitro experiments. Repeated measure two-way ANOVA (time x mice body weight) and post-hoc analyses were used for test mouse body weight between groups. **Figure S3.** The effects of irbesartan were validated in another 9 patient-derived organoids (PDOs) subcutaneous tumor models in vivo and the tumor growth curves were plotted. Repeated measure two-way ANOVA (time × tumor volume) and post-hoc analysis were used for test tumor growth between groups. **Figure S4.** The effects of irbesartan were validated in 10 patient-derived xenografts (PDXs) subcutaneous tumor models in vivo and the tumor growth curves were plotted. Repeated measure two-way ANOVA (time × tumor volume) and post-hoc analysis were used for test tumor growth between groups. **Figure S5.** Irbesartan could significantly reverse GEM resistance in GEM-resistant BxPC-3 cell lines. **Figure S6.** The expression pattern of c-Jun and its clinical significance in PDAC. **Figure S7.** The effects of irbesartan on inhibiting c-Jun expression were validated in 10 patient-derived organoids (PDOs) tumors from Fig.2K and Fig. S3 via western blot and q-PCR. **Figure S8.** The effects of irbesartan on inhibiting c-Jun expression were validated in 10 patient-derived xenografts (PDXs) subcutaneous tumors from Fig.S5, another GEM-resistant BxPC-3 orthotopic tumors from Fig. S6 via western blot and q-PCR. **Figure S9.** Related to Fig. 4. **Figure S10.** Related to Fig. 6. **Figure S11.** Tumoral c-Jun induces chemotherapy resistance of GEM in 2D PDAC models in vitro. **Figure S12.** Tumoral c-Jun is positively correlated with stemness profiles in PDAC. **Figure S13.** Data related to Fig. 7. **Figure S14.** Tumoral c-Jun positively regulates the expression of stemness genes (SOX9/SOX2/OCT4) and iron metabolism genes (FTH1/FTL/TFRC) in PDAC. **Figure S15.** Data related to Fig. 10. **Supplementary table 2. **Detailed information of patients whose specimens were used for establishment of PDO models and in vivo PDX mouse models. **Supplementary table 5. **Correlation of c-Jun expression to clinicopathological features in PDAC. **Supplementary table 6.** Univariate and multivariate Cox proportional hazards analysis of clinicopathological factors for median overall survival and relapse free survival. **Supplementary table 10. **detailed information of shRNA oligos sequences for stable knockdown cells (the following oligos sequences were shown as: overhang-target-loop-antisense). **Supplementary table 11. **detailed information of antibodies used in this study. **Supplementary table 12. **detailed information of primers used for RT-PCR and Ch-IP in this study. **Supplementary table 13.** detailed information of sequences of the plasmids used for luciferase analysis (The following DNA sequences were cloned into pGL3.0-basic plasmid).

**Additional file 2: Table S1. **Detailed information of drug library.

**Additional file 3: Table S3. **Cellular inhibitory rate of drug library.

**Additional file 4: Table S4.** RNA sequencing of PDO01 treated with irbesartan.

**Additional file 5: Table S7.** RNA-sequencing of PDX01-vector_c-Jun-OE.

**Additional file 6: Table S8.** RNA-sequencing of PDO04-vector_c-Jun-OE.

**Additional file 7: Table S9.** GSEA analysis by intersection of PDO04-vector_c-Jun and PDX01-vector_-c-Jun.

## Data Availability

All data relevant to the study are included in the article or uploaded as supplementary information.

## Abbreviations

PDAC: Pancreatic ductal adenocarcinoma
KPC: LSL-Kras^G12D/+^, LSL-Trp53^R172H/+^ and Pdx1-Cre
GEM: Gemcitabine
CSCs: Cancer stem cells
PDO: Patient-derived organoid
PDX: Patient-derived xenograft
Ch-IP: Chromatin immunoprecipation
GSEA: Gene set enrichment analysis
