# Supplementary material for: Irbesartan overcomes gemcitabine resistance in pancreatic cancer by suppressing stemness and iron metabolism via inhibition of the Hippo/YAP1/c-Jun axis
Source: J Exp Clin Cancer Res. 2023 May 4;42:111. doi: 10.1186/s13046-023-02671-8 (PMC10157938; doi:10.1186/s13046-023-02671-8)
Supplement: Supplementary file 1 — Additional file 1: Figure S1. Related to Fig. 1. The effects of combination of GEM and the candidates’ drugs (irbesartan, Dapansutrile, Pemigatinib and Dauricine) on GEM-resistant PDO1# (A-D), PDO2# (E-H) and PDO7# (I-L) lines by CelltiterGlo-3D assays. Paired Student’s t-test were used for in vitro experiments. Figure S2. Related to Fig. 2. (A-B) The relative area of organoids per group in Fig. 2C, F were measured by Image J software. (C-D) The fluorescence intensity of Caspase3/7 of organoids per group in Fig. 2C, F were determined by software Image J. (E-F) The mean intensity of Ki67 of organoids per group. (G) Mice body weight per group in Fig. 2I-K were monitored. All experiments were repeated three times independently. Paired Student’s t-test were used for in vitro experiments. Repeated measure two-way ANOVA (time x mice body weight) and post-hoc analyses were used for test mouse body weight between groups. Figure S3. The effects of irbesartan were validated in another 9 patient-derived organoids (PDOs) subcutaneous tumor models in vivo and the tumor growth curves were plotted. Repeated measure two-way ANOVA (time × tumor volume) and post-hoc analysis were used for test tumor growth between groups. Figure S4. The effects of irbesartan were validated in 10 patient-derived xenografts (PDXs) subcutaneous tumor models in vivo and the tumor growth curves were plotted. Repeated measure two-way ANOVA (time × tumor volume) and post-hoc analysis were used for test tumor growth between groups. Figure S5. Irbesartan could significantly reverse GEM resistance in GEM-resistant BxPC-3 cell lines. Figure S6. The expression pattern of c-Jun and its clinical significance in PDAC. Figure S7. The effects of irbesartan on inhibiting c-Jun expression were validated in 10 patient-derived organoids (PDOs) tumors from Fig.2K and Fig. S3 via western blot and q-PCR. Figure S8. The effects of irbesartan on inhibiting c-Jun expression were validated in 10 patient-derived xenografts (PDXs [file 13046_2023_2671_MOESM1_ESM.docx]

**Supplementary figures and figure legends**

**
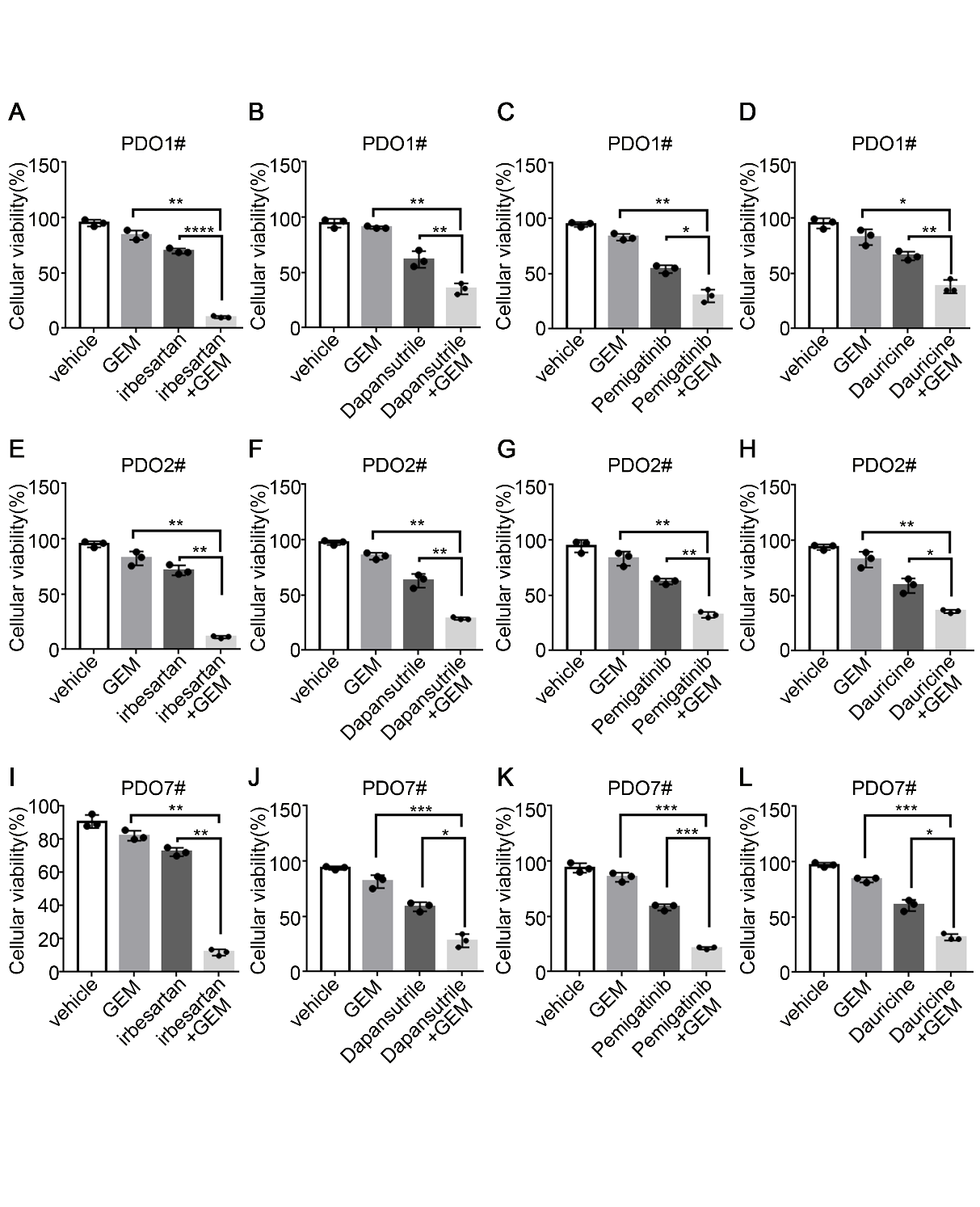
**

**Figure S1: Related to Figure1.** The effects of combination of GEM and the candidates’ drugs (irbesartan, Dapansutrile, Pemigatinib and Dauricine) on GEM-resistant PDO1# (A-D), PDO2# (E-H) and PDO7# (I-L) lines by CelltiterGlo-3D assays. Paired Student’s t-test were used for in vitro experiments.

**
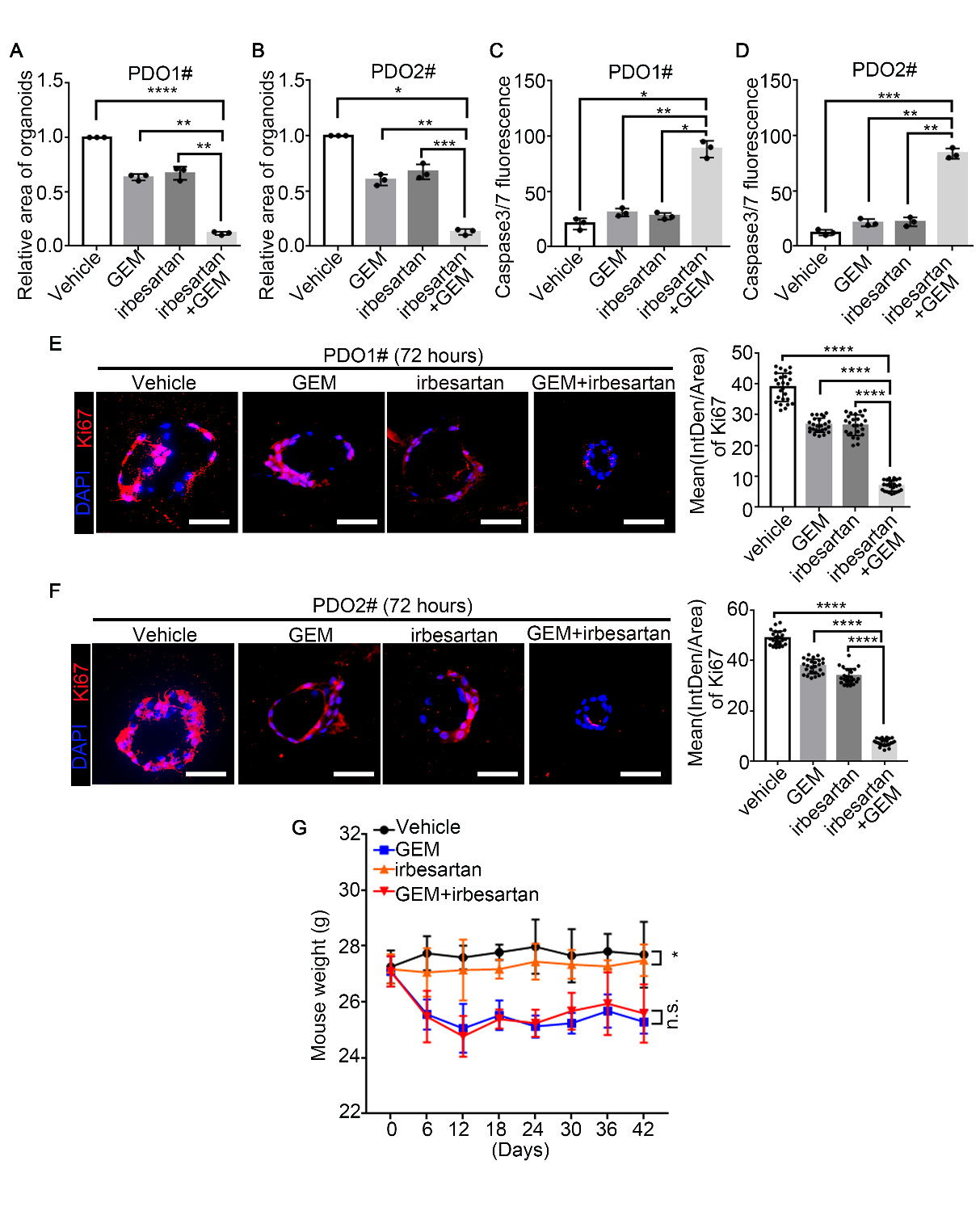
**

**Figure S2: Related to Figure 2.** (A-B) The relative area of organoids per group in **Figure.2C, F** were measured by Image J software. (C-D) The fluorescence intensity of Caspase3/7 of organoids per group in **Figure.2C, F** were determined by software Image J. (E-F) The mean intensity of Ki67 of organoids per group. (G) Mice body weight per group in **Figure 2I-K** were monitored. All experiments were repeated three times independently. Paired Student’s t-test were used for in vitro experiments. Repeated measure two-way ANOVA (time x mice body weight) and post-hoc analyses were used for test mouse body weight between groups.

**
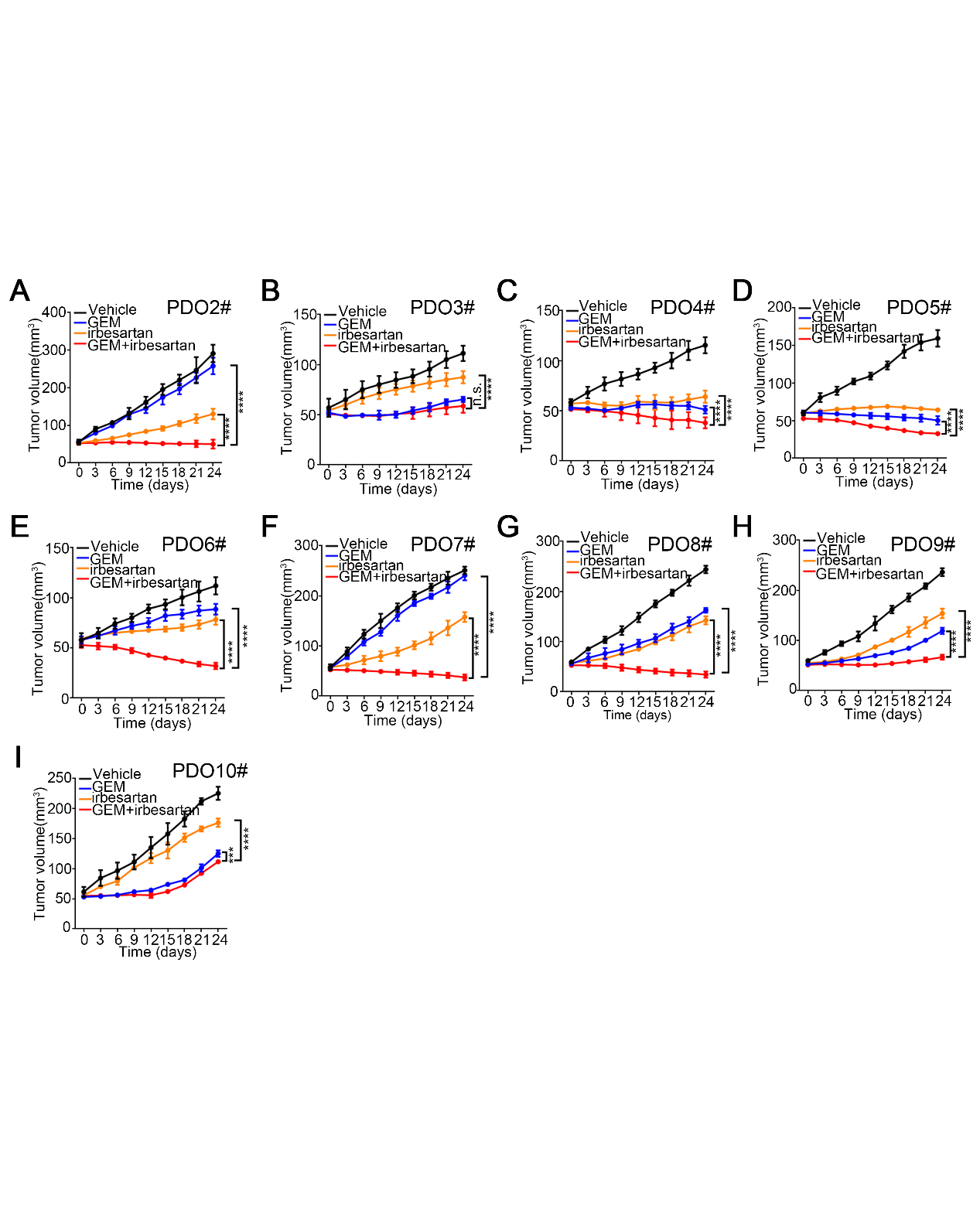
**

**Figure S3: The effects of irbesartan were validated in another 9 patient-derived organoids (PDOs) subcutaneous tumor models in vivo and the tumor growth curves were plotted.** Repeated measure two-way ANOVA (time × tumor volume) and post-hoc analysis were used for test tumor growth between groups.

**
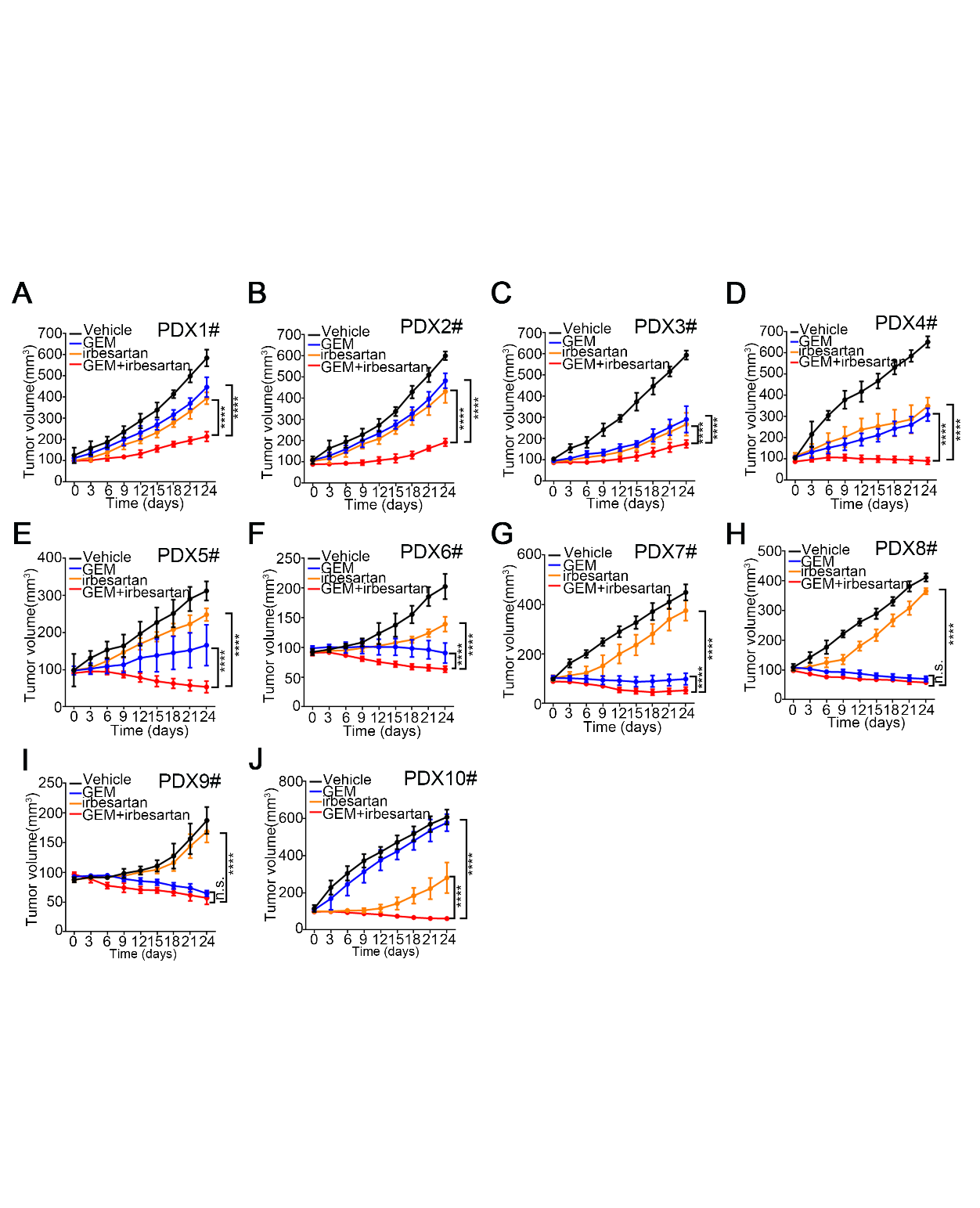
**

**Figure S4: The effects of irbesartan were validated in 10 patient-derived xenografts (PDXs) subcutaneous tumor models in vivo and the tumor growth curves were plotted.** Repeated measure two-way ANOVA (time × tumor volume) and post-hoc analysis were used for test tumor growth between groups.

**
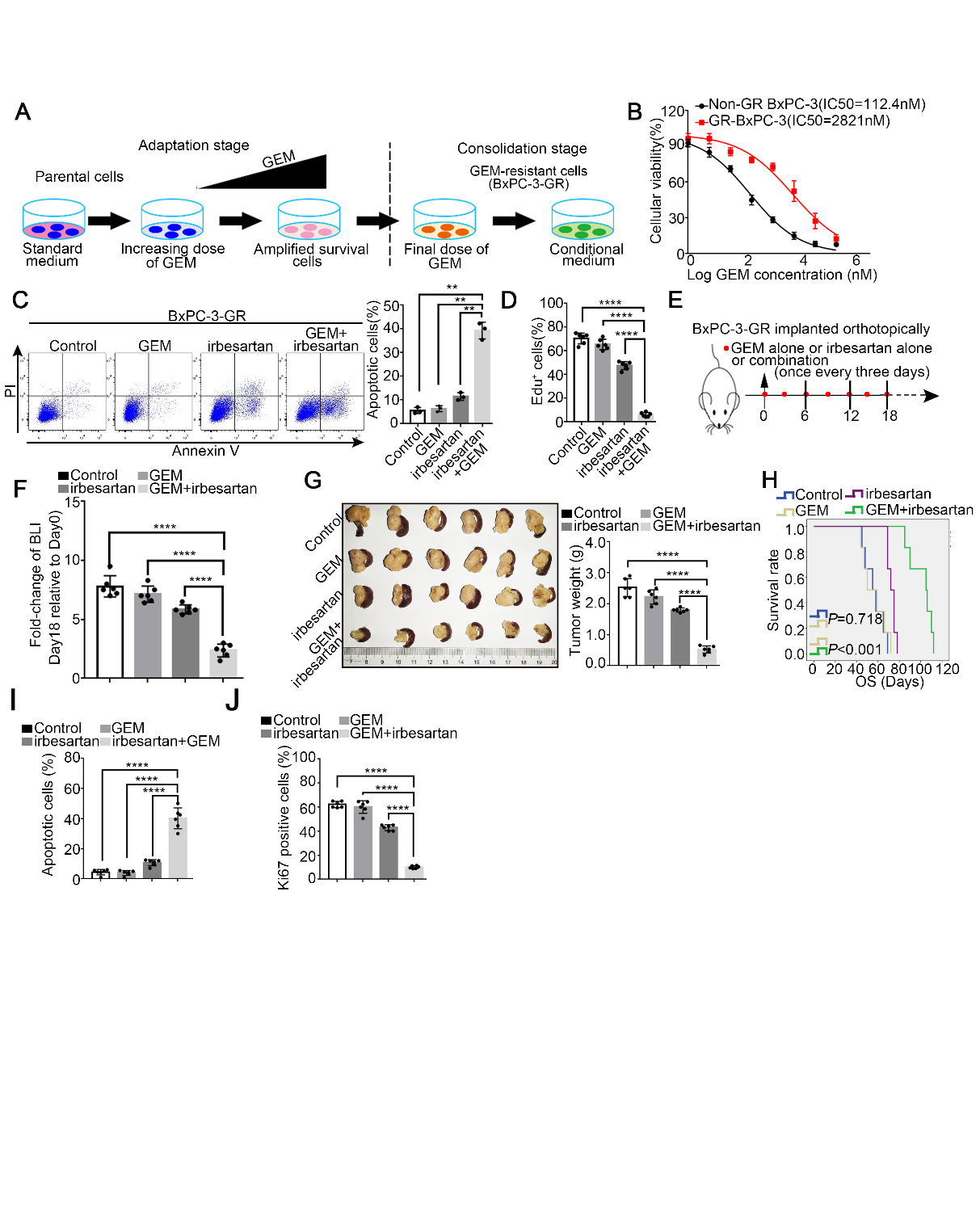
**

**Figure S5: Irbesartan could significantly reverse GEM resistance in GEM-resistant BxPC-3 cell lines.** (A) The schematic illustration of the establishment process for the gemcitabine-resistant BxPC-3 cell lines. (B)The IC50 value of gemcitabine in GEM-non-resistant/resistant BxPC-3 cell lines were determined by CCK-8 assay. (C) BxPC-3-GR cell lines were treated with vehicle, GEM (1.0 μM), irbesartan (1 μM) and GEM plus irbesartan for 72 hours and then cellular apoptosis was detected by flow cytometry for Annexin V/PI. The representative dot plots were shown (left) and the percentage of apoptotic cells was analyzed (right). (D) BxPC-3-GR cell lines were treated with vehicle, GEM (1.0 μM), irbesartan (1μM) and GEM plus irbesartan for 72 hours and then cellular proliferation was detected by EdU staining. The percentage of EdU positive cells was analyzed. (E) Schematic illustration for in vivo therapeutic experiments in an orthotopic mice model. (F) Statistical analysis of the fold change of BLI after drug treatment (BLI on day 18 to BLI on day 0) ( n=6 per group). (G) Representative pancreatic tumor images per group at the experimental ending were shown (left) and tumor weight was determined (right). (H) Kaplan-Meier survival curves with log-rank test were used to analyze the effects after drug treatment in another cohort (n=6). (I-J) The apoptotic and proliferative level of pancreatic tumor in mice were evaluated by TUNEL staining (I) and Ki67 staining (J). All experiments were repeated three times independently. Paired Student’s t-test were used for in vitro experiments. Un-paired Student’s t-test were used for in vivo experiments.

**
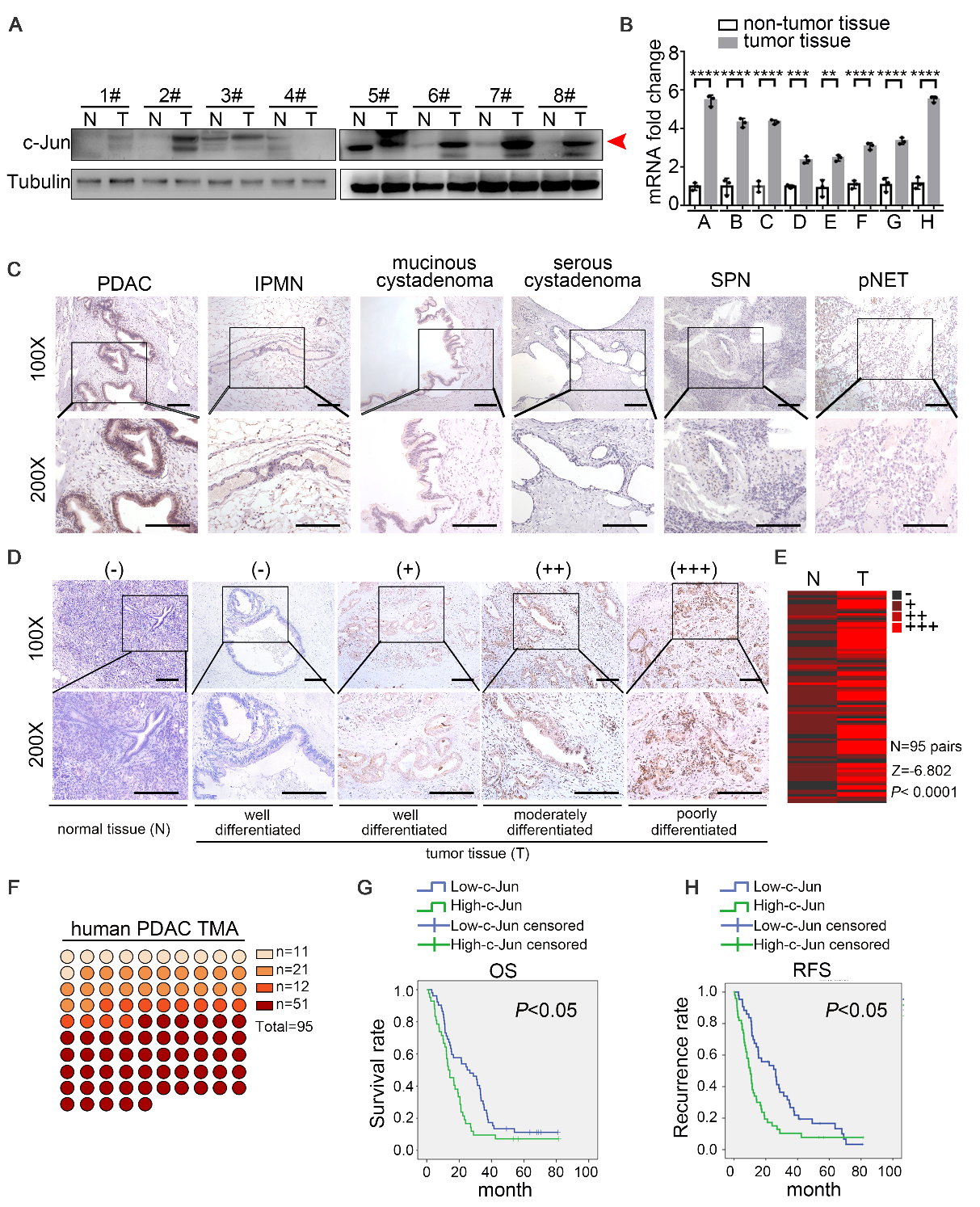
**

**Figure S6: The expression pattern of c-Jun and its clinical significance in PDAC.** Two independent PDAC cohorts were used to evaluate the mRNA and protein expression of c-Jun in panel A and panel B. (A) Eight pairs of fresh PDAC tumor tissues (T) and non-tumor tissues (N) were subjected to western blotting to compare the protein expression levels of c-Jun. Tubulin was used as loading control. (B) Another eight pairs of fresh PDAC tumor tissues and adjacent non-tumor tissues were subjected to RT-PCR analysis to compare the expression levels of c-Jun. Actin was used as loading control. Paired student’s t-test was used for statistical analysis. (C) IHC staining was performed to determine the expression of c-Jun in PDAC and benign pancreatic tumors including serous cystadenoma, mucinous cystadenoma, intraductal papillary mucinous neoplasm (IPMN), solid pseudopapillary neoplasm of the pancreas (SPN) and pancreatic neuroendocrine tumor (pNET). (D-E) Sections of PDAC tissues were used to analyze the expression levels of c-Jun in tumor tissues (T) and the corresponding non-tumor tissues (N). Representative images were shown for absent (-), weak (+), moderate (++), and strong (+++) expression of c-Jun in IHC staining of PDAC tumor and non-tumor tissues (D). The differential expression of c-Jun in 95 pairs of tumor and non-tumor tissues was shown in a heat map and was statistically analyzed by Wilcoxon signed rank tests (E). (F) The distribution of IHC results in 95 PDAC tissues. (G-H) Kaplan–Meier OS (G) and RFS (H) for different levels of c-Jun based on the log-rank statistic test (*P*<0.05).

**
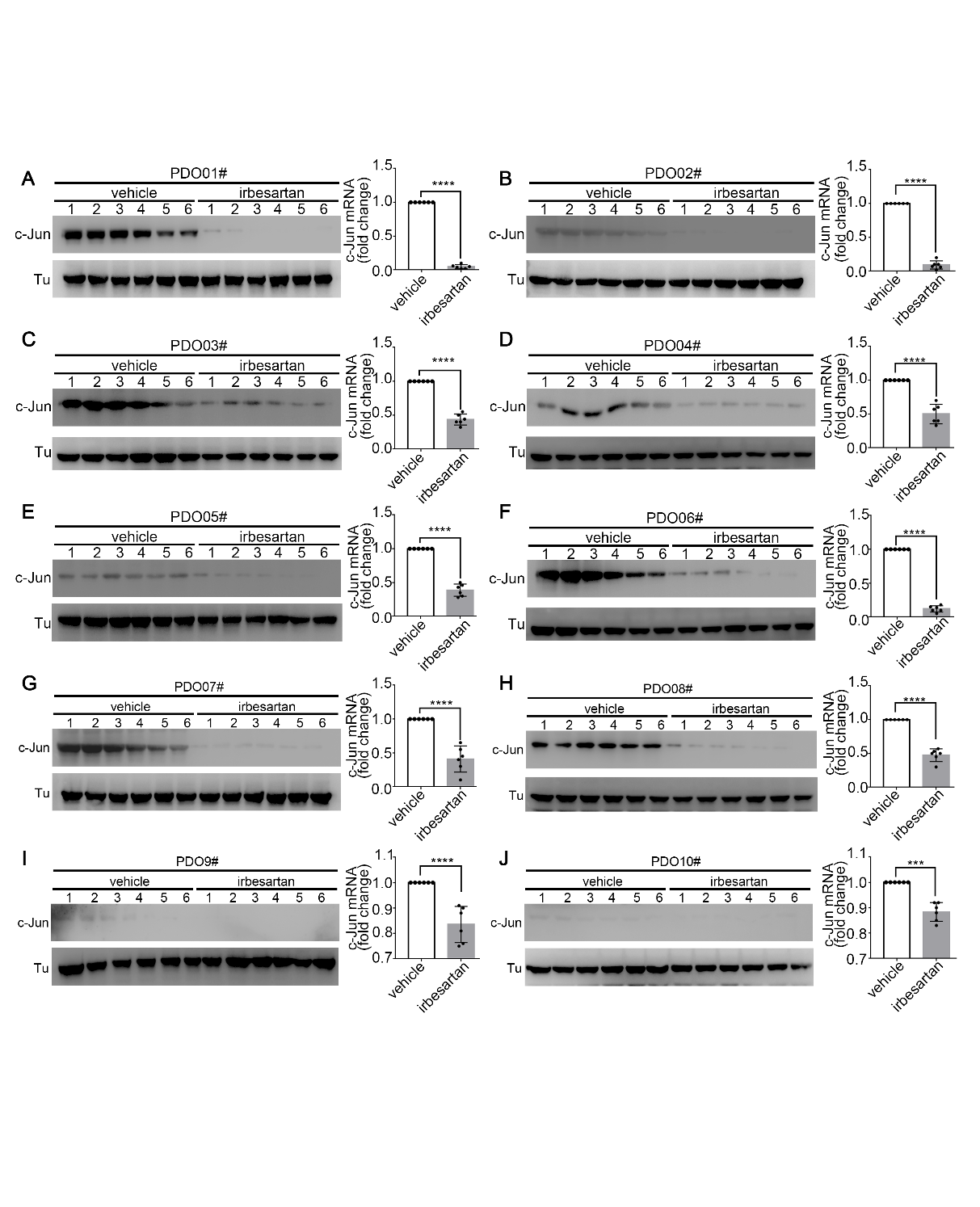
**

**Figure S7: The effects of irbesartan on inhibiting c-Jun expression were validated in 10 patient-derived organoids (PDOs) tumors from Fig.2K and Fig.S3 via western blot and q-PCR.** Briefly, PDOs subcutaneous tumor were obtained and tissues proteins and mRNA were prepared for subsequent western blot and q-PCR experiments. Tubulin was used as loading control for western blots and actin was used for internal control for q-PCR. Representative western blots images were shown in **Fig.S7A-J**, **left** and statistical analysis of q-PCR results were shown in **Fig.S7A-J**, **right**. All experiments were repeated three times independently. Un-paired Student’s t-test were used for in vivo experiments.

**
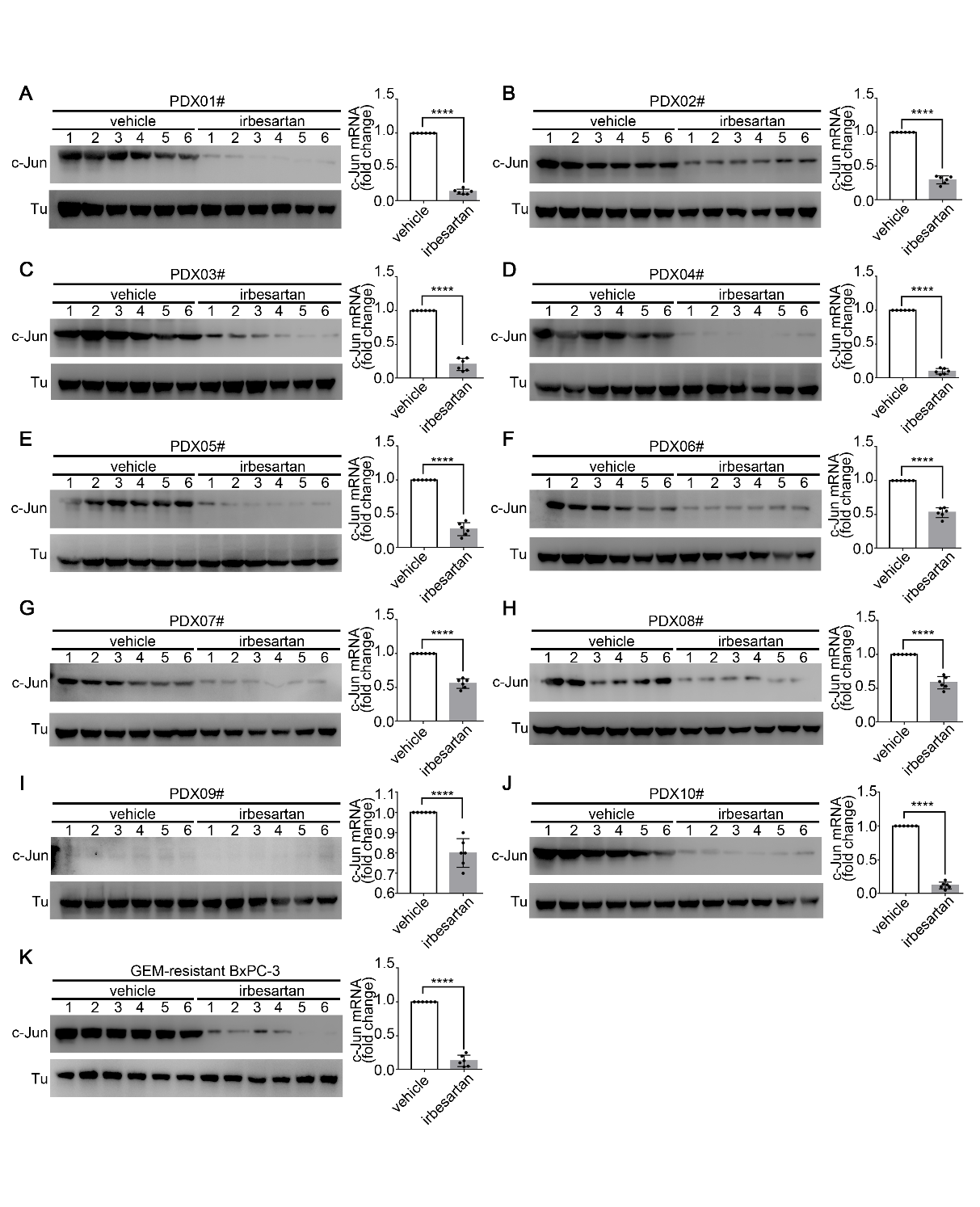
**

**Figure S8: The effects of irbesartan on inhibiting c-Jun expression were validated in 10 patient-derived xenografts (PDXs) subcutaneous tumors from Fig.S5, another GEM-resistant BxPC-3 orthotopic tumors from Fig. S6 via western blot and q-PCR.** Briefly, PDXs subcutaneous tumor and BxPC-3 orthotopic tumors were obtained and tissues proteins and mRNA were prepared for subsequent western blot and q-PCR experiments. Tubulin was used as loading control for western blots and actin was used for internal control for q-PCR. Representative western blots images were shown in **Fig.S8A-L**, **left** and statistical analysis of q-PCR results were shown in **Fig.S8A-L**, **right**. All experiments were repeated three times independently. Un-paired Student’s t-test were used for in vivo experiments.

**
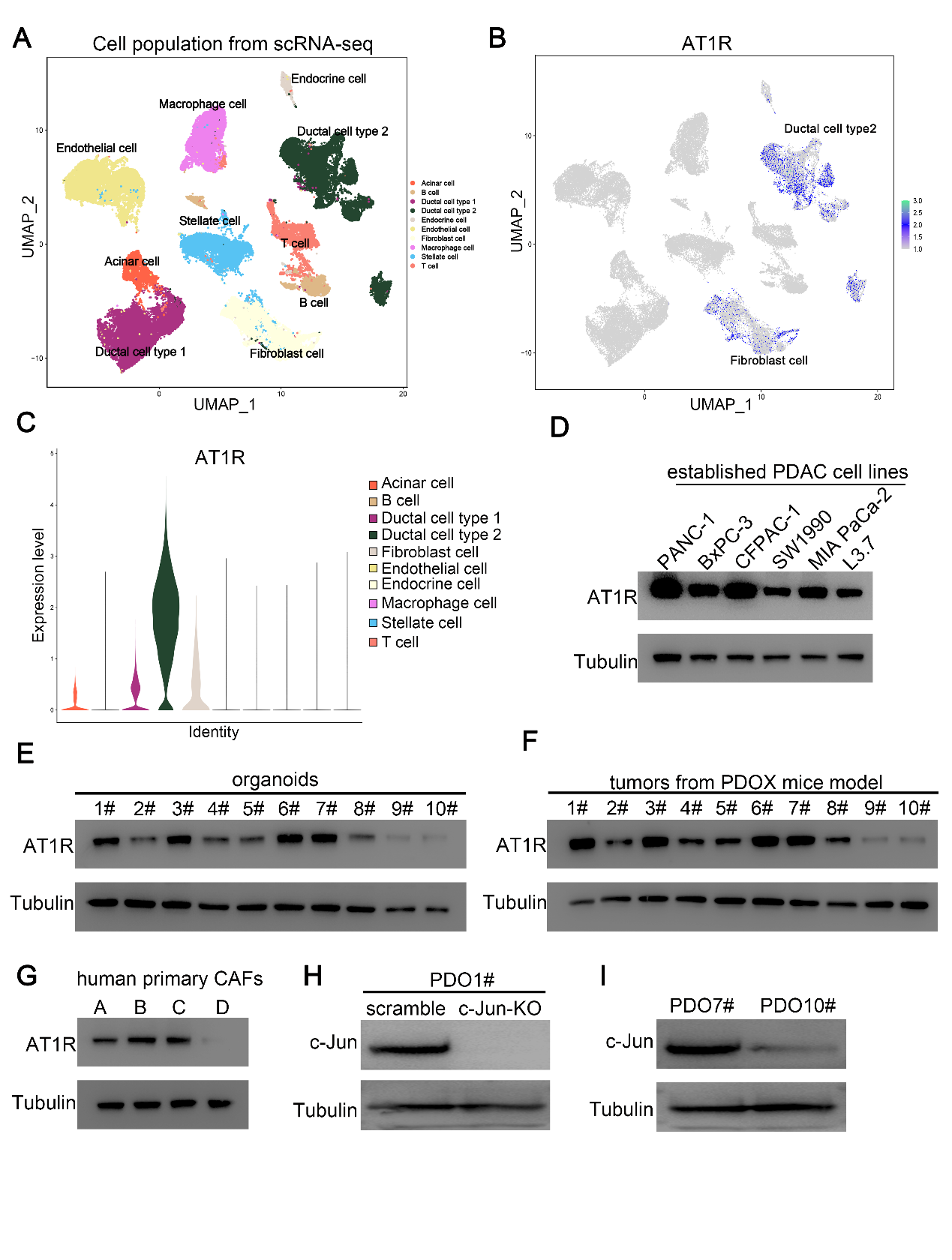
**

**Figure S9: Related to Fig.4.** (A) Unsupervised clustering of all viable cells from the human PDAC patients were shown as UMAP plots. (B) The expression of AT1R in all viable cells were analyzed and presented as UMAP plot. (C) The quantitative analysis for the AT1R mRNA expression level of all viable cell clusters were conducted and data were presented as a violin plot. (D) The expression level of AT1R in established human PDAC cell lines were evaluated by western blot. (E) The expression level of AT1R in human PDAC organoids were evaluated by western blot. (F) The expression level of AT1R in subcutaneous tumors from PDOX mice models were evaluated by western blot. (G) The expression level of AT1R in human primary CAFs were evaluated by western blot. (H-I) The expression of c-Jun in PDO1#-scramble/c-Jun-KO lines, PDO7# and PDO10# were validated by western blot. Tubulin was used as loading control

**
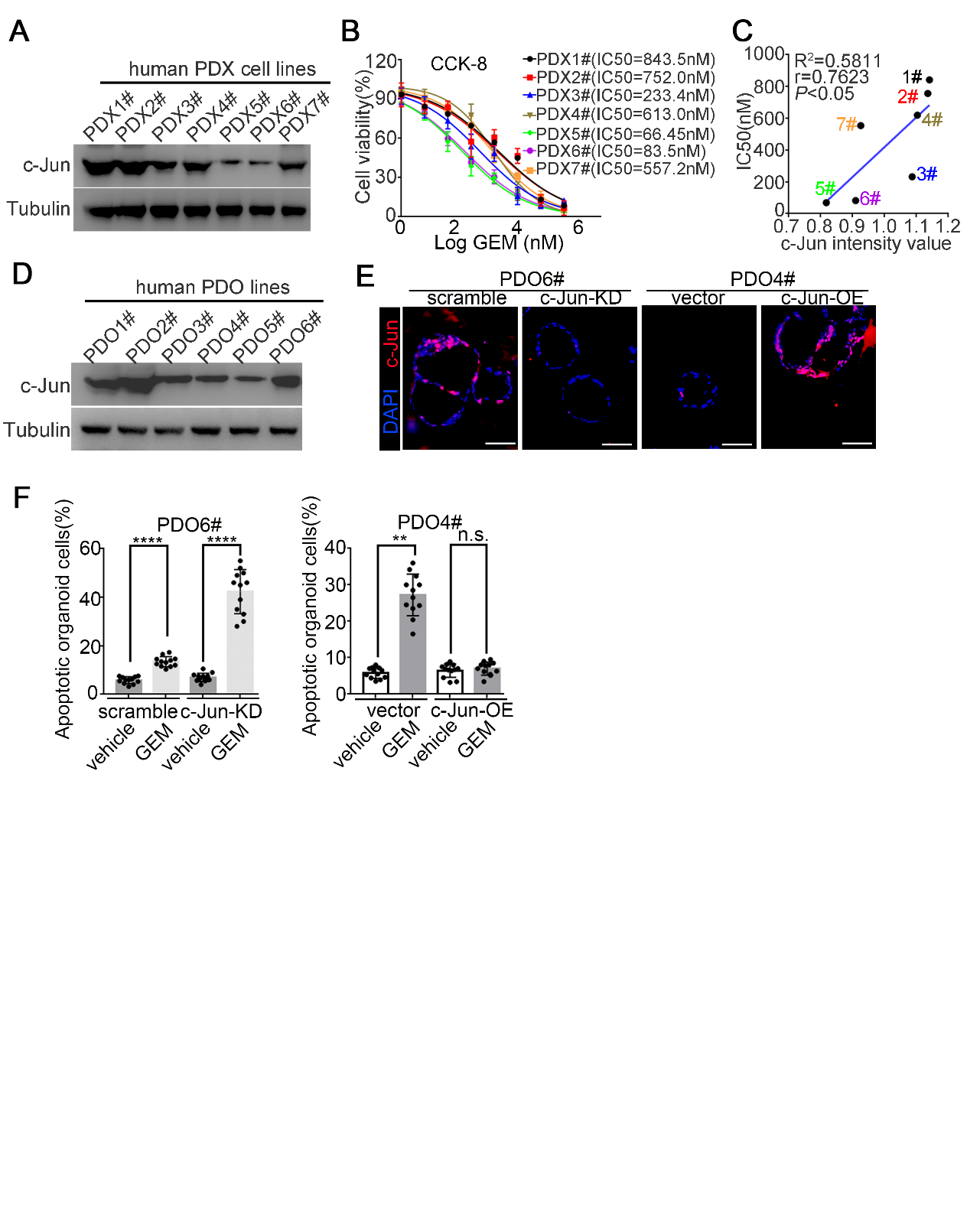
**

**Figure S10: Related to Figure 6.** (A) The basic expression of c-Jun in PDX1#-7# primary cell lines were determined by western blot. (B) The IC50 value of gemcitabine in PDX1#-7# primary cell lines were analysed by CCK-8 assays. (C) Spearman correlation analysis between c-Jun expression and the IC50 of PDXs. (D) The basic expression of c-Jun in PDO1#-6# was determined by western blot. (E) The expression of c-Jun in PDO6#-scramble/c-Jun-KD and PDO4#-vector/c-Jun-OE was validated by immunofluorescence staining. (F) The percentage of apoptotic organoid cells in PDO6#-scramble/c-Jun-KD (left) and PDO4#-vector/c-Jun-OE (right) treated with GEM in Figure 6D. All experiments were repeated three times independently. Paired Student’s t-test were used for in vitro experiments.

**
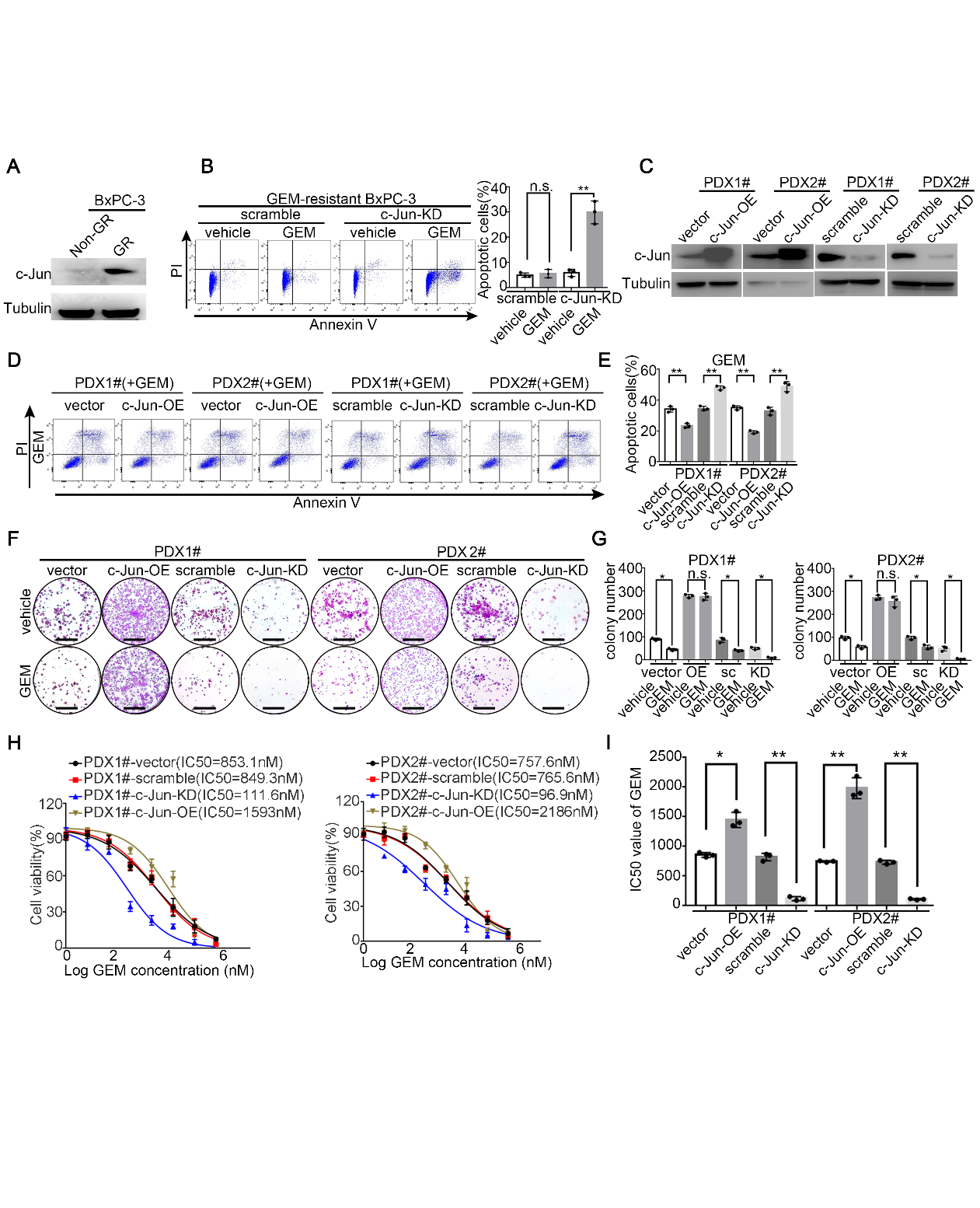
**

**Figure S11: Tumoral c-Jun induces chemotherapy resistance of GEM in 2D PDAC models in vitro.** (A) The c-Jun expression in non-GEM-resistant and GEM-resistant BxPC-3 were determined by western blot. Tubulin was used as loading control. (B) GEM-resistant BxPC-3-scramble/c-Jun-KD were treated with GEM (1μM, 72h) and then cellular apoptosis was detected by flow cytometry. Representative dot plots were shown (left) and statistical analysis were shown (right). (C) The c-Jun expression in established PDX1#-vector/c-Jun-OE, PDX1#-scramble/c-Jun-KD, PDX2#-vector/c-Jun-OE and PDX2#-scramble/c-Jun-KD cell lines were validated by western blot. Tubulin was used as loading control. (D-E) Indicated cell lines were treated with GEM (0.8μM, 72h) and then cellular apoptosis was detected by flow cytometry. Representative dot plots were shown (D) and statistical analysis were shown (E). (F-G) Indicated cell lines were used for colony formation assays in the presence of 0.4μM GEM for 2 weeks. Representative images were shown (F) and statistical analysis for colony number were shown (G). (H-I) The IC50 value of gemcitabine in indicated cell lines were determined by CCK-8 assays. Representative viability curve were shown (H) and statistical analysis for IC50 value were shown (I). All experiments were repeated three times independently. Paired Student’s t-test were used for in vitro experiments.

**
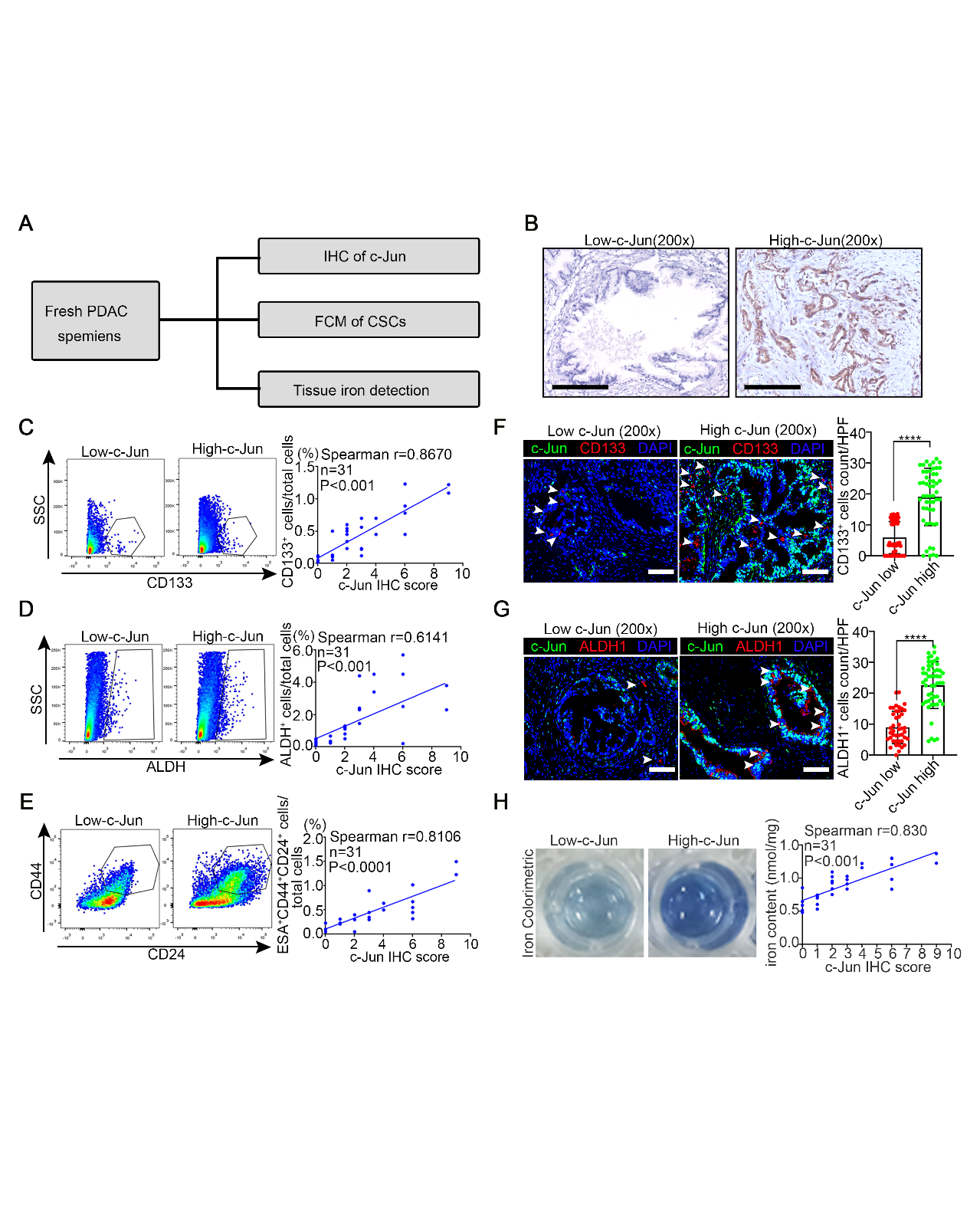
**

**Figure S12: Tumoral c-Jun is positively correlated with stemness profiles in PDAC.** (A) Schematic illustration for the experimental design. Fresh PDAC tissues were separated into three parts, one part was used to detect c-Jun expression by IHC staining; the second part was made into single cell suspension and prepared for CSCs detection by flow cytometry; the third part was prepared to quantify the cellular iron content by iron detection assay. (B-E) Single cell suspensions were prepared from 31 cases of fresh PDAC tissues and stained with ALDEFLUOR or specific antibodies against three CSC subsets (ALDH^+^ cells, CD133^+^ cells and CD44^+^ CD24^+^ cells). Representative IHC staining of c-Jun was shown (B). Scale Bar, 200μm. Representative dot plots of CD133^+^ cells (C, left), ALDH^+^ cells (D, left) and CD44^+^CD24^+^ cells (gated on ESA^+^ epithelial cells; E, left). Spearman correlation analysis between c-Jun IHC score and the proportions of CD133^+^ cells (C, right), ALDH^+^ cells (D, right) and ESA^+^CD44^+^CD24^+^ cells (E, right); n=31, *P*<0.001 and *P*<0.0001.

(F-G) Immunofluorescence staining of c-Jun expression and the accumulation of CD133^+^ cells (F) and ALDH1^+^ cells (G) in tumor tissues. The representative images from 95 pancreatic cancer cases were shown. The arrows indicated CD133^+^ cells and ALDH1^+^ cells. Representative images were shown (left) and statistical analysis were shown (right). Scale bars, 200μM. Non-paired Student’s t-test was used as statistical analysis; n=95, *P*<0.0001. (H) The association between c-Jun expression and iron content was determined in 31 fresh PDAC cases tissues. Representative images of iron assay were shown (left) and statistical analysis was shown (right).

**
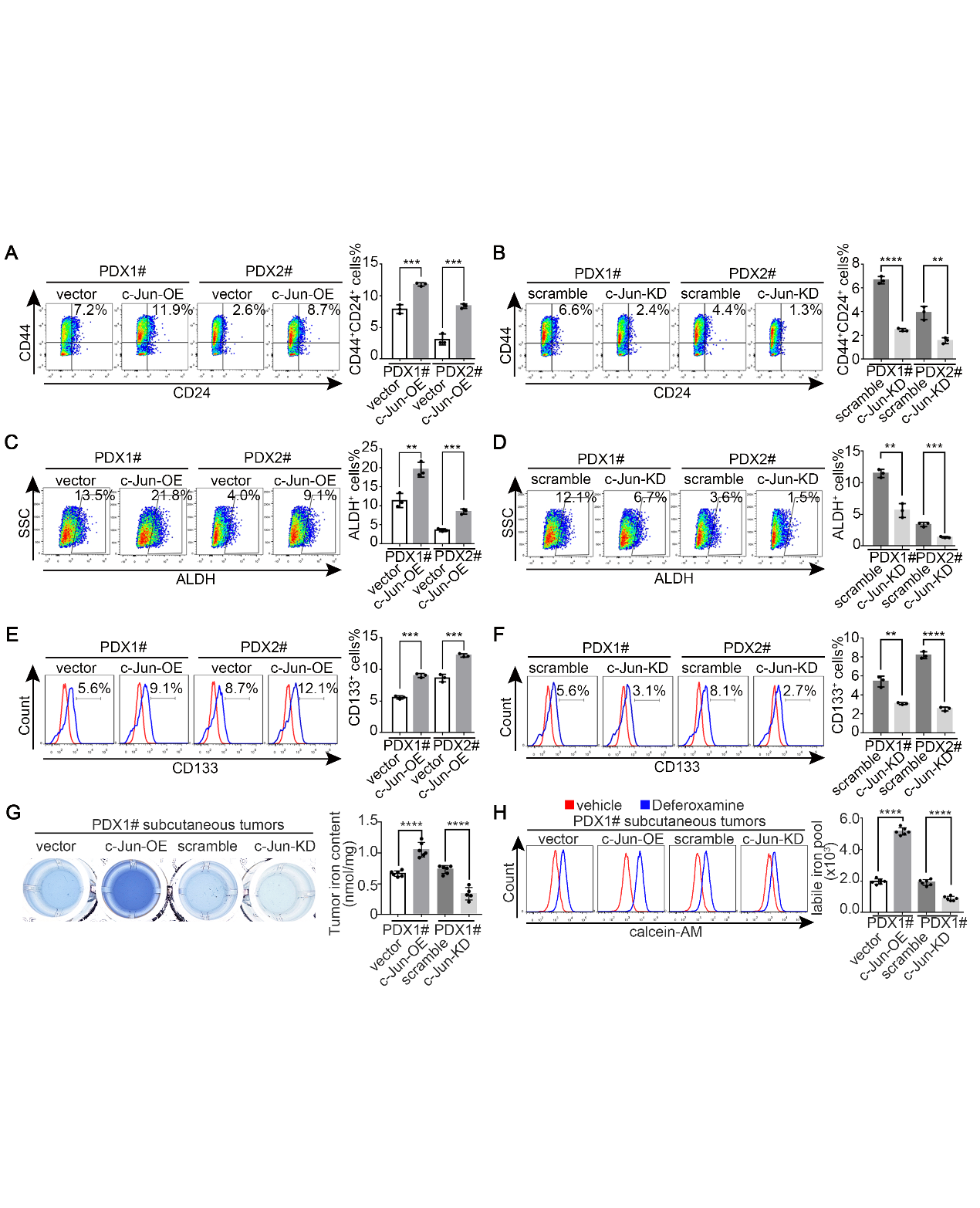
**

**Figure S13: Data related to Fig.7.** (A-B) The proportion of CD44^+^CD24^+^ cells in primary cancer cell lines PDX1#-vector/c-Jun-OE, PDX2#-vector/c-Jun-OE, PDX1#-scramble/c-Jun-KD and PDX2#-scramble/c-Jun-KD were analyzed using flow cytometry. Representative dot plots (A, left; B, left) and percentage of CD44^+^CD24^+^ cells (A, right; B, right) were shown. (C-D) The proportion of ALDH^+^ cells in indicated cells were analyzed using flow cytometry. Representative dot plots (C, left; D, left) and percentage of ALDH^+^ cells (C, right; D, right) were shown. (E-F) The proportion of CD133^+^ cells in indicated cells were analyzed using flow cytometry. Representative histograms (E, left; F, left) and percentage of CD133^+^ cells (E, right; F, right) were shown. (G-H)The iron content (G) and labile iron pool (H) in subcutaneous tumors from Fig.7G were determined. All experiments were repeated three times independently. Paired Student’s t-test were used for in vitro experiments.


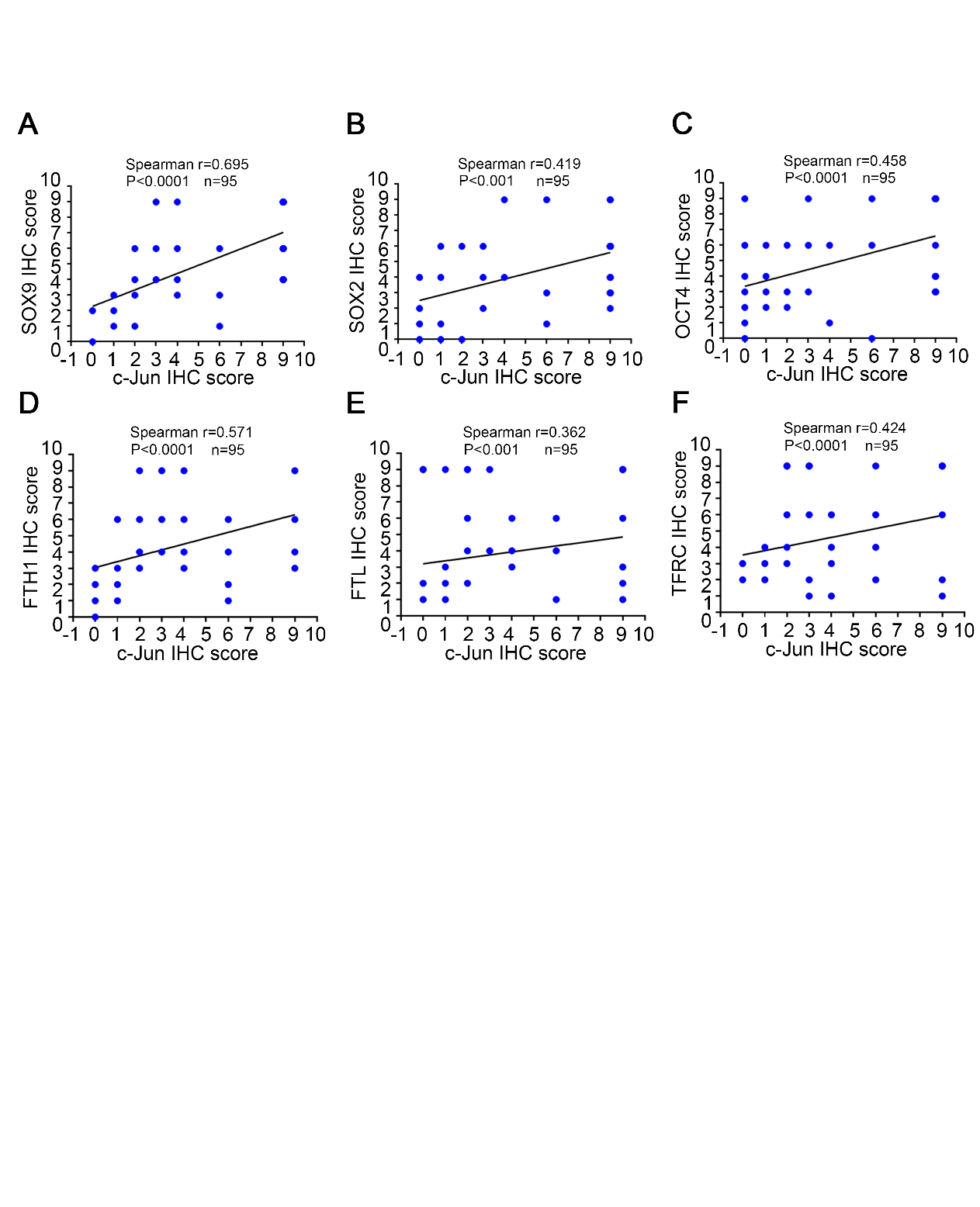


**Figure S14: Tumoral c-Jun positively regulates the expression of stemness genes (SOX9/SOX2/OCT4) and iron metabolism genes (FTH1/FTL/TFRC) in PDAC.** (A-C) 95 sets of consecutive PDAC tissues were stained with c-Jun, SOX9, SOX2 and OCT4. Spearman rank correlation analysis were used to evaluate the correlation between c-Jun and downstream stemness genes. (D-F) 95 sets of consecutive PDAC tissues were stained with c-Jun, FTH1, FTL and TFRC. Spearman rank correlation analysis were used to evaluate the correlation between c-Jun and downstream iron metabolism genes. All experiments were repeated three times independently.


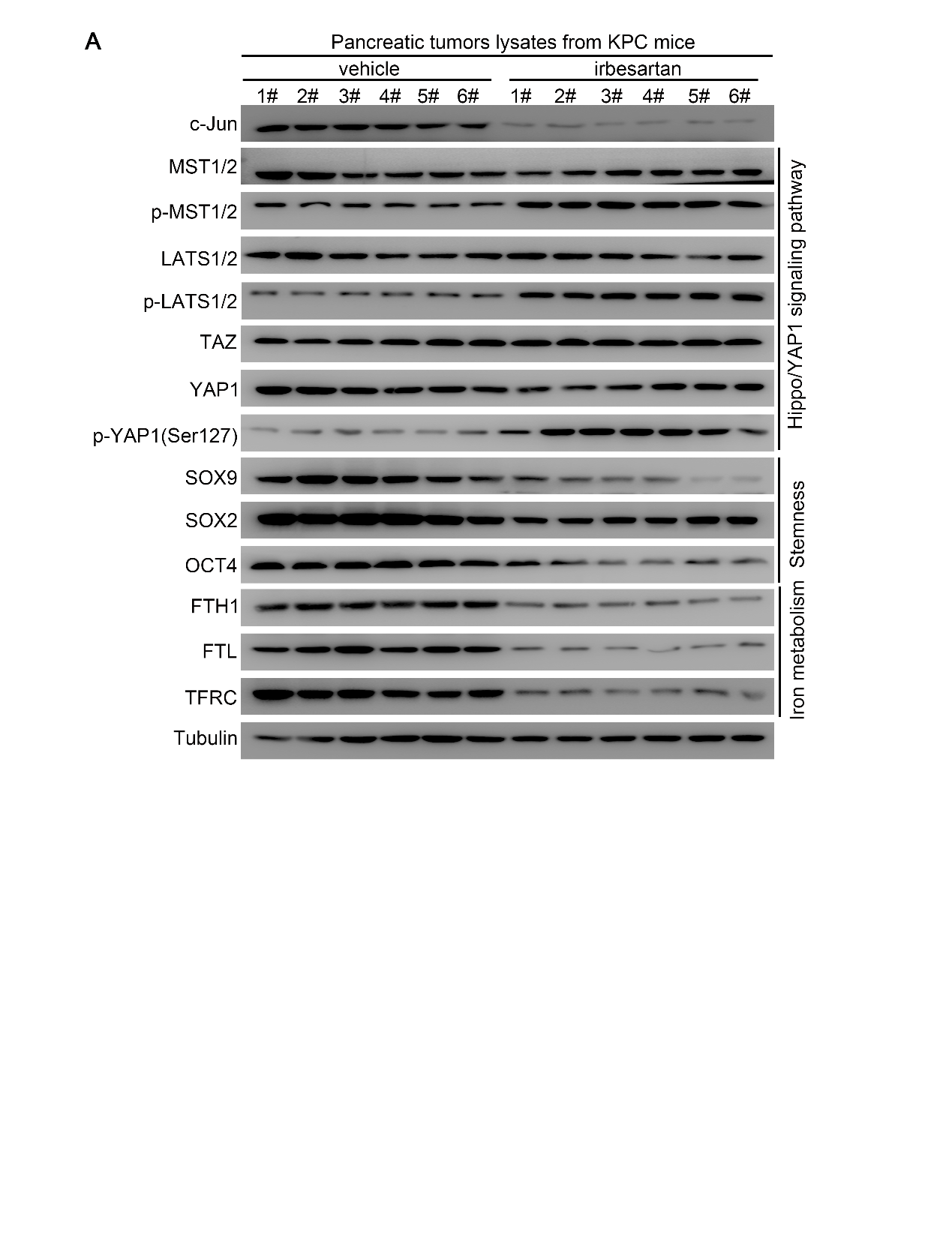


**Figure S15. Data related to Fig.10.** The effects of irbesartan on c-Jun expression, Hippo/YAP1 activity, and expression level of stemness/iron metabolism genes in pancreatic tumors of KPC mice model were evaluated by western blot. Tumor lysates were from Fig.3.

**Supplementary detailed methods**

**Patients and samples collection**

A total of 95 patients who had received radical surgery R0 resection with histological diagnosis of PDAC at the Tianjin Medical University Cancer Institute and Hospital, China from July 2011 to January 2015 were retrospectively collected in this study. Until the last follow-up date of October 23, 2019, the follow-up rate was 100%. Clinicopathological data of the 95 consecutive PDAC patients, including age, gender, histological grade, tumor size, TNM stage, regional lymph node status were obtained. None of the patients had received neoadjuvant chemotherapy or radiotherapy before tissue samples were collected. Systemic gemcitabine/nab-paclitaxel chemotherapy was given to all the patients after operation.

Another retrospective cohort including 58 patients who had received radical surgery R0 resection with histological diagnosis of PDAC at the Tianjin Medical University Cancer Institute and Hospital, China from April 2012 to December 2020 were collected in this study. Until the last follow-up date of December 30, 2021, the follow-up rate was 100%. Clinicopathological data of the 58 PDAC patients, including age, gender, histological grade, tumor size, TNM stage, regional lymph node status were obtained. None of the patients had received neoadjuvant chemotherapy or radiotherapy before tissue samples were collected. Systemic gemcitabine/nab-paclitaxel chemotherapy was given to all the patients after operation.

Another retrospective cohort including 104 patients who has been diagnosed with advanced PDAC based on their pancreatic needle biopsy pathology at the Tianjin Medical University Cancer Institute and Hospital, China from Jul 2015 to Jun 2020 were collected in this study. Until the last follow-up date of Jan 30, 2022, the follow-up rate was 100%. Clinicopathological data of the 104 PDAC patients, including age, gender, histological grade, tumor size, TNM stage, regional lymph node status were obtained. Systemic gemcitabine/paclitaxel chemotherapy was given to all the patients after diagnosis. GEM chemotherapy response of PDAC patients were recorded and divided into three categories: partial remission (PR), stable disease (SD) and progressed disease (PD).

Another retrospective cohort including 60 patients with hypertension who has been diagnosed with advanced PDAC based on their pancreatic needle biopsy pathology at the Tianjin Medical University Cancer Institute and Hospital, China from Jul 2015 to Jun 2020 were collected in this study. Until the last follow-up date of Jan 30, 2022, the follow-up rate was 100%. Clinicopathological data of the 60 PDAC patients, including irbesartan usage history, age, gender, histological grade, tumor size, TNM stage, regional lymph node status were obtained. Patients receiving other ARBs were excluded. Systemic gemcitabine/nab-paclitaxel chemotherapy was given to all the patients after diagnosis. GEM chemotherapy response of PDAC patients were recorded and divided into three categories: partial remission (PR), stable disease (SD) and progressed disease (PD).

Another retrospective cohort including 60 patients with hypertension who has been diagnosed with advanced PDAC based on their pancreatic needle biopsy pathology at the Tongliao City Hospital, China from Jan 2018 to Jan 2020 were collected in this study. Until the last follow-up date of Jun, 1, 2022, the follow-up rate was 100%. Clinicopathological data of the 60 PDAC patients, including irbesartan usage history, age, gender, histological grade, tumor size, TNM stage, regional lymph node status were obtained. Patients receiving other ARBs were excluded. Systemic gemcitabine/nab-paclitaxel chemotherapy was given to all the patients after diagnosis. GEM chemotherapy response of PDAC patients were recorded and divided into three categories: partial remission (PR), stable disease (SD) and progressed disease (PD).

From March 2020 to April 2021, 31 consecutive cases of fresh PDAC tissues were prospectively collected during operation. The PDAC tissue specimen collected was divided into three parts, one part was grinded and digested into single cell suspension for detection of CSCs (ESA^+^CD24^+^CD44^+^, ALDH^+^ and CD133^+^) by flow cytometry; the second part was fixed, embedded in paraffin and then prepared for IHC detection of c-Jun expression; the third part was used for detection of iron content of tumor tissues.

The usage of these specimens and the patients’ information were approved by the Ethics Committee of the Tianjin Medical University Cancer Institute and Hospital (Tianjin, China) and the Ethics Committee of the Tongliao City Hospital (Tongliao, China). All patients provided written consent for the use of their specimens and disease information for future investigations according to the ethics committee and in accordance with recognized ethical guidelines of Helsinki.

**Cell culture**

Human PDAC cell lines PANC-1, L3.7 and SW1990 were obtained from the Type Culture Collection Committee of the Chinese Academy of Sciences (Shanghai, China). The MiaPaca-2 cell line and the immortalized human pancreatic ductal cell line HPDE was purchased from ATCC in 2013. PANC-1, L3.7, SW1990 and MiaPaca-2 cell lines were cultured in DMEM or RPMI1640 basic medium supplemented with 10% Fetal Bovine Serum(FBS) and 1% penicillin-streptomycin solution at 37℃ in a humidified atmosphere of 95% air and 5% CO2.

**The establishment of primary PDAC cell lines**

Fresh human PDAC specimen tissues from PDX-bearing mice were acquired during surgery operation and immediately washed by PBS three times. Blood clots, dead tissues and other connective tissues were removed. PDAC tissues were cut into small pieces (1mm^3^) and then PDAC pieces were transferred into 15ml Conical centrifugal tube(Corning) resuspended with a mix of 5ml enzymes buffer containing 1 mg/ml collagenase(Sigma-Aldrich,C2799), 2.5 U/ml hyaluronidase(Sigma-Aldrich, H3506) and 0.1 mg/ml DNase(Sigma-Aldrich DN25) in 37℃ water bath for 4~6 h. The mix were then filtrated in a 30um strainer (MACS Smart Strainer) to obtain single cell suspension. The primary cancer cells were centrifuged, cell pellet was re-suspended with fresh medium and seeded in 6-well plates. Detailed information of PDX patients were listed in **supplementary table 2**. Low-passage (<10 passages) primary cancer cells were used for later experiments.

**The establishment of patient-derived organoid (PDO) and culture**

Organoids from human PDAC specimens were isolated and cultured as previously reported.^1, 2^ In brief, fresh PDAC specimens were cut into small pieces (<1mm^3^) and washed with cold PBS supplemented with 10% penicillin and streptomycin three times. And then the tissues were digested with digestion buffer with 1% foetal bovine serum,10% penicillin/streptomycin, 1.5 mg/mL collagenase type II, 500 U/mL type collagenase IV, 0.1 mg/mL dispase type II and 10 mM Y-27,632 (Selleck, Shanghai, China)) for 45 min at 37 °C with vigorous vibration. After digestion, the tumour pellets were washed with cold PBS three times and finally collected through centrifugation at 200 g for 5 min. Subsequently, tumour cells were embedded in Matrigel (BD) and plated in 96-well plates. After the polymerization of the Matrigel, these organoids were cultured in complete Advanced DMEM/F12 (Thermo Fisher Scientific) supplemented with Noggin (0.1mg/ml, Peprotech), R-spondin (1μg/ml, Nuvelo), epidermal growth factor (EGF, 50 ng/ml, Peprotech), Glutamax (invitrogen), HEPES (invitrogen), N2 (invitrogen), B27 (invitrogen), N-acetyl-L-cysteine (1mM,sigma), Gastrin (10 nM,sigma), Nicotinamide (10mM, sigma), A83-01 (0.5mM, Tocris Bioscience) and fiboblast growth factor 10( FGF10, 100ng/ml, Preprotech).

The organoids medium was changed approximately every 3 days, and organoids were passaged approximately every 7 days according to their growth conditions. The detailed information of patients were listed in **supplementary table 2**. To determine the genomic background, the single organoids were picked and expanded to obtain clonal organoid lines which were characterized by western blotting and prepared for next generation sequence (NGS).

**Establishment of gemcitabine-resistant pancreatic cancer cell lines**

Gemcitabine-resistant PDAC cell lines were established according to the methods reported previously. Briefly, the process of establishment includes two steps. In the adaptation stage, BxPC-3 cells were treated with gemcitabine for 72 hours by a gradual increase in drug concentration from 20 to 500 nM (20nM, 50nM, 100nM, 250nM and 500 nM). After each dose-induced step, we discarded apoptotic cells and amplified survival cells in gemcitabine-free culture medium; this step was repeated three times. Cells were then exposed to the next increment of gemcitabine over 6 months. In the consolidation stage, selected BxPC-3 cancer cells were treated with 2000nM gemcitabine until they grew normally in conditioned medium.

**Viability of 2D monolayer cancer cells measured by CCK8 assay**

To evaluate the cellular viability of PDAC cell lines, cell counting kit-8 (CCK-8, Bimake) was carried out. Cancer cell lines were seeded in clear, flat-bottom 96-well plates (Corning) at a density of 1000 cells per well. The following day, cancer cells were treated with vehicle, gemcitabine alone, irbesartan alone and gemcitabine plus irbesartan (6 duplications for each group) for 24h, 48h and 72h. And then, culture medium was removed and the cells were incubated with 100μl DMEM medium containing 10% CCK8 (Bimake, B34302) for 3 hours at 37℃ in an incubator. The absorbance were read at 595nm and the cellular viability curve was plotted.

**Viability of 3D organoid measured by CellTiterGlo assay**

To evaluate the cellular viability of organoids, CellTiterGlo 3D assay (Permoga, G9618) were performed. The medium of organoids was discarded and 50 μL of prewarmed detection reagent was added according to the procedure. The cells were then incubated for 10 minutes at room temperature and luminescence was measured at 560 nm by the BioTek plate reader.

**FDA-approved compound library**

An FDA-approved compound library containing in total 1304 FDA-approved drugs (L4200, TOPSCIENCE, 100μl 10mM DMSO stock) was acquired from Perkin-Elmer G3 high-throughput drug screening platform in Basic Medical Research Center of Tianjin Medical University. Hit molecules that were selected after screening the FDA-approved compound library as described below were re-validated through Topscience chemicals and provided as 10mM stock solutions in DMSO. The detailed information of drug library was listed in **supplementary table 1**.

**Fully automated FDA-approved compound library screening and analysis of drug screening results**

We performed our high-throughput screening on 2D/3D human PDAC models by Perkin Elmer G3 fully automatic integrated system in Basic Medical Research Center of Tianjin Medical University. Firstly, we chose organoid 01#, organoid 02# and organoid 07# for our further drug screening. Brifely, Organoids cultured in Matrigel domes were collected and dissociated to single cell suspension embedded in Matrigel (BD) at a density of 5x10^5^ cells per ml. Then, Organoids suspension were seeded in 384-well plates (Perkin Elmer, CellCarrier-384 plates, 6007550) at a 10μl dome per well by an automatic liquid handler integrated into the system. After domes seeding, the plates were incubated in an incubator at 37℃ for 30 minutes. Subsequently, complete organoid medium was added into the 384-well plates at a volume of 20μl per well by automatic liquid handler integrated into the system. The second day, gemcitabine as basic chemotherapeutic regimen were firstly added into the 384-well plates at a final concentration of 0.5μM and then compounds of the FDA-approved drug library (L4200, TOPSCIENCE) as chemotherapy adjuvants were added into the 384-well plates at a final concentration of 1μM. For each drug screening, we set four groups: DMSO group, GEM group, drug candidate group and combination group. Gemcitabine plus erlotinib as considered as positive control and gemcitabine plus solvent control (DMSO) was used as negative control. 3D PDAC organoids treated with chemotherapy regimens were continued to be cultured for 72 hours. Cellular viability of 3D were then evaluated by CelltiterGlo-3D assay and cellular inhibitory rate of single GEM (GEM**_i_**), single drug of library (X**_i_**) and combined regimen ([GEM+X]**_i_** were calculated. To confirm the killing effects of combination therapy and synergetic effects of GEM and candidate drugs, we defined the screening criteria of hits: [GEM+X]**_i_**>60% and [GEM+X]**_i_**> GEM**_i_**+ X**_i_**. Based on this screening criteria, we screened several potential candidates.

**Plasmid construction and lentiviral transduction**

Human c-Jun cDNA ([NM_002228.4](https://www.ncbi.nlm.nih.gov/nuccore/NM_002228.4)) was cloned into a pCDH-plasmid expression vector (pCDH-c-Jun) and pCDH-vector was used as control. For mouse c-Jun stable expression cell lines, mouse c-Jun cDNA (NM_010591.2) was cloned into a pLV-plasmid expression vector (pLV-c-Jun) and pLV-vector was used as control. For the stable c-Jun knockdown cell lines, shc-Jun, sequences were designed by Thermo Fisher shRNA designer (<http://rnaidesigner.thermofisher.com/rnaiexpress/design.do>) and BiOSETTIA shRNA designer (<https://biosettia.com/support/shrna-designer/>). Human c-Jun knock-out lentivirus by CRISPR/Cas9 was purchased from the Company GeneChem, China. Lentiviral infections were performed according to the standard procedures as previously reported. All sequences were listed in **supplementary table 10**.

**Histological evaluation by H&E staining**

The primary human PDAC tissues and PDOX tissues were fixed overnight in 4% paraformaldehyde at 4℃ and then were embedded in paraffin. Subsequently, sections (5 mm) were deparaffinized, hydrated, and stained with hematoxylin and eosin (H&E). The histological pathology was determined by two independent pathologists.

**Immunohistochemistry (IHC) staining**

IHC was performed to analyze c-Jun, FTH1, FTL, TFRC, YAP1, SOX9, SOX2 and OCT4 expression in pancreatic cancer tissues. All antibodies used in this project were listed in **supplementary table 11**. At first day, Paraffin-embedded specimen slides of PDAC were deparaffinized and then heated in a pressure plot for 2.5 min to retrieve the antigens. Subsequently, the slides were incubated with primary antibody overnight at 4℃. At second day, the sections were incubated with secondary antibody for 30 min at an incubator with the temperature of 37℃. The DAB substrate kit (ORIGENE, ZLI-9019) was used for the chromogenic reaction. The intensity of the staining was evaluated using the following criteria: 0, negative; 1, low; 2, medium; and 3, high. The extent of staining was scored as 0, 0% stained; 1, 1%–25% stained; 2, 26%–50% stained; and 3, 51%–100% stained. Five random ﬁelds (100×magniﬁcation) were evaluated under a light microscope. The ﬁnal scores were calculated by multiplying the scores of the intensity with those of the extent and dividing the samples into four grades: 0, negative (-); 1–2, low staining (+); 3–5, medium staining (++); and 6–9, high staining (+++). The following criteria were used to evaluate the expression level of c-Jun, SOX9, SOX2, OCT4, FTH1, FTL and TFRC in PDAC tumor tissues: high expression group, c-Jun, FTH1, FTL and TFRC were scored as ++/+++; low expression group, c-Jun, FTH1, FTL and TFRC were scored as -/+. IHC score was determined by two independent pathologists who were blinded to the patients’ clinical features and outcomes.

**Multiplex fluorescent IHC (mIHC) and Multispectral imaging**

The opal 7-color manual IHC kits (Perkin Elmer, 2395285) were used in the study. Anti-c-Jun (abcam, ab220113), anti-CD133 (Miltenyi Biotec, 130-118-143) and anti-ALDH (BD Biosciences, 611194) antibodies were used. In brief, 5μm PDAC slides were deparafﬁnized and rehydrated through a graded series of ethanol solutions:(100% 1×10min; 95% 1×10min; and rinse in 70%) prior to antigen retrieval in heated Citric Acid Buffer (pH 6.0) in microwave treatment for 15min (EZ Retriever microwave). Each slide was put through two sequential rounds of staining, each including a protein block with blocking buffer followed by primary antibody and corresponding secondary HRP-conjugated polymer. Each HRP-conjugated polymer mediated the covalent binding of a different ﬂuorophore for signal ampliﬁcation. This reaction was followed by additional antigen retrieval in heated Citric Acid Buffer (pH 6.0) for 15min to remove bound antibodies before the next step in the sequence. After two sequential reactions, slides were counterstained with DAPI for 5 mins and mounted with ﬂuorescence mounting medium (Thermofisher). CD133 or ALDH1 labeled by Opal 620, c-Jun labeled by Opal 690. Stained slides were scanned over the whole slide using the TissueGostics system. A slide reviewer were used to select the regions of interest were systematically gridded and selected to capture tissue heterogeneity in an unbiased manner. The selected images were then captured with a ×20 lens and unstained tissues were used to extract the tissue autofluorescence. Images were analyzed using TissueGostcs analysis software 2.4.

**Immunofluorescence staining of organoids**

Organoids were removed from Matrigel using Cell Recovery Solution (Corning) and then embedded in OCT and slices of 5 μm thickness were prepared. Organoid sections were fixed in 4% paraformaldehyde for 15 minutes at room temperature and then permeabilized with 0.1% Triton X-100 in phosphate-buffered saline for 10 minutes at room temperature, and blocked for 1 hour with 3% bovine serum albumin in phosphate-buffered saline. The cells were then stained with an anti-Ki67 antibody (ab1667, abcam, 1:100 dilution) or anti-c-Jun antibody (ab32137, abcam, 1:100) overnight at 4°C. Then, the cells were incubated with AF488-labelled secondary antibodies (A32723, Invitrogen) at room temperature for 1 h. Cells were mounted with 4, 6-diamidino-2-phenylindole (DAPI) solution (0100-20, Southern Biotech). Images were captured with a confocal fluorescence microscope (Zeiss).

**TUNEL staining assay**

Formalin-fixed tumor tissues were embedded in paraffin and sectioned at a thickness of 5μm. TUNEL assay was used to detect apoptosis in the tumor tissues according to the manufacturer’s protocol (Roche, 11684795910). Tissue sections were analyzed to detect the localized green fluorescence of apoptotic cells. DAPI was used to visualize the cell nuclei. Images were captured using the Zessi fluorescence microscope (400×).

**Apoptosis assay of organoids**

The apoptosis assay of organoids was performed as previously reported.^3^ The organoids cultured in 50 μl Matrigel were pre-treated with gemcitabine, irbesartan and vehicle control and then organoid apoptosis were detected using a green-fluorescent capase3/7 probe reagent (R3711, Invitrogen). Hoechst (135102, Invitrogen) was added to organoid culture system to visualize cells undergoing apoptosis. The apoptotic organoids were constantly monitored by Operetta CLS high content analysis system (Perkin Elmer) for 72 hours and quantified using Software Harmony 4.5.

**Drug synergy analysis**

To analyze the synergistic effects of the combination therapy between gemcitabine and irbesartan using the former organoid apoptosis assay. Synergy scores were calculated by Synergyfinder ver2 (<https://synergyfinder.fimm.fi>).^4^ The interpretation of the final synergy scores as follows: less than -10: the interaction between two drugs is likely to be antagonistic; from -10 to 10: the interaction between two drugs is likely to be additive; larger than 10: the interaction between two drugs is likely to be synergistic

**Western blotting**

Whole-cell extracts were prepared by lysing the cells with SDS protein lysis buffer supplemented with proteinase inhibitor cocktail (bimake, B14001). Protein lysate was separated by SDS-PAGE, and then, the targeted proteins were detected by Western blotting with the primary antibodies to c-Jun, MST1, p-MST1, LATS1, p-LATS1, YAP1, p-YAP1 (Ser127), TAZ, Sox9, Sox2, OCT4, FTH1, FTL and TFRC. β-tubulin was used as a loading control. Secondary antibodies: Goat anti-rabbit or mouse antibody at 1:5000(Abmart). The detailed information of antibodies used in western lot were listed in **Supplementary Table 11**.

**Reverse transcription PCR (RT-PCR)**

The total RNA was extracted from PDAC cell lines using TRizol reagent (Invitrogen). And then, the mRNA were used for first-strand cDNA synthesis with the Reverse Transcriptional PCR system (bimake) according to the manufacturer’s instructions. RT-PCR was performed to detect the expression of cDNA level of targeted genes. Each RT-PCR experiment was repeated independently at least three times. Actin was used as loading control. The detailed information of PCR primers sequences used were listed in **Supplementary Table 12**.

**Colony formation assay**

Cells seeded in six-well plates (500 cells/well) were treated with gemcitabine (0.4μM) and continuously cultured for 14 days without disturbance. Culture medium was replaced every 5 days. Colonies were fixed in 1% formaldehyde for 30 min, stained with 0.1% crystal violet for 30 min, and photographed.

**5-ethynyl-2’-deoxyuridine (EdU) staining**

The proliferative ability of cancer cells were determined by Edu Staining (C6015, US, China) according to the instructions. Brifely, cancer cells were seeded into a 96-well plate at a density of 4 × 10^4^ cells/well and cultured overnight at 37 °C. The next day, cells were fixed with 1% formaldehyde for 10 min, followed by incubated with 50 µM EdU labeling medium at 37 °C for 2 h. The cell nucleus was stained with Hochest33342 for 30 min. Finally, the Edu-positive cells (red) and DAPI-positive cells (blue) were observed under a fluorescent microscope (Leica).

**ALDEFLUOR assay**

The ALDEFLUOR™ kit (STEMCELL Technologies, 01700) was used to analyze the subpopulation with the high ALDH enzymatic activity which has been considered as the marker of pancreatic CSCs. The assay follows the protocols suggested by the manufacturer. 1 × 10^6^ cells were suspended in ALDEFLUOR assay buffer containing ALDH substrate (BAAA) and incubated in a cell incubator for 60 min at 37°C. For the negative control, each sample aliquot was treated with diethylamino benzaldehyde (DEAB), a specific ALDH inhibitor. The cell flow cytometric sorting gates were established using DEAB treated cells as negative controls.

**Detection of cancer stem cells (CSCs) by flow cytometry**

To investigate the relationship between the expression of tumoral c-Jun and percentages of pancreatic CSCs subsets in PDAC tissues, fresh PDAC specimens were collected and divided into three parts, one part was immediately fixed in formalin buffer, embedded in paraffin and prepared for detection of c-Jun via IHC staining; the second part was immediately digested into the single cell suspension with 1 mg/ml collagenase (Sigma-Aldrich, C2799), 2.5 U/ml hyaluronidase (Sigma-Aldrich, H3506) and 0.1 mg/ml DNase (Sigma-Aldrich DN25). To evaluate the percentages of CSCs subsets in PDAC tumor tissues, single cell suspension was divided into three parts, three different combination of CSCs markers were used, including ESA^+^CD44^+^CD24^+^ CSCs, CD133^+^ CSCs and ALDH^+^ CSCs. Related antibodies were used according the instructions in **Supplementary Table 11**.

The percentages of CSCs subsets in human pancreatic cancer cell lines were also detected by flow cytometry. Three different combination of CSCs markers was used, including CD44^+^CD24^+^ CSCs, CD133^+^ CSCs and ALDH^+^ CSCs. Isotype controls were used as negative controls. The data were analyzed using soft Flow Jo 10.0

**Measurement of cellular apoptosis by flow cytometry**

To investigate the gemcitabine responses in PDAC cell lines, annexin V and propidium iodide (PI) staining were carried out to evaluate cellular apoptosis levels. Indicated cell lines were counted, plated in six-well plates and grown overnight. In the following day, the adherent cell lines were treated with 400nM gemcitabine and PBS was used as control. After 72 hours, the medium containing detached cells were collected and the remaining adherent cells were harvested by EDTA-free trypsinization. The cell pellets were washed with cold PBS once and then suspended with 500ul 1×binding buffer, and then the cellular suspension were stained with 5μl Annexin V-APC and 5μl PI (Biolegend) in room temperature for 30 minutes. Cellular apoptosis were detected by flow cytometry. Annexin V^+^ cells were defined as apoptotic cells. Annexin V^+^/PI^+^ cell population was considered as late-apoptotic cells and Annexin V^+^/PI^-^ population was considered as early-apoptotic cells. The data were analyzed using soft Flow Jo 10.0.

**Sphere formation assay**

PDACs (5000 cells/ml) were cultured in ultra-low adhesion plates (Corning) in serum-free DMEM/F12 medium (GIBCO), which contains B27 (1:50, Invitrogen), 20ng/ml EGF (Proteintech), 10ng/ml FGF2 (Proteintech), 0.4% Bovine Serum Albumin (Sigma) and 5μg/ml insulin (Sigma). After 2 weeks, tumor spheres with diameter>75μm were counted. To evaluate the effects of irbesartan on sphere formation capacity, cancer cells were pre-treated with irbesartan (1μM) for 48 hours; and then, pre-treated cancer cells (5000 cells/ml) were collected and seeded in ultra-low adhesion plates (Corning) in stem cell medium. After 2 weeks, tumor spheres with diameter>75μm were counted.

**In *vitro* limiting dilution tumorsphere formation assay**

To determine the capacity of self-renewal of PDAC cancer cell lines, in vitro limiting dilution tumorsphere formation assay was performed. In brief, different numbers of cells per well (range of 500-25 cells per well) were seeded in 96-well plates containing 100 µl stem cell medium. After 14 d, the tumor spheres were measured and analyzed by Extreme Limiting Dilution analysis software (<http://bioinf.wehi.edu.au/software/elda>).

**Cancer stem cells (CSCs) sorting by FACS**

CD133^+^ CSCs and CD133^-^ CSCs were sorted from primary pancreatic cancer cell lines using CD133 antibody-conjugated magnetic microbeads (Miltenyi Biotec, 130-097-049) under sterile conditions according to the instructions of the manufacturer. Our analysis revealed a purity>95% of CD133^+^ CSCs and CD133^-^ non-CSCs. Cell viability was checked by Trypan blue dye exclusion. Firstly, the sorted CD133^+^ and CD133^-^ cells were subcutaneously implanted into the NSG mice and the tumor growth was monitored. Secondly, the sorted CD133^+^ CSCs and CD133^-^ non-CSCs were used for detection of c-Jun and stemness markers (Sox9, Sox2, Nanog and Oct4) by western blot.

**Determination of intracellular iron content**

Intracellular ferrous iron level (Fe2+) were evaluated by using the iron assay kit (ab83366, Abcam). Briefly, tumor tissues or cancer cells were collected, washed with cold PBS, and homogenized in iron assay buffer. And then the supernatant were added with iron reducer and subsequently for 30 minutes at 37 ℃ in an incubator. Finally, iron probe was added, mixed, and incubated for another 1 hour, and the value of OD was immediately measured on a colorimetric micro-plate reader at the absorbance of 593nm. Cellular iron content was analyzed according to the standard curve.

**In vitro transferrin uptake imaging assay**

PDAC-vector/scramble cells and PDAC-c-Jun/shc-Jun cells were pre-labeled with Cell Tracker Blue CMHC dye (Invitrogen, cat: C2111) and Cell Tracker Green CMFDA dye (Invitrogen, cat: C7025), respectively; and then, cancer cells were washed by PBS three times. Subsequently, PDAC-vector cells were mixed with PDAC-c-Jun cells and PDAC-scramble cell were mixed with PDAC-c-Jun-KD cells, respectively. Next, the mixed cancer cells were counted and seeded in 6-well plate overnight. The following day, the culture medium was replaced with conditioned medium containing 1μg/ml red-conjugate transferrin. After 30 minutes, transferrin uptake images were captured on Laser Scanning Confocal Microscopy (Zeiss). Representative Field showing TF (red) in PDAC-vector/scramble (blue) and PDAC-c-Jun/c-Jun-KD (green) and the capacity of transferrin uptake were quantified by measuring the ratio of area of cellular TF and area of cellular surface.

**Detection of liable iron pool (LIP) by flow cytometry**

Cells were washed with cold PBS three times and incubated with 0.6μM calcein-AM for 10 minutes at 37℃ in an incubator. And then, the cells were washed with cold PBS three times and treated with or without Deferiprone. Next, stained cells were analyzed by flow cytometry at a 488 nm laser. Calcein was exited at 488 nm and fluorescence was measured at 530 nm. The mean fluorescence intensity (MFI) of calcein were calculated by Flowjo 10.0 and the relative LIP were calculated as the difference of MFI between cancer cells with Deferiprone treatment and cancer cells without Deferiprone treatment.

**Chromatin immunoprecipitation (Ch-IP) and luciferase analysis**

Ch-IP assays were performed using Ch-IP kit (Millipore) according to the manufacturer's instructions. To detect whether c-Jun directly bound to the promoter region of SOX9, SOX2, OCT4, FTH1, FTL and TFRC, PDAC cells were immunoprecipitated with anti-c-Jun antibody (abcam, ab32137). And then the immunoprecipitated products were detected by PCR. Detailed information of related Ch-IP primers were listed in **supplementary table 12**. To detect whether c-Jun directly induced the transcriptional activity of SOX9, SOX2, OCT4, FTH1, FTL and TFRC, luciferase analyses were performed using dual-luciferase reporter assay kit (Promega, E1910). In brief, cancer cells were transfected with pCDH-c-Jun plasmid or control vector (pCDH-vector), which were subsequently transfected with pGL3-*SOX9*-binding site-WT (wild type), pGL3-*SOX9*-binding site1-mutation, pGL3-*SOX9*-binding site2-mutation, pGL3-*SOX9*-binding site1+2-mutation; pGL3-*SOX2*-binding site-WT (wild type), pGL3-*SOX2*-binding site1-mutation, pGL3-*SOX2*-binding site2-mutation, pGL3-*SOX2*-binding site3-mutation, pGL3-*SOX2*-binding site1+2-mutation, pGL3-*SOX2*-binding site1+3-mutation, pGL3-*SOX2*-binding site2+3-mutation and pGL3-*SOX2*-binding site1+2+3-mutation; pGL3-*OCT4*-binding site-WT (wild type), pGL3-*OCT4*-binding site1-mutation; pGL3-*FTH1*-binding site-WT (wild type), pGL3-*FTH1*-binding site1-mutation; pGL3-*FTL*-binding site-WT (wild type), pGL3-*FTL*-binding site1-mutation; pGL3-*TFRC*-binding site-WT (wild type), pGL3-*TFRC*-binding site1-mutation, pGL3-*TFRC*-binding site2-mutation, pGL3-*TFRC*-binding site3-mutation, pGL3-*TFRC*-binding site1+2-mutation, pGL3-*TFRC*-binding site1+3-mutation, pGL3-*TFRC*-binding site2+3-mutation and pGL3-*TFRC*-binding site1+2+3-mutation,respectively. Forty-eight hours later, cells were subjected to dual luciferase analysis. The results of luciferase analysis were expressed as a fold induction relative to the cells transfected with the control vector after normalization to Renilla activity. Related sequences of the vectors for luciferase analysis were listed in **supplementary table 13**.

To detect whether TEAD directly bound to the promoter region of c-Jun, PDAC cells were immunoprecipitated with anti-TEAD1 antibody (CST: #12292). And then the immunoprecipitated products were detected by PCR. Detailed information of related Ch-IP primers were listed in **supplementary table 12**. To detect whether irbesartan suppressed the transcriptional activity of c-Jun regulated by TEAD, luciferase analyses were performed using dual-luciferase reporter assay kit (Promega, E1910). In brief, cancer cells were divided into vehicle group and irbesartan group (1μM) and then PDAC cells each group were transfected with pCDH-TEAD1 plasmid or control vector (pCDH-vector), which were subsequently transfected with pGL3-*TEAD1*-binding site-WT (wild type), pGL3-*TEAD1*-binding site1-mutation, pGL3-*TEAD1*-binding site2-mutation, pGL3-*TEAD1*-binding site1+2-mutation,respectively.Forty-eight hours later, cells were subjected to dual luciferase analysis. The results of luciferase analysis were expressed as a fold induction relative to the cells transfected with the control vector after normalization to Renilla activity. Related sequences of the vectors for luciferase analysis were listed in **supplementary table 13**.

**In *vivo* limiting dilution tumorsphere formation assay**

Female 4–6-week-old NOD/SCID mice were purchased from SPF Biotechnology Company (Beijing). All mice were maintained in specific pathogen–free conditions, and animal experiment procedures were approved by the Ethics Committee of Tianjin Medical University Cancer Institute and Hospital, in compliance with the principles and procedures of the NIH Guide for the Care and Use of Laboratory Animals.

To verify the effects of c-Jun on PDAC cellular stemness, mice were randomized into four groups: (A) PDX1#-vector group, (B) PDX1#-c-Jun-OE group, (C) PDX1#-scramble group, (D) PDX1#-c-Jun-KD group. In each group, cancer cells at a dilution range of 1×10^3^，1×10^5^ and 1×10^6^ were suspended in a 60μl mix of Matrigel plug (Corning,356234) and PBS at a 1:1 ratio and then subcutaneously injected into contralateral flanks of the mice. Implanted subcutaneous tumors were monitored and the volume of tumor were measured three times a week using a caliper. All the mice were euthanized at the end of two months. Subcutaneous tumors were harvested and tumor incidence were analyzed. Stem cell frequency were calculated on website <http://bioinf.wehi.edu.au/software/elda/>.

To validate whether irbesartan repressed cellular stemness in PDAC, mice were also randomized into groups: (A) PDO1#-vehicle group, (B) PDO1#-irbesartan group, (C) PDX1#-vehicle group, (D) PDX1#-irbesartan group. In each group, cancer cells at a dilution range of 1×10^3^，1×10^5^ and 1×10^6^ were suspended in a 60μl mix of Matrigel plug (Corning,356234) and PBS at a 1:1 ratio and then subcutaneously injected into contralateral flanks of the NOD/SCID mice. Five days later, mice were orally administrate with irbesartan (20mg/kg/day, twice a week) and vehicle. Implanted subcutaneous tumors were monitored and the volume of tumor were measured three times a week using a caliper. All the mice were euthanized at the end of three months. Subcutaneous tumors were harvested and tumor incidence were analyzed. Stem cell frequency were calculated on website <http://bioinf.wehi.edu.au/software/elda/>.

**In vivo evaluation of the therapeutic effects of irbesartan and GEM on human PDAC 2D/3D models**

To investigate the effects of irbesartan on enhancing capacity of GEM to kill cancer cells, an orthotopical mice model using 3D organoid were performed. In brief, female 4~6 NOD/SCID mice were randomized into four groups (PBS buffer was used as the vehicle for gemcitabine and 6 mice for each group): (A) PDO1#-vehicle, (B) PDO1#-gemcitabine, (C) PDO1#-irbesartan (D) PDO1#-gemcitabine plus irbesartan. A total of 1×10^6^ indicated PDO1# cancer cells were suspended in a 60μl mix of Matrigel and PBS at a 1:1 ratio and orthotopically injected into pancreas of each mouse to form tumors. Drug was administrated when the tumor volume reached 50mm^3^ monitored by MRI scanning and the first day of administration was defined as Day 0. Gemcitabine (purchased from MCE, HY-17026) was intraperitoneally administrated at a dose of 100mg/kg once a week, irbesartan (purchased from MCE, HY-B0202) was orally administrated at a dose of 20mg/kg twice a week. Pancreatic tumor growth was monitored once a week using MRI scans and tumor volumes were calculated by the following formula: Volume= 1/2 L1× (L2)^2^, where L1 is the long axis and L2 is the short axis. Mice body weight were monitored once a week. Mice were separated in 2 sets. For set 1, gemcitabine or irbesartan were administrated until death one week after tumor inoculation and survival curve was plotted. For set 2, mice were treated like in set 1 but killed after 6 weeks of treatment. For mice in set 2, pancreatic tumor tissues were harvested and tumor weights were measured. Tumor tissues were immediately ﬁxed in buffered formalin and embedded in paraffin and another part were kept at -80℃ for protein extraction and western blot analysis. Tissue slides (5μm) were prepared and Hematoxylin & Eosin (H&E) staining were performed for histopathological analysis according to instructions. IHC of Ki67 (abcam, ab16667) staining were performed to evaluate proliferation status of tumor tissues. TUNEL staining were performed to evaluate apoptotic cancer cells in tumor tissue.

To further validate the effects of irbesartan on enhancing capacity of GEM to kill cancer cells, a subcutaneous mice model using 3D organoid and PDXs were performed. In brief, female 4~6 NOD/SCID mice were randomized into four groups (PBS buffer was used as the vehicle for gemcitabine and 6 mice for each group): (A) vehicle, (B) gemcitabine, (C) irbesartan (D) gemcitabine plus irbesartan. A total of 1×10^6^ digested single cell suspensions from 3D PDO02/03/04/05/06/07/08/09/10 lines were suspended in a 60μl mix of Matrigel and PBS at a 1:1 ratio and subcutaneously injected into each mouse to form tumors. For PDX transplantation, small pieces (about 5mm) from PDX01/02/03/04/05/06/07/08/09/10 were subcutaneously transplanted into each mouse to form tumors. Drug was administrated when the subcutaneous tumor volumes reached 50mm^3^ monitored by MRI scanning. Gemcitabine (purchased from MCE, HY-17026) was intraperitoneally administrated at a dose of 100mg/kg twice a week, irbesartan (purchased from MCE, HY-B0202) was orally administrated at a dose of 20mg/kg twice a week. Pancreatic tumor growth was monitored once a week using a caliper and tumor volumes were calculated by the following formula: Volume= 1/2 L1× (L2)^2^, where L1 is the long axis and L2 is the short axis. Mice body weight were monitored once a week. 24 days later, the mice were sacrificed and subcutaneous tumor tissues were harvested and tumor weights were measured. Tumor tissues were immediately ﬁxed in buffered formalin and embedded in paraffin and another part were kept at -80℃ for protein extraction and western blot analysis. Tissue slides (5μm) were prepared and Hematoxylin & Eosin (H&E) staining were performed for histopathological analysis according to instructions. IHC of Ki67 (abcam, ab16667) staining were performed to evaluate proliferation status of tumor tissues. TUNEL staining were performed to evaluate apoptotic cancer cells in tumor tissue.

To investigate the effects of irbesartan on reversing gemcitabine resistance of PDAC, another orthotopical mice model using 2D GEM-resistant cancer cells were performed. In brief, female 4~6 BALB/C nude mice were randomized into four groups (PBS buffer was used as the vehicle for gemcitabine and 6 mice for each group): (A) vehicle (B) gemcitabine, (C) irbesartan (D) gemcitabine plus irbesartan. A total of 1×10^6^ indicated GEM-resistant BxPC-3 cancer cells were suspended in a 60μl mix of Matrigel and PBS at a 1:1 ratio and orthotopically injected into pancreas of each mouse to form tumors. Drug was administrated when the tumor volume reached 50mm^3^ monitored by MRI scanning. Gemcitabine (purchased from MCE, HY-17026) was intraperitoneally administrated at a dose of 100mg/kg once a week, irbesartan (purchased from MCE, HY-B0202) was orally administrated at a dose of 20mg/kg twice a week. Pancreatic tumor growth was monitored once a week using bioluminescent imaging (BLI). Mice body weight were monitored once a week. Mice were separated in 2 sets. For set 1, gemcitabine or irbesartan were administrated until death one week after tumor inoculation and survival curve was plotted. For set 2, mice were treated like in set 1 but killed after 4 weeks of treatment. For mice in set 2, pancreatic tumor tissues were harvested and tumor weights were measured. Tumor tissues were immediately ﬁxed in buffered formalin and embedded in paraffin and another part were kept at -80℃ for protein extraction and western blot analysis. Tissue slides (5μm) were prepared and Hematoxylin & Eosin (H&E) staining were performed for histopathological analysis according to instructions. IHC of Ki67 (abcam, ab16667) staining were performed to evaluate proliferation status of tumor tissues. TUNEL staining were performed to evaluate apoptotic cancer cells in tumor tissue.

**In vivo evaluation of the therapeutic effects of irbesartan in a c-Jun dependent manner on human PDAC models**

To investigate the effects of irbesartan on enhancing capacity of GEM to kill cancer cells in a c-Jun dependent manner, an orthotopical mice model using 3D organoids were performed. In brief, female 4~6 NOD/SCID mice were randomized into eight groups (PBS buffer was used as the vehicle for gemcitabine and 6 mice for each group): (A) PDO1#-scramble-vehicle, (B) PDO1#-scramble-gemcitabine, (C) PDO1#-scramble-irbesartan (D) PDO1#-scramble-gemcitabine plus irbesartan, (E) PDO1#-c-Jun-KO-vehicle, (F) PDO1#-c-Jun-KO-gemcitabine, (G) PDO1#-c-Jun-KO-irbesartan (H) PDO1#-c-Jun-KO-gemcitabine plus irbesartan. A total of 1×10^6^ indicated PDO1#-scramble/c-Jun-KO cancer cells were suspended in a 60μl mix of Matrigel and PBS at a 1:1 ratio and orthotopically injected into pancreas of each mouse to form tumors. Drug was administrated when the tumor volume reached 50mm^3^ monitored by MRI scanning. Gemcitabine (purchased from MCE, HY-17026) was intraperitoneally administrated at a dose of 100mg/kg twice a week, irbesartan (purchased from MCE, HY-B0202) was orally administrated at a dose of 20mg/kg twice a week. Pancreatic tumor growth was monitored once a week using MRI scans and tumor volumes were calculated by the following formula: Volume= 1/2 L1× (L2)^2^, where L1 is the long axis and L2 is the short axis. Mice body weight were monitored once a week. Mice were separated in 2 sets. For set 1, gemcitabine or irbesartan were administrated until death one week after tumor inoculation and survival curve was plotted. For set 2, mice were treated like in set 1 but killed after 6 weeks of treatment. For mice in set 2, pancreatic tumor tissues were harvested and tumor weights were measured. Tumor tissues were immediately ﬁxed in buffered formalin and embedded in paraffin and another part were kept at -80℃ for protein extraction and western blot analysis. Tissue slides (5μm) were prepared and Hematoxylin & Eosin (H&E) staining were performed for histopathological analysis according to instructions. TUNEL staining were performed to evaluate apoptotic cancer cells in tumor tissue.

**In vivo evaluation of the role or c-Jun in gemcitabine resistance on human PDAC 2D/3D models.**

To investigate the effects of c-Jun on gemcitabine resistance, an orthotopical mice model using 3D organoids were performed. In brief, female 4~6 NOD/SCID mice were randomized into four groups (PBS buffer was used as the vehicle for gemcitabine and 6 mice for each group): (A) PDO4#-vector (vehicle), (B) PDO4#-vector (gemcitabine), (C) PDO4#-c-Jun-OE (vehicle), (D) PDO4#-c-Jun-OE (gemcitabine). A total of 1×10^6^ indicated PDO4#-vector/c-Jun-OE cancer cells were suspended in a 60μl mix of Matrigel and PBS at a 1:1 ratio and orthotopically injected into pancreas of each mouse to form tumors. Drug was administrated when the tumor volume reached 50mm^3^ monitored by MRI scanning and the first day of administration was defined as Day0. Gemcitabine (purchased from MCE, HY-17026) was intraperitoneally administrated at a dose of 100mg/kg once a week. Pancreatic tumor growth was monitored once a week using MRI scans and tumor volumes were calculated by the following formula: Volume= 1/2 L1× (L2)^2^, where L1 is the long axis and L2 is the short axis. Mice body weight were monitored once a week. Mice were separated in 2 sets. For set 1, gemcitabine were administrated until death one week after tumor inoculation and survival curve was plotted. For set 2, mice were treated like in set 1 but killed after 6 weeks of treatment. For mice in set 2, pancreatic tumour tissues were harvested and tumor weights were measured. Tumor tissues were immediately ﬁxed in buffered formalin and embedded in paraffin and another part were kept at -80℃ for protein extraction and western blot analysis. Tissue slides (5μm) were prepared and Hematoxylin & Eosin (H&E) staining were performed for histopathological analysis according to instructions. IHC of Ki67 (abcam, ab16667) staining were performed to evaluate proliferation status of tumor tissues. TUNEL staining were performed to evaluate apoptotic cancer cells in tumor tissue.

To investigate the effects of c-Jun on gemcitabine resistance, a subcutaneous mice model using 2D PDX primary cell lines were carried out. In brief, female 4~6 BALB/C nude mice were randomized into eight groups (PBS buffer was used as the vehicle for gemcitabine and 6 mice for each group): (A) PDX1#-vector (vehicle), (B) PDX1#-vector (gemcitabine), (C) PDX1#-c-Jun-OE (vehicle), (D) PDX1#-c-Jun-OE (gemcitabine), (E) PDX1#-scramble (vehicle), (F) PDX1#-scramble (gemcitabine), (G) PDX1#-c-Jun-KD (vehicle), (H) PDX1#-c-Jun-KD (gemcitabine). A total of 1×10^6^ indicated PDX1#-vector/c-Jun-OE and PDX1#-scramble/c-Jun-KD cancer cells were subcutaneously injected into each mouse to form tumors. Drug was administrated when the tumor volume reached 50mm^3^ monitored by MRI scanning. Gemcitabine (purchased from MCE, HY-17026) was intraperitoneally administrated at a dose of 100 mg/kg once a week. Subcutaneous tumor growth was monitored a caliper and tumor volumes were calculated by the following formula: Volume= 1/2 L1× (L2)^2^, where L1 is the long axis and L2 is the short axis. Mice were sacrificed after 6 weeks of treatment. Subcutaneous tumor tissues were harvested and tumor weights and volumes were measured. Tumor tissues were immediately ﬁxed in buffered formalin and embedded in paraffin and another part were kept at -80℃ for protein extraction and western blot analysis. Tissue slides (5μm) were prepared and Hematoxylin & Eosin (H&E) staining were performed for histopathological analysis according to instructions. IHC of Ki67 (abcam, ab16667) staining were performed to evaluate proliferation status of tumor tissues. TUNEL staining were performed to evaluate apoptotic cancer cells in tumor tissue.

**KPC preclinical animal cohorts**

Pancreatic tumor volume of KPC mice were monitored twice a week by the MRI scanning.When pancreatic tumor of KPC mice initiated and reached 20~60mm3 , mice were then randomized into four groups: (A) vehicle (normal saline), (B) gemcitabine plus nab-paclitaxel (gemcitabine purchased from MCE, HY-17026, diluted in normal saline, 100mg/kg intraperitoneally once a week; nab-paclitaxel purchased from HengRui company, diluted in normal saline, 300mg/kg intravenously once a week), (C) irbesartan (purchased from MCE, HY-B0202; irbesartan were pre-dissolved in olive oil; 20mg/kg twice a week by oral gavage), (D) gemcitabine/nab-paclitaxel +irbesartan. Mice were separated in 2 sets. For set 1(8 mice per group), drug was administrated when the tumor reached 20~60mm3 until death. In set 2 (6 mice per group), mice were treated like in set 1 but killed after 8 weeks of treatment to compare tissues. The volume of pancreatic tumor was monitored twice a week by MRI scanning. For mice in set 2, pancreatic tumor tissues were harvested and weighed. Tumor tissues immediately fixed in buffered formalin and embedded in paraffin and another part were kept at -80℃ for protein extraction and western blot analysis. Tissue slides (5μm) were prepared and Hematoxylin & Eosin (H&E) staining were performed for histopathological analysis according to instructions. IHC of Ki67 (abcam, ab16667) staining were performed to evaluate proliferation status of tumor tissues. Tissues protein from vehicle group and rosiglitazone group were prepared for western blot to analyze the expression of c-Jun.

**RNA-sequencing**

Total RNA for samples was extracted using TRizol (Invitrogen) according to the manufacturer’s instructions. The quality and integrality of the total RNA were evaluated using the ratio of A260/280 and electrophoresed agarose gel. The mRNA samples were prepared for transcriptomic sequencing according to the following process. Firstly, mRNA was enriched and purified from total RNA using Oligo (dT) beads. The purified mRNA was cut into short fragments using a fragmentation buffer and used as templates to synthesize the first-strand and second-strand cDNA. After end repair and the addition of poly (A), the cDNA fragments were ligated with Illumina sequencing adapters. The ligation products were enriched by PCR amplification to construct the cDNA library template. Finally, the library was sequenced using Illumina HiSeqTM 2500 by Gene Denovo Biotechnology Co. (Guangzhou, China). The expression level for each gene was calculated by Fragments per Kilobase of transcript per Million mapped reads (FPKM). Differentially expressed genes (DEGs) between groups were identified by the edgeR package (http://www.rproject.org/) with a threshold of “False discovery rate (FDR) < 0.05 and the absolute value of the log2 fold change≥1.

**Statistical analysis**

Statistical analysis were performed with the IBM SPSS Statistics software version 21.0. Each experiment was conducted in triplicate. And the data values were presented as the mean ± SD, unless otherwise stated. The variance between different groups were statistically compared. Student’s t-test was used to compare the mean values. Median survival time was analysed using Kaplan–Meier curves and the log-rank test was used to analyse the differences in survival times among different groups. Spearman rank correlation was performed to evaluate the correlation between different parameters. The differential expression of targeted genes in paired tumour and non-tumour tissues was analysed by Wilcoxon signed rank tests. Repeated measure two-way ANOVA (tumour volume ×time) and post-hoc analysis were carried out for mice tumour volume growth analysis. The risk factors associated with the prognoses of these patients were evaluated with Cox’s proportional hazard regression model. *P<0.05, **P<0.01, ***P<0.001, ****P<0.001 and n.s. means non-significant.

**References**

1. Huch M, Bonfanti P, Boj SF, et al. Unlimited in vitro expansion of adult bi-potent pancreas progenitors through the Lgr5/R-spondin axis. Embo j 2013;32:2708-21.

2. Boj SF, Hwang CI, Baker LA, et al. Organoid models of human and mouse ductal pancreatic cancer. Cell 2015;160:324-38.

3. Koikawa K, Kibe S, Suizu F, et al. Targeting Pin1 renders pancreatic cancer eradicable by synergizing with immunochemotherapy. Cell 2021;184:4753-4771 e27.

4. Ianevski A, He L, Aittokallio T, et al. SynergyFinder: a web application for analyzing drug combination dose-response matrix data. Bioinformatics 2017;33:2413-2415.

**Supplementary Tables**

**Supplementary table1:** The detailed information of the FDA-approved drug library was shown in a supplementary EXCEL table named “**Table S1_Detailed information of drug library**”.

**Supplementary table2:** Detailed information of patients whose specimens were used for establishment of PDO models and in vivo PDX mouse models. The IC50 value of gemcitabine in PDO models and PDX-derived primary cell lines were determined by CellTiterGlo-3D assay and CCK-8 assays, respectively.

| Patient ID | Age | Gender | TNM stage | Pathological stage | PDAC differentiation grade | IC50 of GEM(nM) | RFS (months) |
| --- | --- | --- | --- | --- | --- | --- | --- |
| PDO01# | 66 | Male | T2N0M0 | ⅠB | moderated | 2483 | 6.5 |
| PDO02# | 54 | Male | T2N0M0 | ⅠB | moderated | 1638 | 7.3 |
| PDO03# | 59 | Male | T2N0M0 | ⅠB | low | 263 | 21.2 |
| PDO04# | 54 | Female | T2N0M0 | ⅠB | moderated | 214.7 | 22.3 |
| PDO05# | 65 | Male | T2N0M0 | ⅠB | moderated | 145.6 | 24.5 |
| PDO06# | 61 | Male | T2N1M0 | ⅡB | moderated | 627.2 | 15.2 |
| PDO07# | 69 | Male | T1cN0M0 | ⅠA | moderated | 2825 | 5.5 |
| PDO08# | 66 | Female | T1cN0M0 | ⅠA | moderated | 796.4 | 14.3 |
| PDO09# | 62 | Female | T2N0M0 | ⅠA | moderated | 248.8 | 20.5 |
| PDO10# | 68 | Male | T3N0M0 | ⅡA | moderated | 147.7 | 30.0 |
| PDX01# | 66 | Male | T3N0M0 | ⅡA | moderated | 841.2 | 14.6 |
| PDX02# | 59 | Male | T3N0M0 | ⅡA | low | 755.0 | 15.4 |
| PDX03# | 54 | Female | T2N1M0 | ⅡB | moderated | 231.7 | 20.3 |
| PDX04# | 46 | Male | T3N1M0 | ⅡB | low | 618.7 | 16.2 |
| PDX05# | 65 | Male | T2N0M0 | ⅠB | moderated | 68.35 | 32.3 |
| PDX06# | 55 | Male | T2N0M0 | ⅠB | moderated | 81.5 | 31.4 |
| PDX07# | 62 | Male | T2N0M0 | ⅠB | moderated | 553.4 | 16.3 |
| PDX08# | 65 | Male | T2N0M0 | ⅠB | moderated | 324.4 | 22.4 |
| PDX09# | 56 | Male | T2N0M0 | ⅠB | moderated | 367.4 | 19.4 |
| PDX10# | 55 | Female | T2N0M0 | ⅠB | low | 2586 | 6.3 |

**Supplementary table3:** The detailed information of cellular inhibitory rate of 1321 drugs on PDAC was shown in a supplementary EXCEL table named “**Table S3_cellular inhibitory rate of drug library**”. Detailed data of PDX#01/#02/#10 cell lines, PDO#01/PDO#02/PDO#07 lines and gemcitabine-resistant BxPC-3 were presented in seven sheets, respectively.

**Supplementary table4:** The list of the differential expression genes (DEGs) from RNA-sequencing data of PDO01# treated with vehicle and irbesartan, shown in a supplementary EXCEL table named “**Table S4_RNA sequencing of PDO01 treated with irbesartan**”.

**Supplementary table5:** Correlation of c-Jun expression to clinicopathological features in PDAC.

| **Parameters** |  | **c-Jun(n)** | | **χ^2^** | ***P*** | **r** |
| --- | --- | --- | --- | --- | --- | --- |
|  |  | **low** | **high** |  |  |  |
| **Age(years)** | ≤60 | 26 | 25 | 1.231 | 0.267 | 0.114 |
|  | >60 | 17 | 26 |  |  |  |
| **Gender** | Male | 27 | 32 | 0.000 | 0.996 | 0.000 |
|  | Female | 16 | 19 |  |  |  |
| **Histological grade** | G1, G2 | 34 | 29 | 5.205 | **0.023^a^** | 0.235 |
|  | G3 | 9 | 22 |  |  |  |
| **p TNM stage** | IA, IB | 31 | 26 | 4.357 | **0.037^a^** | 0.215 |
|  | IIA, IIB | 12 | 25 |  |  |  |
| **Tumor size** | T1≤3.5cm | 34 | 19 | 16.587 | **0.000^a^** | 0.420 |
|  | T2>3.5cm | 9 | 32 |  |  |  |
| **LN metastasis** | N0 | 33 | 41 | 0.185 | 0.667 | -0.044 |
|  | N1 | 10 | 10 |  |  |  |

Note: Data was based on IHC assay. Statistical data on c-Jun expression in relation to clinic- histopathologic features for surgical PDAC specimens. *P* values were calculated using the chi-square test. ^a^ Statistically significant (*P*<0.05). ^b^ Here c-Jun expression was divided into high-c-Jun and low-c-Jun according to staining scores.

**Supplementary table6:** Univariate and multivariate Cox proportional hazards analysis of clinicopathological factors for median overall survival and relapse free survival.

| **Variables** | **Overall survival** | | **Relapse free survival** | |
| --- | --- | --- | --- | --- |
| **Univariate analysis** | **HR (95.0% CI)** | ***P*** | **HR (95.0% CI)** | ***P*** |
| Age | 1.068(0.697-1.636) | 0.762 | 0.918(0.596-1.414) | 0.698 |
| Gender | 1.026(0.659-1.598) | 0.910 | 0.868(0.554-1.360) | 0.536 |
| Histological grade | 1.263(0.802-1.987) | 0.313 | 1.217(0.772-1.920) | 0.398 |
| Tumor size | 1.771(1.144-2.742) | **0.010^a^** | 1.710(1.102-2.653) | **0.017^a^** |
| p TNM stage | 2.103(1.355-3.262) | **0.001^a^** | 2.219(1.411-3.488) | **0.001^a^** |
| LN metastasis | 1.142(0.670-1.947) | 0.625 | 1.421(0.849-2.379) | 0.181 |
| c-Jun expression | 2.348(1.508-3.656) | **0.000^a^** | 1.934(1.248-2.999) | **0.003^a^** |
| **Multivariate analysis** |  |  |  |  |
| p TNM stage | 1.826(1.170-2.850) | **0.008^a^** | 2.026(1.283-3.201) | **0.002^a^** |
| c-Jun expression | 2.114(1.347-3.318) | **0.001^a^** | 1.753(1.123-2.735) | **0.013^a^** |

NOTE: Data was based on IHC assay. C-Jun staining score was determined by two independent pathologists who were blinded to the patients’ clinical features and outcomes. Multivariate Cox proportional hazards analysis used backward selection model. Abbreviations: HR, hazard ratio; CI, confidence interval; LN, lymph node. ^a^ Statistically significant (*P*<0.05).

**Supplementary table7:** The detailed information of the RNA-sequencing data of PDX#01#-vector/c-Jun-OE line was shown in a supplementary EXCEL table named **“Table S7_RNA-sequencing of PDX01-vector_c-Jun-OE”.**

**Supplementary table8:** The detailed information of the RNA-sequencing data of PDO#04#-vector/c-Jun-OE line was shown in a supplementary EXCEL table named **“Table S8_RNA-sequencing of PDO04-vector_c-Jun-OE”.**

**Supplementary table9:** The detailed information of signal pathway enrichment by GSEA from RNA-sequencing data of PDX#01-vector/c-Jun-OE and PDO#04-vector/c-Jun-OE was shown in a supplementary EXCEL table named **“Table S9_GSEA analysis by intersection of PDO04-vector_c-Jun and PDX01-vector_-c-Jun”.**

**Supplementary table10:** detailed information of shRNA oligos sequences for stable knockdown cells (the following oligos sequences were shown as: overhang-target-loop-antisense).

| Human c-Jun shRNA1#  (most efficient) | AAAAGGACCTTATGGCTACAGTATTGGATCCAATACTGTAGCCATAAGGTCC |
| --- | --- |
| Human c-Jun shRNA2# | AAAAGCTACAGTAACCCCAAGATTTGGATCCAAATCTTGGGGTTACTGTAGC |
| Human c-Jun shRNA3# | AAAAGGCACAGCTTAAACAGAAATTGGATCCAATTTCTGTTTAAGCTGTGCC |
| Human FTH1 shRNA1#  (most efficient) | AAAAGCCGAATCTTCCTTCAGGATATTGGATCCAATATCCTGAAGGAAGATTCGGC |
| Human FTH1 shRNA2# | AAAAGCGATGATGTGGCTTTGAATTGGATCCAATTCAAAGCCACATCATCGC |
| Human FTH1  shRNA3# | AAAAGCAGGTGAAAGCCATCAAATTGGATCCAATTTGATGGCTTTCACCTGC |
| Human FTL shRNA1#  (most efficient) | AAAACTGGAGACTCACTTCCTAGATTTGGATCCAAATCTAGGAAGTGAGTCTCCAG |
| Human FTL shRNA2# | AAAAGCCACTTCTTCCGCGAATTTTGGATCCAAAATTCGCGGAAGAAGTGGC |
| Human FTL shRNA3# | AAAAGGAGACTCACTTCCTAGATTTGGATCCAAATCTAGGAAGTGAGTCTCC |
| Human TFRC shRNA1#  (most efficient) | AAAAGCTAGATCAGCATTCTCTATTGGATCCAATAGAGAATGCTGATCTAGC |
| Human TFRC shRNA2# | AAAAGGTGGAGAACCATTGTCATTTGGATCCAAATGACAATGGTTCTCCACC |
| Human TFRC shRNA3# | AAAAGCAATTGGTGTGTTGATATTTGGATCCAAATATCAACACACCAATTGC |
| Human SOX9 shRNA1# (most efficient) | AAAAGGAGGAAGTCGGTGAAGAATTGGATCCAATTCTTCACCGACTTCCTCC |
| Human SOX9 shRNA2# | AAAAGCGACGTCATCTCCAACATTTGGATCCAAATGTTGGAGATGACGTCGC |
| Human SOX9 shRNA3# | AAAAGCAAGCTCTGGAGACTTCTGATTGGATCCAATCAGAAGTCTCCAGAGCTTGC |
| Human SOX2 shRNA1# (most efficient) | AAAAGGAGCACCCGGATTATAAATTGGATCCAATTTATAATCCGGGTGCTCC |
| Human SOX2 shRNA2# | AAAAGGGACATGATCAGCATGTATTGGATCCAATACATGCTGATCATGTCCC |
| Human SOX2 shRNA3# | AAAAGGACATGATCAGCATGTATTTGGATCCAAATACATGCTGATCATGTCC |
| Human OCT4 shRNA1# (most efficient) | AAAAGGTTCTATTTGGGAAGGTATTGGATCCAATACCTTCCCAAATAGAACC |
| Human OCT4 shRNA2# | AAAAGCTTCAAGAACATGTGTAATTGGATCCAATTACACATGTTCTTGAAGC |
| Human OCT4 shRNA3# | AAAAGCCCGAAAGAGAAAGCGAATTGGATCCAATTCGCTTTCTCTTTCGGGC |

**Supplementary table11:** detailed information of antibodies used in this study.

| **Antibodies name** | **Dilution** | **Source** | **Cat #** |
| --- | --- | --- | --- |
| Anti-human c-Jun antibody (for IHC, WB and Ch-IP) | 1:1000 | abcam | ab32137 |
| Anti-human Sox9 antibody (for WB and IHC) | 1:1000 | abcam | ab185230 |
| Anti-human Sox2 antibody (for WB and IHC) | 1:1000 | Proteintech | 66411-1-Ig |
| Anti-human Nanog antibody (for WB and IHC) | 1:1000 | abcam | ab109250 |
| Anti-human Oct4 antibody (for WB and IHC) | 1:1000 | abcam | ab18976 |
| Anti-human CD133 antibody (for mIHC and flow cytometry) | 1:100 | Miltenyi Biotec | 130-118-143 |
| Anti-human tubulin antibody (for WB) | 1:5000 | abmart | M20005 |
| Anti-human ALDH1 antibody (for mIHC) | 1:1000 | BD | 611194 |
| Anti-human CK19 antibody (for WB) | 1:1000 | abcam | ab7755 |
| Anti-human E-cadherin antibody (for WB) | 1:1000 | abcam | ab1416 |
| Anti-human Ki67 antibody (for IHC and mIHC) | 1:100 | abcam | ab16667 |
| Anti-human TP53 antibody (for IF) | 1:100 | abcam | ab32389 |
| Anti-human H3K27Ac antibody (for Ch-IP) | 1:100 | Merck | 07-360 |
| Anti-human H3K9me antibody (for Ch-IP) | 1:100 | abcam | ab8898 |
| PE/Cy7 anti-human EpCAM antibody (for flow cytometry) | 5μl/test | Biolegend | 324222 |
| PE anti-human CD24 antibody (for flow cytometry) | 5μl/test | Biolegend | 311106 |
| APC anti-human CD44 antibody (for flow cytometry) | 5μl/test | Biolegend | 338806 |
| Anti-human Ki67 antibody (for IHC) | 1:100 | abcam | ab16667 |
| Anti-human FTH1 antibody (for IHC and WB) | 1:1000 for WB; 1:100 for IHC | abcam | ab183781 |
| Anti-human FTL antibody (for IHC and WB) | 1:1000 for WB; 1:100 for IHC | abcam | ab218400 |
| Anti-human TFRC antibody (for IHC and WB) | 1:1000 for WB; 1:100 for IHC | abcam | ab214039 |
| Ant-human MST1 antibody (for WB) | 1:1000 | CST | CST#3682 |
| Anti-human LATS1 antibody (for WB) | 1:1000 | CST | CST#9153 |
| Anti-human p-MST1 antibody (for WB) | 1:1000 | CST | CST#49332 |
| Anti-human p-LATS1 antibody (for WB) | 1:1000 | CST | CST#9157 |
| Anti-human YAP1 antibody (for WB, IF and IHC) | 1:1000 for WB; 1:100 for IF and IHC | CST | CST#14074 |
| Anti-human p-YAP1(Ser127) antibody (for WB) | 1:1000 | CST | CST#13008 |
| Anti-human TAZ antibody (for WB) | 1:1000 | CST | CST#83669 |
| Anti-human TEAD1 antibody (for Ch-IP) | 1:100 | CST | CST#12292 |

**Supplementary table12:** detailed information of primers used for RT-PCR and Ch-IP in this study.

| **Gene name** | **Forward (5’-3’)** | **Reverse(5’-3’)** |
| --- | --- | --- |
| **h c-Jun** | TCCTGCCCAGTGTTGTTTGT | GACTTCTCAGTGGGCTGTCC |
| **h FTH1** | TTCAACAGTGCTTGGACGGA | AGTCACACAAATGGGGGTCAT |
| **h FTL** | AGATCCGGGGACTCTCTTCC | CAGCTGGCTTCTTGATGTCC |
| **h TFRC** | AGAACTACACCGACCCTCGT | TTTGAGCGCTGTCTTTGACC |
| **h SOX9** | AGGAAGCTCGCGGACCAGTAC | GGTGGTCCTTCTTGTGCTGCAC |
| **h SOX2** | GCTACAGCATGATGCAGGACCA | TCTGCGAGCTGGTCATGGAGTT |
| **h OCT4** | CCTGAAGCAGAAGAGGATCACC | AAAGCGGCAGATGGTCGTTTGG |
| **h Actin** | ACCCTGAAGTACCCCATCGAG | AGCACAGCCTGGATAGCAAC |
| **c-JunCh-IP-FTH1-1** | GGTTTAGCCATGTTAGTTAG | AATTTGGAGCAGAGTGAA |
| **c-JunCh-IP-FTL-1** | TCTGGGCTGAGGTATGAC | TGCTTTGTCTCCGTGTCT |
| **c-JunCh-IP-TFRC-1** | CCAACATGATAAAATCCCGTCA | TCCCCTGTGGTGTTCCTTCT |
| **c-JunCh-IP-TFRC-2** | AAACGGATTTGCTTCTTA | GGATTGTAGATTAACTTTCG |
| **c-JunCh-IP-TFRC-3** | ACATTCCATCCTGCACGTA | ACCGGCGGTTTATAGCCT |
| **c-JunCh-IP-SOX9-1** | TCTCCCGGAAGGACATTG | GTGAATCGGCCCACATCA |
| **c-JunCh-IP-SOX9-2** | ATGGATTATTACGGAGGAAC | TAATGCGTAGCCGAGTTT |
| **c-JunCh-IP-SOX2-1** | TTCTTAGGGTGAGTAAGGGTA | CTCTTGTTGGGATCTTTGTG |
| **c-JunCh-IP-SOX2-2** | AATAAATGGAACGTGGCT | TTGACTACAAAGGGTGGG |
| **c-JunCh-IP-SOX2-3** | AGGACAGAGGTTTGGGTCT | CAGCCAGTGCAGGAGTTG |
| **c-JunCh-IP-OCT4-1** | GTTGGGAGTTGAAAGTTGG | AGTGGCTGGGACTACAGG |
| **TEAD1Ch-IP-c-Jun-1** | ACACTCAAAGCCGCAAATAC | TCGCAGAGCCTGGATAGG |
| **TEAD1Ch-IP-c-Jun-2** | GTGCCCGGAGTGAAGAAA | GGGCTTGAAGTTGGACCTG |

**Supplementary table13:** detailed information of sequences of the plasmids used for luciferase analysis (The following DNA sequences were cloned into pGL3.0-basic plasmid).

| FTH1 promoter-binding site1-wt |
| --- |
| ttcacctgtcacccgccattaaccccgtcagcttctctccaatgacaccccagggttgtcctacagggccaccgaagcctgaagctcctgtgagtggggccgataagaggccattttgaaaagtcctcagcatgggcttcgcctttagatccacgtcgtttctcctttctaacccgagatgtgtcccttgggtgcgaagcgacaccagcgattctctcggaggccccggtggctcacccccacgactccccggggaggcggtccccatccctagggaaacccggcccgcccagcctttgttgcagaaaccgcacacggagccatatttgctttcagccaaaggcagacgtggtcggaggagtcggaagggcaggggcggacccgaaacccgccgtgcgccccacttctccatcgcgctccttcctggactctgaggaagagaaaatgcgctcgaccacctaaggaccgcgtctccaccttccaccttcccaatcatctgcagcttcccaatctgcagcaaggtgtcacaagggcttctccgcagctatgatttctaacaccatgccccgggccaggagcctcagggtgcccgttcgggaaaatgggagccgaatcaggatcaccccatgcgcccccgcacccttcccccgccggttccaacgcccgggcgcccctagaccggggggggggttctgaggtggacttcctgcgcctcctcgcagcttccctccaactccactaaacgggcacagagacgccaccgctgtcccagaggcagtcggctaccggtccccgctcccgagctccgccagagcgcgcgagggcctccagcggccgcccctcccccacagcaggggcggggtcccgcgcccaccggaaggagcgggctcggggcgggcggcgctgattggccggggcgggcctgacgccgacgcggctataagagaccacaagcgacccgcagg |
| FTH1 promoter-binding site1-mut |
| ttcacctgtcacccgccattaaccccgtcagcttctctccaatgacaccccagggttgtcctacagggccaccgaagcctgaagctcctgtgagtggggccttttttttttttttttgaaaagtcctcagcatgggcttcgcctttagatccacgtcgtttctcctttctaacccgagatgtgtcccttgggtgcgaagcgacaccagcgattctctcggaggccccggtggctcacccccacgactccccggggaggcggtccccatccctagggaaacccggcccgcccagcctttgttgcagaaaccgcacacggagccatatttgctttcagccaaaggcagacgtggtcggaggagtcggaagggcaggggcggacccgaaacccgccgtgcgccccacttctccatcgcgctccttcctggactctgaggaagagaaaatgcgctcgaccacctaaggaccgcgtctccaccttccaccttcccaatcatctgcagcttcccaatctgcagcaaggtgtcacaagggcttctccgcagctatgatttctaacaccatgccccgggccaggagcctcagggtgcccgttcgggaaaatgggagccgaatcaggatcaccccatgcgcccccgcacccttcccccgccggttccaacgcccgggcgcccctagaccggggggggggttctgaggtggacttcctgcgcctcctcgcagcttccctccaactccactaaacgggcacagagacgccaccgctgtcccagaggcagtcggctaccggtccccgctcccgagctccgccagagcgcgcgagggcctccagcggccgcccctcccccacagcaggggcggggtcccgcgcccaccggaaggagcgggctcggggcgggcggcgctgattggccggggcgggcctgacgccgacgcggctataagagaccacaagcgacccgcagg |
| FTL promoter-binding site1-wt |
| cagggtctctgtacccctctgtctccgggtctgttttcccctctctctgggtctctgtccccctctctctcagggtctctgtctgagtctctttttctctgggcctctgtctccccaccccacgttctctatggtcttcagatcctgtctctctcagcccctcctctccctccacctatgggccccaaggcccctctcggccgactaaaggtctctgggctgaggtatgacatttggtaaatgagtaactcgggccccacagtcaacggggtgctttggacggctgcccctcctccatggactcttcccagagaccccaagtcaggggagagagaggggcagaggatcaatgagtgtacactgagccaccacccagagtgaggggacaatatggccacaggcaggatcccagaaaaaaaaaaattatgggaaccaaaaagacacggagacaaagcacacagccggcttccaacaaggggggaccttggggtagggggcccgatttgggtctgagccaggtctagttctgagggtcccaccaaaaaagtcggggagtgccaaggggatgaggtcagcgagtggccccacccccatccgtccccgcgggggccccttcccgcgctgagctcagcatgactcagcagtcgctgcctgcaacaaggaccccagataccggcctggcggggcatctgcagatcccctcggtcaggggctgccccgcctgggctgttcggagcagctcctctgtagggcagggcctgacctcaccccacaccccagtggcttcagctccctgcccagcgcacgggctgcctccatcagggcacttaagtagagtcaaggtttgcgccgactccgtctcccagaaatcaacgtggaccaggcccacttaaaaaatatgcattagtcgaagacagacgtctcgtccataggtccccagactgtttgtccacaacgcagggcttctctttgtgggcctgaaggagcacacgtcaggcaagacacgagggcaacttcagagacactgttcagggccacggagaattctcgaagtctgtgagttgtctccaaaacagcctccaggaaatgcaatcccagagacaccgcaaagctcaaaacagacttttcattcctccccatttccactcccagaaaaagcctgggcccctgaaagagctccaaggtatccccaaagtcccagcccatctttggagggttccagagggctgtccagggttccagaagtcaccctacctcatacaatgctcctcctgggcttcccgcactgggtgaaacccacggctccagaataactgccctttccaacttgcagagcacctcaagaacctaaagatttccccggggagggctccaaaatatagagagtttgcatcgtgaacccccacccctgtaaatcccaggactccagtccccgcaacactccatcttccagagatctccaggggtccagaaataacacctcacagccttccaagcttcccaaggatccggagactccttatctcgcagcgcaaacctcaggcaactacaggtcctccctgcacctttgaggtgcccctccccccactcaaggttccagaaaaatccatgccctcggaaaccccagaatcttactttcccgaggcaccccaggaccccagggtcatcagttcacagggctgttagtgctcccataaaactctagcctcccagagggaccccagcccccaccctcccgcccacgaacccctgcatttccagaatcagccccagggccccaacccccccaagcccccatttcacaacacgctggcgctacaggcgcgtgacttccccttgctttggggcggggggctgagactcctatgtgctccggattggtcaggcacggccttcggccccgcctcctgccaccgcagattggccgctagccctccccgagcgccctgcctccgagggccggcgcaccataaaagaagccgccctagccacgtcccctc |
| FTL promoter-binding site1-mut |
| cagggtctctgtacccctctgtctccgggtctgttttcccctctctctgggtctctgtccccctctctctcagggtctctgtctgagtctctttttctctgggcctctgtctccccaccccacgttctctatggtcttcagatcctgtctctctcagcccctcctctccctccacctatgggccccaaggcccctctcggccgactaaaggtctctgggctgaggtatgacattttttttttttttttttcgggccccacagtcaacggggtgctttggacggctgcccctcctccatggactcttcccagagaccccaagtcaggggagagagaggggcagaggatcaatgagtgtacactgagccaccacccagagtgaggggacaatatggccacaggcaggatcccagaaaaaaaaaaattatgggaaccaaaaagacacggagacaaagcacacagccggcttccaacaaggggggaccttggggtagggggcccgatttgggtctgagccaggtctagttctgagggtcccaccaaaaaagtcggggagtgccaaggggatgaggtcagcgagtggccccacccccatccgtccccgcgggggccccttcccgcgctgagctcagcatgactcagcagtcgctgcctgcaacaaggaccccagataccggcctggcggggcatctgcagatcccctcggtcaggggctgccccgcctgggctgttcggagcagctcctctgtagggcagggcctgacctcaccccacaccccagtggcttcagctccctgcccagcgcacgggctgcctccatcagggcacttaagtagagtcaaggtttgcgccgactccgtctcccagaaatcaacgtggaccaggcccacttaaaaaatatgcattagtcgaagacagacgtctcgtccataggtccccagactgtttgtccacaacgcagggcttctctttgtgggcctgaaggagcacacgtcaggcaagacacgagggcaacttcagagacactgttcagggccacggagaattctcgaagtctgtgagttgtctccaaaacagcctccaggaaatgcaatcccagagacaccgcaaagctcaaaacagacttttcattcctccccatttccactcccagaaaaagcctgggcccctgaaagagctccaaggtatccccaaagtcccagcccatctttggagggttccagagggctgtccagggttccagaagtcaccctacctcatacaatgctcctcctgggcttcccgcactgggtgaaacccacggctccagaataactgccctttccaacttgcagagcacctcaagaacctaaagatttccccggggagggctccaaaatatagagagtttgcatcgtgaacccccacccctgtaaatcccaggactccagtccccgcaacactccatcttccagagatctccaggggtccagaaataacacctcacagccttccaagcttcccaaggatccggagactccttatctcgcagcgcaaacctcaggcaactacaggtcctccctgcacctttgaggtgcccctccccccactcaaggttccagaaaaatccatgccctcggaaaccccagaatcttactttcccgaggcaccccaggaccccagggtcatcagttcacagggctgttagtgctcccataaaactctagcctcccagagggaccccagcccccaccctcccgcccacgaacccctgcatttccagaatcagccccagggccccaacccccccaagcccccatttcacaacacgctggcgctacaggcgcgtgacttccccttgctttggggcggggggctgagactcctatgtgctccggattggtcaggcacggccttcggccccgcctcctgccaccgcagattggccgctagccctccccgagcgccctgcctccgagggccggcgcaccataaaagaagccgccctagccacgtcccctc |
| TFRC promoter-binding site1,2,3-wt |
| aatacaaaaattagcctggcatggtggcacacacctgtagtcccagctacttgggaggctgaggcaggagaatcgcttgaatccggaggcagaggttgcagtgagcccgagatcatgccactgtactccagccagggtgacagagcaagacactgtcttaaaagtaaaaataataaaaataaataaaattaataattaaagaaataaagaaggaacaccacaggggagcaggcctggtgtctgaatgttaaggtaggccctctgtggatgtgcatccttaaccttttcctgattagatcattaaaggtgcagcaatgctcctgctcatatgacttttttttttttttgagatggaatcttgctctgtcgcccaggttggagtgcaatggcgcgatcttggctcactgcaacctccgcctcttgggttcaagcgattcttctgcctcagcctcctgagtagctgggattacaggcgcgtgccactatgcctggctaattttttttgaaatttttttagtagagatggggtttcaccatattggtcaggctggtctcgaactccagacctcgtgatccgctcacctcggcctcccaaagtgttaggattgcaggcgtgagccaccgcgcctggcaactcatatgacttttaaacacttctctttggggagagagattttagagaatgggagtaaagagaaaggcggtgagaatcccaagtactttctctatcttagagcagaagaaataaagggaagagtaaaagcccaaggcccagaaacggatttgcttcttaaagggctgttctgtgtccctacgggcctgaaggtcagtttatgtgcaatgctttttaaggtatgggttgctttcttgcattgtagctactacctccctggcctgctggttactgaccagagggagggagagggtgtgattgtttggaaagagtttaaacagtgacaagaattgaattttatttatttgttattcattgcacaatccaataacagcgatggcaatgaaaacgaaagttaatctacaatcctatcaccctattaagtcacttgtttctattttcccttttattccctcatgcattaaacattacgcaaagcactccgctagtcttggttaaagtgcagcttgtatacattctgaataaaaatttgcattttgcttttcttttcctttttgtatttctgaggctgcaaaatacatcttcacaagagtggctttaatagttacctaacattccatcctgcacgtacatttcaaggcactgccgcagtgcaatatccaacatttaagcgtcaacgccaacagattggacctagcactgcagggactggaggttggtcaaggtcaggcggaggctgccaggctaccagggtggaggaaggcgccgaggcagaggccagtgcgcccatcgcgcggctcctcggggcacctgctgccttggcgccttttcccttggccttcgcctcgcccgcagcgccctccgcatagggccccgcccgctgcgcgcgcatccccgccccccgggcgatctgtcagagcacctcgcgagcgtacgtgcctcaggaagtgacgcacagcccccctgggggccgggggcggggccaggctataaaccgccggttaggggccgccatcccctc |
| TFRC promoter-binding site1-mut |
| aatacaaaaattagcctggcatggtggcacacacctgtagtcccagctacttgggaggctgaggcaggagaatcgcttgaatccggaggcagaggttgcagtgagcccttttttttttttttgtactccagccagggtgacagagcaagacactgtcttaaaagtaaaaataataaaaataaataaaattaataattaaagaaataaagaaggaacaccacaggggagcaggcctggtgtctgaatgttaaggtaggccctctgtggatgtgcatccttaaccttttcctgattagatcattaaaggtgcagcaatgctcctgctcatatgacttttttttttttttgagatggaatcttgctctgtcgcccaggttggagtgcaatggcgcgatcttggctcactgcaacctccgcctcttgggttcaagcgattcttctgcctcagcctcctgagtagctgggattacaggcgcgtgccactatgcctggctaattttttttgaaatttttttagtagagatggggtttcaccatattggtcaggctggtctcgaactccagacctcgtgatccgctcacctcggcctcccaaagtgttaggattgcaggcgtgagccaccgcgcctggcaactcatatgacttttaaacacttctctttggggagagagattttagagaatgggagtaaagagaaaggcggtgagaatcccaagtactttctctatcttagagcagaagaaataaagggaagagtaaaagcccaaggcccagaaacggatttgcttcttaaagggctgttctgtgtccctacgggcctgaaggtcagtttatgtgcaatgctttttaaggtatgggttgctttcttgcattgtagctactacctccctggcctgctggttactgaccagagggagggagagggtgtgattgtttggaaagagtttaaacagtgacaagaattgaattttatttatttgttattcattgcacaatccaataacagcgatggcaatgaaaacgaaagttaatctacaatcctatcaccctattaagtcacttgtttctattttcccttttattccctcatgcattaaacattacgcaaagcactccgctagtcttggttaaagtgcagcttgtatacattctgaataaaaatttgcattttgcttttcttttcctttttgtatttctgaggctgcaaaatacatcttcacaagagtggctttaatagttacctaacattccatcctgcacgtacatttcaaggcactgccgcagtgcaatatccaacatttaagcgtcaacgccaacagattggacctagcactgcagggactggaggttggtcaaggtcaggcggaggctgccaggctaccagggtggaggaaggcgccgaggcagaggccagtgcgcccatcgcgcggctcctcggggcacctgctgccttggcgccttttcccttggccttcgcctcgcccgcagcgccctccgcatagggccccgcccgctgcgcgcgcatccccgccccccgggcgatctgtcagagcacctcgcgagcgtacgtgcctcaggaagtgacgcacagcccccctgggggccgggggcggggccaggctataaaccgccggttaggggccgccatcccctc |
| TFRC promoter-binding site2-mut |
| aatacaaaaattagcctggcatggtggcacacacctgtagtcccagctacttgggaggctgaggcaggagaatcgcttgaatccggaggcagaggttgcagtgagcccgagatcatgccactgtactccagccagggtgacagagcaagacactgtcttaaaagtaaaaataataaaaataaataaaattaataattaaagaaataaagaaggaacaccacaggggagcaggcctggtgtctgaatgttaaggtaggccctctgtggatgtgcatccttaaccttttcctgattagatcattaaaggtgcagcaatgctcctgctcatatgacttttttttttttttgagatggaatcttgctctgtcgcccaggttggagtgcaatggcgcgatcttggctcactgcaacctccgcctcttgggttcaagcgattcttctgcctcagcctcctgagtagctgggattacaggcgcgtgccactatgcctggctaattttttttgaaatttttttagtagagatggggtttcaccatattggtcaggctggtctcgaactccagacctcgtgatccgctcacctcggcctcccaaagtgttaggattgcaggcgtgagccaccgcgcctggcaactcatatgacttttaaacacttctctttggggagagagattttagagaatgggagtaaagagaaaggcggtgagaatcccaagtactttctctatcttagagcagaagaaataaagggaagagtaaaagcccaaggcccagaaacggatttgcttcttaaagggctgttctgtgtccctacgggcctgaaggtcagtttatgtgcaatgctttttaaggtatgggttgctttcttgcattgtagctactacctccctggcctgctggttactgaccagagggagggagagggtgtgattgtttggaaagagtttaaacagtgacaagaattgaattttatttatttttttttttttttttaatccaataacagcgatggcaatgaaaacgaaagttaatctacaatcctatcaccctattaagtcacttgtttctattttcccttttattccctcatgcattaaacattacgcaaagcactccgctagtcttggttaaagtgcagcttgtatacattctgaataaaaatttgcattttgcttttcttttcctttttgtatttctgaggctgcaaaatacatcttcacaagagtggctttaatagttacctaacattccatcctgcacgtacatttcaaggcactgccgcagtgcaatatccaacatttaagcgtcaacgccaacagattggacctagcactgcagggactggaggttggtcaaggtcaggcggaggctgccaggctaccagggtggaggaaggcgccgaggcagaggccagtgcgcccatcgcgcggctcctcggggcacctgctgccttggcgccttttcccttggccttcgcctcgcccgcagcgccctccgcatagggccccgcccgctgcgcgcgcatccccgccccccgggcgatctgtcagagcacctcgcgagcgtacgtgcctcaggaagtgacgcacagcccccctgggggccgggggcggggccaggctataaaccgccggttaggggccgccatcccctc |
| TFRC promoter-binding site3-mut |
| aatacaaaaattagcctggcatggtggcacacacctgtagtcccagctacttgggaggctgaggcaggagaatcgcttgaatccggaggcagaggttgcagtgagcccgagatcatgccactgtactccagccagggtgacagagcaagacactgtcttaaaagtaaaaataataaaaataaataaaattaataattaaagaaataaagaaggaacaccacaggggagcaggcctggtgtctgaatgttaaggtaggccctctgtggatgtgcatccttaaccttttcctgattagatcattaaaggtgcagcaatgctcctgctcatatgacttttttttttttttgagatggaatcttgctctgtcgcccaggttggagtgcaatggcgcgatcttggctcactgcaacctccgcctcttgggttcaagcgattcttctgcctcagcctcctgagtagctgggattacaggcgcgtgccactatgcctggctaattttttttgaaatttttttagtagagatggggtttcaccatattggtcaggctggtctcgaactccagacctcgtgatccgctcacctcggcctcccaaagtgttaggattgcaggcgtgagccaccgcgcctggcaactcatatgacttttaaacacttctctttggggagagagattttagagaatgggagtaaagagaaaggcggtgagaatcccaagtactttctctatcttagagcagaagaaataaagggaagagtaaaagcccaaggcccagaaacggatttgcttcttaaagggctgttctgtgtccctacgggcctgaaggtcagtttatgtgcaatgctttttaaggtatgggttgctttcttgcattgtagctactacctccctggcctgctggttactgaccagagggagggagagggtgtgattgtttggaaagagtttaaacagtgacaagaattgaattttatttatttgttattcattgcacaatccaataacagcgatggcaatgaaaacgaaagttaatctacaatcctatcaccctattaagtcacttgtttctattttcccttttattccctcatgcattaaacattacgcaaagcactccgctagtcttggttaaagtgcagcttgtatacattctgaataaaaatttgcattttgcttttcttttcctttttgtatttctgaggctgcaaaatacatcttcacaagagtggctttaatagttacctaacattccatcctgcacgtacatttcaaggcactgccgcagtgcaatatccaacatttaagcgtcaacgccaacagattggacctagcactgcagggactggaggttggtcaaggtcaggcggaggctgccaggctaccagggtggaggaaggcgccgaggcagaggccagtgcgcccatcgcgcggctcctcggggcacctgctgccttggcgccttttcccttggccttcgcctcgcccgcagcgccctccgcatagggccccgcccgctgcgcgcgcatccccgccccccgggcgatctgtcagagcacctcgcgagcgtacgtgcctcttttttttttttttagcccccctgggggccgggggcggggccaggctataaaccgccggttaggggccgccatcccctc |
| TFRC promoter-binding site1+2-mut |
| aatacaaaaattagcctggcatggtggcacacacctgtagtcccagctacttgggaggctgaggcaggagaatcgcttgaatccggaggcagaggttgcagtgagcccttttttttttttttgtactccagccagggtgacagagcaagacactgtcttaaaagtaaaaataataaaaataaataaaattaataattaaagaaataaagaaggaacaccacaggggagcaggcctggtgtctgaatgttaaggtaggccctctgtggatgtgcatccttaaccttttcctgattagatcattaaaggtgcagcaatgctcctgctcatatgacttttttttttttttgagatggaatcttgctctgtcgcccaggttggagtgcaatggcgcgatcttggctcactgcaacctccgcctcttgggttcaagcgattcttctgcctcagcctcctgagtagctgggattacaggcgcgtgccactatgcctggctaattttttttgaaatttttttagtagagatggggtttcaccatattggtcaggctggtctcgaactccagacctcgtgatccgctcacctcggcctcccaaagtgttaggattgcaggcgtgagccaccgcgcctggcaactcatatgacttttaaacacttctctttggggagagagattttagagaatgggagtaaagagaaaggcggtgagaatcccaagtactttctctatcttagagcagaagaaataaagggaagagtaaaagcccaaggcccagaaacggatttgcttcttaaagggctgttctgtgtccctacgggcctgaaggtcagtttatgtgcaatgctttttaaggtatgggttgctttcttgcattgtagctactacctccctggcctgctggttactgaccagagggagggagagggtgtgattgtttggaaagagtttaaacagtgacaagaattgaattttatttatttttttttttttttttaatccaataacagcgatggcaatgaaaacgaaagttaatctacaatcctatcaccctattaagtcacttgtttctattttcccttttattccctcatgcattaaacattacgcaaagcactccgctagtcttggttaaagtgcagcttgtatacattctgaataaaaatttgcattttgcttttcttttcctttttgtatttctgaggctgcaaaatacatcttcacaagagtggctttaatagttacctaacattccatcctgcacgtacatttcaaggcactgccgcagtgcaatatccaacatttaagcgtcaacgccaacagattggacctagcactgcagggactggaggttggtcaaggtcaggcggaggctgccaggctaccagggtggaggaaggcgccgaggcagaggccagtgcgcccatcgcgcggctcctcggggcacctgctgccttggcgccttttcccttggccttcgcctcgcccgcagcgccctccgcatagggccccgcccgctgcgcgcgcatccccgccccccgggcgatctgtcagagcacctcgcgagcgtacgtgcctcaggaagtgacgcacagcccccctgggggccgggggcggggccaggctataaaccgccggttaggggccgccatcccctc |
| TFRC promoter-binding site1+3-mut |
| aatacaaaaattagcctggcatggtggcacacacctgtagtcccagctacttgggaggctgaggcaggagaatcgcttgaatccggaggcagaggttgcagtgagcccttttttttttttttgtactccagccagggtgacagagcaagacactgtcttaaaagtaaaaataataaaaataaataaaattaataattaaagaaataaagaaggaacaccacaggggagcaggcctggtgtctgaatgttaaggtaggccctctgtggatgtgcatccttaaccttttcctgattagatcattaaaggtgcagcaatgctcctgctcatatgacttttttttttttttgagatggaatcttgctctgtcgcccaggttggagtgcaatggcgcgatcttggctcactgcaacctccgcctcttgggttcaagcgattcttctgcctcagcctcctgagtagctgggattacaggcgcgtgccactatgcctggctaattttttttgaaatttttttagtagagatggggtttcaccatattggtcaggctggtctcgaactccagacctcgtgatccgctcacctcggcctcccaaagtgttaggattgcaggcgtgagccaccgcgcctggcaactcatatgacttttaaacacttctctttggggagagagattttagagaatgggagtaaagagaaaggcggtgagaatcccaagtactttctctatcttagagcagaagaaataaagggaagagtaaaagcccaaggcccagaaacggatttgcttcttaaagggctgttctgtgtccctacgggcctgaaggtcagtttatgtgcaatgctttttaaggtatgggttgctttcttgcattgtagctactacctccctggcctgctggttactgaccagagggagggagagggtgtgattgtttggaaagagtttaaacagtgacaagaattgaattttatttatttgttattcattgcacaatccaataacagcgatggcaatgaaaacgaaagttaatctacaatcctatcaccctattaagtcacttgtttctattttcccttttattccctcatgcattaaacattacgcaaagcactccgctagtcttggttaaagtgcagcttgtatacattctgaataaaaatttgcattttgcttttcttttcctttttgtatttctgaggctgcaaaatacatcttcacaagagtggctttaatagttacctaacattccatcctgcacgtacatttcaaggcactgccgcagtgcaatatccaacatttaagcgtcaacgccaacagattggacctagcactgcagggactggaggttggtcaaggtcaggcggaggctgccaggctaccagggtggaggaaggcgccgaggcagaggccagtgcgcccatcgcgcggctcctcggggcacctgctgccttggcgccttttcccttggccttcgcctcgcccgcagcgccctccgcatagggccccgcccgctgcgcgcgcatccccgccccccgggcgatctgtcagagcacctcgcgagcgtacgtgcctcttttttttttttttagcccccctgggggccgggggcggggccaggctataaaccgccggttaggggccgccatcccctc |
| TFRC promoter-binding site2+3-mut |
| aatacaaaaattagcctggcatggtggcacacacctgtagtcccagctacttgggaggctgaggcaggagaatcgcttgaatccggaggcagaggttgcagtgagcccgagatcatgccactgtactccagccagggtgacagagcaagacactgtcttaaaagtaaaaataataaaaataaataaaattaataattaaagaaataaagaaggaacaccacaggggagcaggcctggtgtctgaatgttaaggtaggccctctgtggatgtgcatccttaaccttttcctgattagatcattaaaggtgcagcaatgctcctgctcatatgacttttttttttttttgagatggaatcttgctctgtcgcccaggttggagtgcaatggcgcgatcttggctcactgcaacctccgcctcttgggttcaagcgattcttctgcctcagcctcctgagtagctgggattacaggcgcgtgccactatgcctggctaattttttttgaaatttttttagtagagatggggtttcaccatattggtcaggctggtctcgaactccagacctcgtgatccgctcacctcggcctcccaaagtgttaggattgcaggcgtgagccaccgcgcctggcaactcatatgacttttaaacacttctctttggggagagagattttagagaatgggagtaaagagaaaggcggtgagaatcccaagtactttctctatcttagagcagaagaaataaagggaagagtaaaagcccaaggcccagaaacggatttgcttcttaaagggctgttctgtgtccctacgggcctgaaggtcagtttatgtgcaatgctttttaaggtatgggttgctttcttgcattgtagctactacctccctggcctgctggttactgaccagagggagggagagggtgtgattgtttggaaagagtttaaacagtgacaagaattgaattttatttatttttttttttttttttaatccaataacagcgatggcaatgaaaacgaaagttaatctacaatcctatcaccctattaagtcacttgtttctattttcccttttattccctcatgcattaaacattacgcaaagcactccgctagtcttggttaaagtgcagcttgtatacattctgaataaaaatttgcattttgcttttcttttcctttttgtatttctgaggctgcaaaatacatcttcacaagagtggctttaatagttacctaacattccatcctgcacgtacatttcaaggcactgccgcagtgcaatatccaacatttaagcgtcaacgccaacagattggacctagcactgcagggactggaggttggtcaaggtcaggcggaggctgccaggctaccagggtggaggaaggcgccgaggcagaggccagtgcgcccatcgcgcggctcctcggggcacctgctgccttggcgccttttcccttggccttcgcctcgcccgcagcgccctccgcatagggccccgcccgctgcgcgcgcatccccgccccccgggcgatctgtcagagcacctcgcgagcgtacgtgcctcttttttttttttttagcccccctgggggccgggggcggggccaggctataaaccgccggttaggggccgccatcccctc |
| TFRC promoter-binding site1+2+3-mut |
| aatacaaaaattagcctggcatggtggcacacacctgtagtcccagctacttgggaggctgaggcaggagaatcgcttgaatccggaggcagaggttgcagtgagcccttttttttttttttgtactccagccagggtgacagagcaagacactgtcttaaaagtaaaaataataaaaataaataaaattaataattaaagaaataaagaaggaacaccacaggggagcaggcctggtgtctgaatgttaaggtaggccctctgtggatgtgcatccttaaccttttcctgattagatcattaaaggtgcagcaatgctcctgctcatatgacttttttttttttttgagatggaatcttgctctgtcgcccaggttggagtgcaatggcgcgatcttggctcactgcaacctccgcctcttgggttcaagcgattcttctgcctcagcctcctgagtagctgggattacaggcgcgtgccactatgcctggctaattttttttgaaatttttttagtagagatggggtttcaccatattggtcaggctggtctcgaactccagacctcgtgatccgctcacctcggcctcccaaagtgttaggattgcaggcgtgagccaccgcgcctggcaactcatatgacttttaaacacttctctttggggagagagattttagagaatgggagtaaagagaaaggcggtgagaatcccaagtactttctctatcttagagcagaagaaataaagggaagagtaaaagcccaaggcccagaaacggatttgcttcttaaagggctgttctgtgtccctacgggcctgaaggtcagtttatgtgcaatgctttttaaggtatgggttgctttcttgcattgtagctactacctccctggcctgctggttactgaccagagggagggagagggtgtgattgtttggaaagagtttaaacagtgacaagaattgaattttatttatttttttttttttttttaatccaataacagcgatggcaatgaaaacgaaagttaatctacaatcctatcaccctattaagtcacttgtttctattttcccttttattccctcatgcattaaacattacgcaaagcactccgctagtcttggttaaagtgcagcttgtatacattctgaataaaaatttgcattttgcttttcttttcctttttgtatttctgaggctgcaaaatacatcttcacaagagtggctttaatagttacctaacattccatcctgcacgtacatttcaaggcactgccgcagtgcaatatccaacatttaagcgtcaacgccaacagattggacctagcactgcagggactggaggttggtcaaggtcaggcggaggctgccaggctaccagggtggaggaaggcgccgaggcagaggccagtgcgcccatcgcgcggctcctcggggcacctgctgccttggcgccttttcccttggccttcgcctcgcccgcagcgccctccgcatagggccccgcccgctgcgcgcgcatccccgccccccgggcgatctgtcagagcacctcgcgagcgtacgtgcctcttttttttttttttagcccccctgggggccgggggcggggccaggctataaaccgccggttaggggccgccatcccctc |
| SOX9 promoter-binding site1+2-wt |
| aaagtcatagccctcttcactgactttattccagcaaacatggtaattacaatatcatacaactaagtacagacgacctggctaaaatgtctgcccgatggtctccgagtctcctgaatcaggtgtctgagaattacacagttatactgtacacacaatgcccttctacccgggacacagaaataggtccacactacgcggacttttttctcctaggaaaggacgatgctgttcttacactttctgaaagtaatcacagagccctggatacgaagctattgtatgcaaatctcttaaatttgtaaacgagctatagggcaccagaaacatcccatttgaagaagttacattcgttaaaaaaaaaatgctgttgaacaaggctgtaacttactaccttatgaagagttccatttcctataagaaatgtcgggcttcttccgaagacaacgagagaaaacaagattttaagaaattctcccggaaggacattgatttggatcttgtgatagtgtcctcacttcgcaaattagaaagggaaaaaaaaactagtttttattatgatgtgggccgattcaccacaacaataatttaattgaggcgaatttttgcaagagcccaaaagggtggggggggggggggagtttaaaattaagagtttcccaatgctgtgcgtttatttgggattctgaaagcacagaacccgcaagcgaccaagacttttcttctatcccagagcagatagctccgcacttacccaacctggctctaagcatttcgtgtaaacacaaaggttgtgctcaaatcacacttgaaatacatgagagacaccaccaatgcctcccccagaactcccaactacatgcaggtctgaaccgaccggactagagtcagaagcaccggcgcttcaccccttcaggtgtttcgtaaatgccagcaaaagcaggcaagcagcatgactccgccagagtggagcgttttgtctgcggtggtgcccatttgtttggtcttttacaaaccaagtgaccggcctgggcctcgcggcccgggacagccgcattggcaaacttctatctctcaaagccagagcagttagcaaactctcccccagacagggcgactcggctgacgtttttgacccggccaggaggcaaagaccaaaacgtcagagcagtagccctgttactgaggagcgtcggcagggtcgcgggtagagggggctggagaatgacttgtcagagctcaaggtcgatgtggcgcggggcggcctcgagagcgccgggctcctgcgtggccacggccgccgctgccaaccttcgcggggacttagctttgctttccattgactccctttgcaaaagcgcagcagaatcctgaccagccgcaccagccccggcgaacccgagcatgttaatctatttatatggattattacggaggaacagcgggcgttgagtcaccaaaacatttgcttcaaaagactatttctaagcacttttgcaggcaggcaggctcgctccaggcgcgtaaactcggctacgcattaagaagcggctgcttttcgaatactgcaaactccagctaagtccccggtgccgcggagagagcagtgaaaagaaatgtcggaggtgggggtagatcctagtctagacacacacacttgcgcgcacacacacacacacacacacaagattcgcgcggagaaggcactaaaattctggcattccgagagtacgacaaacttacacacttggaagtcccgggtcccccgccttccccgcagcaccccccgcccccccaccctaccgtccgccctttggctgcgatcccctcccctctcctcccctcccgcctcgtcacccagcccagtgccacaatcctcctccctccccaaaatcgggtccaatcagctgcctgccaaccctgggactgctgtgctgtgattggcgggtggctctaaggtgaggcggagtatttattaaagagaccctgggctgggagtt |
| SOX9 promoter-binding site1-mut |
| aaagtcatagccctcttcactgactttattccagcaaacatggtaattacaatatcatacaactaagtacagacgacctggctaaaatgtctgcccgatggtctccgagtctcctgaatcaggtgtctgagaattacacagttatactgtacacacaatgcccttctacccgggacacagaaataggtccacactacgcggacttttttctcctaggaaaggacgatgctgttcttacactttctgaaagtaatcacagagccctggatacgaagctattgtatgcaaatctcttaaatttgtaaacgagctatagggcaccagaaacatcccatttgaagaagttacattcgttaaaaaaaaaatgctgttgaacaaggctgtaacttactaccttatgaagagttccatttcctataagaaatgtcgggcttcttccgaagacaacgagagaaaacaagattttaagaaattctcccggaaggacattgatttggatcttgtgatagtgtcctcacttcgcaaattagaaagggaaaaaaaaactagtttttattatgatgtgggccgattcaccacaacaataatttaattgaggcgaatttttgcaagagcccaaaagggtggggggggggggggagtttaaaattaagagtttcccaatgctgtgcgtttatttgggattctgaaagcacagaacccgcaagcgaccaagacttttcttctatcccagagcagatagctccgcacttacccaacctggctctaagcatttcgtgtaaacacaaaggttgtgctcaaatcacacttgaaatacatgagagacaccaccaatgcctcccccagaactcccaactacatgcaggtctgaaccgaccggactagagtcagaagcaccggcgcttcaccccttcaggtgtttcgtaaatgccagcaaaagcaggcaagcagcatgactccgccagagtggagcgttttgtctgcggtggtgcccatttgtttggtcttttacaaaccaagtgaccggcctgggcctcgcggcccgggacagccgcattggcaaacttctatctctcaaagccagagcagttagcaaactctcccccagacagggcgactcggctgacgtttttgacccggccaggaggcaaagaccaaaacgtcagagcagtagccctgttactgaggagcgtcggcagggtcgcgggtagagggggctggagaatgacttgtcagagctcaaggtcgatgtggcgcggggcggcctcgagagcgccgggctcctgcgtggccacggccgccgctgccaaccttcgcggggacttagctttgctttccattgactccctttgcaaaagcgcagcagaatcctgaccagccgcaccagccccggcgaacccgagcatgttaatctatttatatggattattacggaggaacagcgggcgttttttttttttttcatttgcttcaaaagactatttctaagcacttttgcaggcaggcaggctcgctccaggcgcgtaaactcggctacgcattaagaagcggctgcttttcgaatactgcaaactccagctaagtccccggtgccgcggagagagcagtgaaaagaaatgtcggaggtgggggtagatcctagtctagacacacacacttgcgcgcacacacacacacacacacacaagattcgcgcggagaaggcactaaaattctggcattccgagagtacgacaaacttacacacttggaagtcccgggtcccccgccttccccgcagcaccccccgcccccccaccctaccgtccgccctttggctgcgatcccctcccctctcctcccctcccgcctcgtcacccagcccagtgccacaatcctcctccctccccaaaatcgggtccaatcagctgcctgccaaccctgggactgctgtgctgtgattggcgggtggctctaaggtgaggcggagtatttattaaagagaccctgggctgggagtt |
| SOX9 promoter-binding site2-mut |
| aaagtcatagccctcttcactgactttattccagcaaacatggtaattacaatatcatacaactaagtacagacgacctggctaaaatgtctgcccgatggtctccgagtctcctgaatcaggtgtctgagaattacacagttatactgtacacacaatgcccttctacccgggacacagaaataggtccacactacgcggacttttttctcctaggaaaggacgatgctgttcttacactttctgaaagtaatcacagagccctggatacgaagctattgtatgcaaatctcttaaatttgtaaacgagctatagggcaccagaaacatcccatttgaagaagttacattcgttaaaaaaaaaatgctgttgaacaaggctgtaacttactaccttatgaagagttccatttcctataagaaatgtcgggcttcttccgaagacaacgagagaaaacaagattttaagaaattctcccggaaggacattgatttggatcttgtgatatttttttttttttgcaaattagaaagggaaaaaaaaactagtttttattatgatgtgggccgattcaccacaacaataatttaattgaggcgaatttttgcaagagcccaaaagggtggggggggggggggagtttaaaattaagagtttcccaatgctgtgcgtttatttgggattctgaaagcacagaacccgcaagcgaccaagacttttcttctatcccagagcagatagctccgcacttacccaacctggctctaagcatttcgtgtaaacacaaaggttgtgctcaaatcacacttgaaatacatgagagacaccaccaatgcctcccccagaactcccaactacatgcaggtctgaaccgaccggactagagtcagaagcaccggcgcttcaccccttcaggtgtttcgtaaatgccagcaaaagcaggcaagcagcatgactccgccagagtggagcgttttgtctgcggtggtgcccatttgtttggtcttttacaaaccaagtgaccggcctgggcctcgcggcccgggacagccgcattggcaaacttctatctctcaaagccagagcagttagcaaactctcccccagacagggcgactcggctgacgtttttgacccggccaggaggcaaagaccaaaacgtcagagcagtagccctgttactgaggagcgtcggcagggtcgcgggtagagggggctggagaatgacttgtcagagctcaaggtcgatgtggcgcggggcggcctcgagagcgccgggctcctgcgtggccacggccgccgctgccaaccttcgcggggacttagctttgctttccattgactccctttgcaaaagcgcagcagaatcctgaccagccgcaccagccccggcgaacccgagcatgttaatctatttatatggattattacggaggaacagcgggcgttgagtcaccaaaacatttgcttcaaaagactatttctaagcacttttgcaggcaggcaggctcgctccaggcgcgtaaactcggctacgcattaagaagcggctgcttttcgaatactgcaaactccagctaagtccccggtgccgcggagagagcagtgaaaagaaatgtcggaggtgggggtagatcctagtctagacacacacacttgcgcgcacacacacacacacacacacaagattcgcgcggagaaggcactaaaattctggcattccgagagtacgacaaacttacacacttggaagtcccgggtcccccgccttccccgcagcaccccccgcccccccaccctaccgtccgccctttggctgcgatcccctcccctctcctcccctcccgcctcgtcacccagcccagtgccacaatcctcctccctccccaaaatcgggtccaatcagctgcctgccaaccctgggactgctgtgctgtgattggcgggtggctctaaggtgaggcggagtatttattaaagagaccctgggctgggagtt |
| SOX9 promoter-binding site1+2-mut |
| aaagtcatagccctcttcactgactttattccagcaaacatggtaattacaatatcatacaactaagtacagacgacctggctaaaatgtctgcccgatggtctccgagtctcctgaatcaggtgtctgagaattacacagttatactgtacacacaatgcccttctacccgggacacagaaataggtccacactacgcggacttttttctcctaggaaaggacgatgctgttcttacactttctgaaagtaatcacagagccctggatacgaagctattgtatgcaaatctcttaaatttgtaaacgagctatagggcaccagaaacatcccatttgaagaagttacattcgttaaaaaaaaaatgctgttgaacaaggctgtaacttactaccttatgaagagttccatttcctataagaaatgtcgggcttcttccgaagacaacgagagaaaacaagattttaagaaattctcccggaaggacattgatttggatcttgtgatatttttttttttttgcaaattagaaagggaaaaaaaaactagtttttattatgatgtgggccgattcaccacaacaataatttaattgaggcgaatttttgcaagagcccaaaagggtggggggggggggggagtttaaaattaagagtttcccaatgctgtgcgtttatttgggattctgaaagcacagaacccgcaagcgaccaagacttttcttctatcccagagcagatagctccgcacttacccaacctggctctaagcatttcgtgtaaacacaaaggttgtgctcaaatcacacttgaaatacatgagagacaccaccaatgcctcccccagaactcccaactacatgcaggtctgaaccgaccggactagagtcagaagcaccggcgcttcaccccttcaggtgtttcgtaaatgccagcaaaagcaggcaagcagcatgactccgccagagtggagcgttttgtctgcggtggtgcccatttgtttggtcttttacaaaccaagtgaccggcctgggcctcgcggcccgggacagccgcattggcaaacttctatctctcaaagccagagcagttagcaaactctcccccagacagggcgactcggctgacgtttttgacccggccaggaggcaaagaccaaaacgtcagagcagtagccctgttactgaggagcgtcggcagggtcgcgggtagagggggctggagaatgacttgtcagagctcaaggtcgatgtggcgcggggcggcctcgagagcgccgggctcctgcgtggccacggccgccgctgccaaccttcgcggggacttagctttgctttccattgactccctttgcaaaagcgcagcagaatcctgaccagccgcaccagccccggcgaacccgagcatgttaatctatttatatggattattacggaggaacagcgggcgttttttttttttttcatttgcttcaaaagactatttctaagcacttttgcaggcaggcaggctcgctccaggcgcgtaaactcggctacgcattaagaagcggctgcttttcgaatactgcaaactccagctaagtccccggtgccgcggagagagcagtgaaaagaaatgtcggaggtgggggtagatcctagtctagacacacacacttgcgcgcacacacacacacacacacacaagattcgcgcggagaaggcactaaaattctggcattccgagagtacgacaaacttacacacttggaagtcccgggtcccccgccttccccgcagcaccccccgcccccccaccctaccgtccgccctttggctgcgatcccctcccctctcctcccctcccgcctcgtcacccagcccagtgccacaatcctcctccctccccaaaatcgggtccaatcagctgcctgccaaccctgggactgctgtgctgtgattggcgggtggctctaaggtgaggcggagtatttattaaagagaccctgggctgggagtt |
| SOX2 promoter-binding site1+2+3-wt |
| agaactaaaacaagccataacttgagagaaaaaggagaaccttcggggggcaggaaggttgattggaaataacttaaggaaagtctgcagaattcttttttttacaacttttctgagtttccagtgggtatatttagtgtgagtttgacagtaacaggctagggagggcagagattggagaaattgggggtcgggggagtgattatgggaagaaggttagtaaggaacaaaacaatgcaccgttttgtaaagataataaatggaacgtggctggtagatactattcagtacattttcttagggtgagtaagggtagaccaggggaggagggggcggagagagtgttacagaagaaagaaaataagtaaccctgatggtttaagccctttataaaaaagaaatggcatcaggtttttttttctttattcccccccaccccaccctttgtagtcaagtgcattttagccacaaagatcccaacaagagagtggaaggaaacttagacgaggctttgtttgactccgtgtagcgacaacaagagaaacaaaactacctatttgtaacggacgtgctgccattgccctccgcattgagcgcctacctattgaaatctttacgtcgggacaatgggagagcggctaaaattaccctcttgggtcctgggcgggcaagattcctgagcccctacccccgcccccatctcatcctcctctaacccgggccttgctgggctcccccttccccagtcccggccgccttctcccagtgtgcgctgcctgcacctgtgcctggagagcatcgaccccgcctcccaggccttgagcccctttgcggcgcagccccagccttgcgcggcctgggctttgcggccaccacaatggaaatctacggggaaaatgccagggctggttctgctggagtcctgggaactctgcgtgggagggagtttgtgactgcggcccaaaagccacctccatacagtgccgtgggatgccaggaagttgaaatcaccctcccccatcgcctgcacttttgagcgcccttccgtctgtgtctttccccagcccccatttgaaagccgcacgaccgaaacccttcttacggggaggcatgggatgggaatggggagtgggggcagacagtagaagcatcccctttgctacggttgaatgaagacagtctagtgggagatgtggctggggctaagaggaagagctgcagtttcctgggccaaagagctgagttggacagggagatggcagcttaccaaggcctgctggttctcagctctagagtctgccttatggtccgagcaggatttatttttaagaacagagcaagttacgtggaagcaaggaaggttttgaggacagaggtttgggtctcctaacttctagtcgggactgtgagaagggcgtgagagagtgttggcacctgtaaggtaagagaggagagcggaagagcgcagtacgggagcggcaccagaggggctggagttgggggggagtgctgtggatgagcgggagaacaatgacacaccaactcctgcactggctgtttccagaaatacgagttggacagccgccctgagccacccactgtgccctgccccacccccgcaccttagctgcttcccgcgtcccatcctcatttaagtaccctgcaccaaaaagtaaatcaatattaagtttaaagaaaaaaaaacccacgtagtcttagtgctgtttacccacttccttcgaaaaggcgtgtggtgtgacctgttgctgcgagaggggatacaaaggtttctcagtggctggcaggctggctctgggagcctcctccccctcctcgcctgccccctcctcccccggcctcccccgcgcggccggcggcgcgggaggccccgccccctttcatgcaaaacccggcagcgaggctgggctcgagtggaggagccgccgcgcgctgattggtcgctagaaacccatttattccctgacagcccccgtcacatg |
| SOX2 promoter-binding site1-mut |
| agaactaaaacaagccataacttgagagaaaaaggagaaccttcggggggcaggaaggttgattggaaataacttaaggaaagtctgcagaattcttttttttacaacttttctgagtttccagtgggtatatttagtgtgagtttgacagtaacaggctagggagggcagagattggagaaattgggggtcgggggagtgattatgggaagaaggttagtaaggaacaaaacaatgcaccgttttgtaaagataataaatggaacgtggctggtagatactattcagtacattttcttagggtgagtaagggtagaccaggggaggagggggcggagagagtgttacagaagaaagaaaataagtaaccctgatggtttaagccctttataaaaaagaaatggcatcaggtttttttttctttattcccccccaccccaccctttgtagtcaagtgcattttagccacaaagatcccaacaagagagtggaaggaaacttagacgaggctttgtttgactccgtgtagcgacaacaagagaaacaaaactacctatttgtaacggacgtgctgccattgccctccgcattgagcgcctacctattgaaatctttacgtcgggacaatgggagagcggctaaaattaccctcttgggtcctgggcgggcaagattcctgagcccctacccccgcccccatctcatcctcctctaacccgggccttgctgggctcccccttccccagtcccggccgccttctcccagtgtgcgctgcctgcacctgtgcctggagagcatcgaccccgcctcccaggccttgagcccctttgcggcgcagccccagccttgcgcggcctgggctttgcggccaccacaatggaaatctacggggaaaatgccagggctggttctgctggagtcctgggaactctgcgtgggagggagtttgtgactgcggcccaaaagccacctccatacagtgccgtgggatgccaggaagttgaaatcaccctcccccatcgcctgcacttttgagcgcccttccgtctgtgtctttccccagcccccatttgaaagccgcacgaccgaaacccttcttacggggaggcatgggatgggaatggggagtgggggcagacagtagaagcatcccctttgctacggttgaatgaagacagtctagtgggagatgtggctggggctaagaggaagagctgcagtttcctgggccaaagagctgagttggacagggagatggcagcttaccaaggcctgctggttctcagctctagagtctgccttatggtccgagcaggatttatttttaagaacagagcaagttacgtggaagcaaggaaggttttgaggacagaggtttgggtctcctaacttctagtcgggactgtgagaagggcgtgagagagtgttggcacctgtaaggtaagagaggagagcggaagagcgcagtacgggagcggcaccagaggggctggagttgggggggagtgctgtggatgagcgggattttttttttttttcaactcctgcactggctgtttccagaaatacgagttggacagccgccctgagccacccactgtgccctgccccacccccgcaccttagctgcttcccgcgtcccatcctcatttaagtaccctgcaccaaaaagtaaatcaatattaagtttaaagaaaaaaaaacccacgtagtcttagtgctgtttacccacttccttcgaaaaggcgtgtggtgtgacctgttgctgcgagaggggatacaaaggtttctcagtggctggcaggctggctctgggagcctcctccccctcctcgcctgccccctcctcccccggcctcccccgcgcggccggcggcgcgggaggccccgccccctttcatgcaaaacccggcagcgaggctgggctcgagtggaggagccgccgcgcgctgattggtcgctagaaacccatttattccctgacagcccccgtcacatg |
| SOX2 promoter-binding site2-mut |
| agaactaaaacaagccataacttgagagaaaaaggagaaccttcggggggcaggaaggttgattggaaataacttaaggaaagtctgcagaattcttttttttacaacttttctgagtttccagtgggtatatttagtgtgagtttgacagtaacaggctagggagggcagagattggagaaattgggggtcgggggagtgattatgggaagaaggttagtaaggaacaaaacaatgcaccgttttgtaaagataataaatggaacgtggctggtagatactattcagtacattttcttagggtgagtaagggtagaccaggggaggagggggcggagagagtgttacagaagaaagaaaataagtaaccctgatggtttaagccctttataaaaaagaattttttttttttttttttttctttattcccccccaccccaccctttgtagtcaagtgcattttagccacaaagatcccaacaagagagtggaaggaaacttagacgaggctttgtttgactccgtgtagcgacaacaagagaaacaaaactacctatttgtaacggacgtgctgccattgccctccgcattgagcgcctacctattgaaatctttacgtcgggacaatgggagagcggctaaaattaccctcttgggtcctgggcgggcaagattcctgagcccctacccccgcccccatctcatcctcctctaacccgggccttgctgggctcccccttccccagtcccggccgccttctcccagtgtgcgctgcctgcacctgtgcctggagagcatcgaccccgcctcccaggccttgagcccctttgcggcgcagccccagccttgcgcggcctgggctttgcggccaccacaatggaaatctacggggaaaatgccagggctggttctgctggagtcctgggaactctgcgtgggagggagtttgtgactgcggcccaaaagccacctccatacagtgccgtgggatgccaggaagttgaaatcaccctcccccatcgcctgcacttttgagcgcccttccgtctgtgtctttccccagcccccatttgaaagccgcacgaccgaaacccttcttacggggaggcatgggatgggaatggggagtgggggcagacagtagaagcatcccctttgctacggttgaatgaagacagtctagtgggagatgtggctggggctaagaggaagagctgcagtttcctgggccaaagagctgagttggacagggagatggcagcttaccaaggcctgctggttctcagctctagagtctgccttatggtccgagcaggatttatttttaagaacagagcaagttacgtggaagcaaggaaggttttgaggacagaggtttgggtctcctaacttctagtcgggactgtgagaagggcgtgagagagtgttggcacctgtaaggtaagagaggagagcggaagagcgcagtacgggagcggcaccagaggggctggagttgggggggagtgctgtggatgagcgggagaacaatgacacaccaactcctgcactggctgtttccagaaatacgagttggacagccgccctgagccacccactgtgccctgccccacccccgcaccttagctgcttcccgcgtcccatcctcatttaagtaccctgcaccaaaaagtaaatcaatattaagtttaaagaaaaaaaaacccacgtagtcttagtgctgtttacccacttccttcgaaaaggcgtgtggtgtgacctgttgctgcgagaggggatacaaaggtttctcagtggctggcaggctggctctgggagcctcctccccctcctcgcctgccccctcctcccccggcctcccccgcgcggccggcggcgcgggaggccccgccccctttcatgcaaaacccggcagcgaggctgggctcgagtggaggagccgccgcgcgctgattggtcgctagaaacccatttattccctgacagcccccgtcacatg |
| SOX2 promoter-binding site3-mut |
| agaactaaaacaagccataacttgagagaaaaaggagaaccttcggggggcaggaaggttgattggaaataacttaaggaaagtctgcagaattcttttttttacaacttttctgagtttccagtgggtatatttagtgtgagtttgacagtaacaggctagggagggcagagattggagaaattgggggtcgggggagtgattatgggaagaaggttagtaaggaacaaaacaatgcaccgttttgtaaagataataaatggaacgtggctggtagatactattcagtacattttcttttttttttttttggtagaccaggggaggagggggcggagagagtgttacagaagaaagaaaataagtaaccctgatggtttaagccctttataaaaaagaaatggcatcaggtttttttttctttattcccccccaccccaccctttgtagtcaagtgcattttagccacaaagatcccaacaagagagtggaaggaaacttagacgaggctttgtttgactccgtgtagcgacaacaagagaaacaaaactacctatttgtaacggacgtgctgccattgccctccgcattgagcgcctacctattgaaatctttacgtcgggacaatgggagagcggctaaaattaccctcttgggtcctgggcgggcaagattcctgagcccctacccccgcccccatctcatcctcctctaacccgggccttgctgggctcccccttccccagtcccggccgccttctcccagtgtgcgctgcctgcacctgtgcctggagagcatcgaccccgcctcccaggccttgagcccctttgcggcgcagccccagccttgcgcggcctgggctttgcggccaccacaatggaaatctacggggaaaatgccagggctggttctgctggagtcctgggaactctgcgtgggagggagtttgtgactgcggcccaaaagccacctccatacagtgccgtgggatgccaggaagttgaaatcaccctcccccatcgcctgcacttttgagcgcccttccgtctgtgtctttccccagcccccatttgaaagccgcacgaccgaaacccttcttacggggaggcatgggatgggaatggggagtgggggcagacagtagaagcatcccctttgctacggttgaatgaagacagtctagtgggagatgtggctggggctaagaggaagagctgcagtttcctgggccaaagagctgagttggacagggagatggcagcttaccaaggcctgctggttctcagctctagagtctgccttatggtccgagcaggatttatttttaagaacagagcaagttacgtggaagcaaggaaggttttgaggacagaggtttgggtctcctaacttctagtcgggactgtgagaagggcgtgagagagtgttggcacctgtaaggtaagagaggagagcggaagagcgcagtacgggagcggcaccagaggggctggagttgggggggagtgctgtggatgagcgggagaacaatgacacaccaactcctgcactggctgtttccagaaatacgagttggacagccgccctgagccacccactgtgccctgccccacccccgcaccttagctgcttcccgcgtcccatcctcatttaagtaccctgcaccaaaaagtaaatcaatattaagtttaaagaaaaaaaaacccacgtagtcttagtgctgtttacccacttccttcgaaaaggcgtgtggtgtgacctgttgctgcgagaggggatacaaaggtttctcagtggctggcaggctggctctgggagcctcctccccctcctcgcctgccccctcctcccccggcctcccccgcgcggccggcggcgcgggaggccccgccccctttcatgcaaaacccggcagcgaggctgggctcgagtggaggagccgccgcgcgctgattggtcgctagaaacccatttattccctgacagcccccgtcacatg |
| SOX2 promoter-binding site1+2-mut |
| agaactaaaacaagccataacttgagagaaaaaggagaaccttcggggggcaggaaggttgattggaaataacttaaggaaagtctgcagaattcttttttttacaacttttctgagtttccagtgggtatatttagtgtgagtttgacagtaacaggctagggagggcagagattggagaaattgggggtcgggggagtgattatgggaagaaggttagtaaggaacaaaacaatgcaccgttttgtaaagataataaatggaacgtggctggtagatactattcagtacattttcttagggtgagtaagggtagaccaggggaggagggggcggagagagtgttacagaagaaagaaaataagtaaccctgatggtttaagccctttataaaaaagaattttttttttttttttttttctttattcccccccaccccaccctttgtagtcaagtgcattttagccacaaagatcccaacaagagagtggaaggaaacttagacgaggctttgtttgactccgtgtagcgacaacaagagaaacaaaactacctatttgtaacggacgtgctgccattgccctccgcattgagcgcctacctattgaaatctttacgtcgggacaatgggagagcggctaaaattaccctcttgggtcctgggcgggcaagattcctgagcccctacccccgcccccatctcatcctcctctaacccgggccttgctgggctcccccttccccagtcccggccgccttctcccagtgtgcgctgcctgcacctgtgcctggagagcatcgaccccgcctcccaggccttgagcccctttgcggcgcagccccagccttgcgcggcctgggctttgcggccaccacaatggaaatctacggggaaaatgccagggctggttctgctggagtcctgggaactctgcgtgggagggagtttgtgactgcggcccaaaagccacctccatacagtgccgtgggatgccaggaagttgaaatcaccctcccccatcgcctgcacttttgagcgcccttccgtctgtgtctttccccagcccccatttgaaagccgcacgaccgaaacccttcttacggggaggcatgggatgggaatggggagtgggggcagacagtagaagcatcccctttgctacggttgaatgaagacagtctagtgggagatgtggctggggctaagaggaagagctgcagtttcctgggccaaagagctgagttggacagggagatggcagcttaccaaggcctgctggttctcagctctagagtctgccttatggtccgagcaggatttatttttaagaacagagcaagttacgtggaagcaaggaaggttttgaggacagaggtttgggtctcctaacttctagtcgggactgtgagaagggcgtgagagagtgttggcacctgtaaggtaagagaggagagcggaagagcgcagtacgggagcggcaccagaggggctggagttgggggggagtgctgtggatgagcgggattttttttttttttcaactcctgcactggctgtttccagaaatacgagttggacagccgccctgagccacccactgtgccctgccccacccccgcaccttagctgcttcccgcgtcccatcctcatttaagtaccctgcaccaaaaagtaaatcaatattaagtttaaagaaaaaaaaacccacgtagtcttagtgctgtttacccacttccttcgaaaaggcgtgtggtgtgacctgttgctgcgagaggggatacaaaggtttctcagtggctggcaggctggctctgggagcctcctccccctcctcgcctgccccctcctcccccggcctcccccgcgcggccggcggcgcgggaggccccgccccctttcatgcaaaacccggcagcgaggctgggctcgagtggaggagccgccgcgcgctgattggtcgctagaaacccatttattccctgacagcccccgtcacatg |
| SOX2 promoter-binding site1+3-mut |
| agaactaaaacaagccataacttgagagaaaaaggagaaccttcggggggcaggaaggttgattggaaataacttaaggaaagtctgcagaattcttttttttacaacttttctgagtttccagtgggtatatttagtgtgagtttgacagtaacaggctagggagggcagagattggagaaattgggggtcgggggagtgattatgggaagaaggttagtaaggaacaaaacaatgcaccgttttgtaaagataataaatggaacgtggctggtagatactattcagtacattttcttttttttttttttggtagaccaggggaggagggggcggagagagtgttacagaagaaagaaaataagtaaccctgatggtttaagccctttataaaaaagaaatggcatcaggtttttttttctttattcccccccaccccaccctttgtagtcaagtgcattttagccacaaagatcccaacaagagagtggaaggaaacttagacgaggctttgtttgactccgtgtagcgacaacaagagaaacaaaactacctatttgtaacggacgtgctgccattgccctccgcattgagcgcctacctattgaaatctttacgtcgggacaatgggagagcggctaaaattaccctcttgggtcctgggcgggcaagattcctgagcccctacccccgcccccatctcatcctcctctaacccgggccttgctgggctcccccttccccagtcccggccgccttctcccagtgtgcgctgcctgcacctgtgcctggagagcatcgaccccgcctcccaggccttgagcccctttgcggcgcagccccagccttgcgcggcctgggctttgcggccaccacaatggaaatctacggggaaaatgccagggctggttctgctggagtcctgggaactctgcgtgggagggagtttgtgactgcggcccaaaagccacctccatacagtgccgtgggatgccaggaagttgaaatcaccctcccccatcgcctgcacttttgagcgcccttccgtctgtgtctttccccagcccccatttgaaagccgcacgaccgaaacccttcttacggggaggcatgggatgggaatggggagtgggggcagacagtagaagcatcccctttgctacggttgaatgaagacagtctagtgggagatgtggctggggctaagaggaagagctgcagtttcctgggccaaagagctgagttggacagggagatggcagcttaccaaggcctgctggttctcagctctagagtctgccttatggtccgagcaggatttatttttaagaacagagcaagttacgtggaagcaaggaaggttttgaggacagaggtttgggtctcctaacttctagtcgggactgtgagaagggcgtgagagagtgttggcacctgtaaggtaagagaggagagcggaagagcgcagtacgggagcggcaccagaggggctggagttgggggggagtgctgtggatgagcgggattttttttttttttcaactcctgcactggctgtttccagaaatacgagttggacagccgccctgagccacccactgtgccctgccccacccccgcaccttagctgcttcccgcgtcccatcctcatttaagtaccctgcaccaaaaagtaaatcaatattaagtttaaagaaaaaaaaacccacgtagtcttagtgctgtttacccacttccttcgaaaaggcgtgtggtgtgacctgttgctgcgagaggggatacaaaggtttctcagtggctggcaggctggctctgggagcctcctccccctcctcgcctgccccctcctcccccggcctcccccgcgcggccggcggcgcgggaggccccgccccctttcatgcaaaacccggcagcgaggctgggctcgagtggaggagccgccgcgcgctgattggtcgctagaaacccatttattccctgacagcccccgtcacatg |
| SOX2 promoter-binding site2+3-mut |
| agaactaaaacaagccataacttgagagaaaaaggagaaccttcggggggcaggaaggttgattggaaataacttaaggaaagtctgcagaattcttttttttacaacttttctgagtttccagtgggtatatttagtgtgagtttgacagtaacaggctagggagggcagagattggagaaattgggggtcgggggagtgattatgggaagaaggttagtaaggaacaaaacaatgcaccgttttgtaaagataataaatggaacgtggctggtagatactattcagtacattttcttttttttttttttggtagaccaggggaggagggggcggagagagtgttacagaagaaagaaaataagtaaccctgatggtttaagccctttataaaaaagaattttttttttttttttttttctttattcccccccaccccaccctttgtagtcaagtgcattttagccacaaagatcccaacaagagagtggaaggaaacttagacgaggctttgtttgactccgtgtagcgacaacaagagaaacaaaactacctatttgtaacggacgtgctgccattgccctccgcattgagcgcctacctattgaaatctttacgtcgggacaatgggagagcggctaaaattaccctcttgggtcctgggcgggcaagattcctgagcccctacccccgcccccatctcatcctcctctaacccgggccttgctgggctcccccttccccagtcccggccgccttctcccagtgtgcgctgcctgcacctgtgcctggagagcatcgaccccgcctcccaggccttgagcccctttgcggcgcagccccagccttgcgcggcctgggctttgcggccaccacaatggaaatctacggggaaaatgccagggctggttctgctggagtcctgggaactctgcgtgggagggagtttgtgactgcggcccaaaagccacctccatacagtgccgtgggatgccaggaagttgaaatcaccctcccccatcgcctgcacttttgagcgcccttccgtctgtgtctttccccagcccccatttgaaagccgcacgaccgaaacccttcttacggggaggcatgggatgggaatggggagtgggggcagacagtagaagcatcccctttgctacggttgaatgaagacagtctagtgggagatgtggctggggctaagaggaagagctgcagtttcctgggccaaagagctgagttggacagggagatggcagcttaccaaggcctgctggttctcagctctagagtctgccttatggtccgagcaggatttatttttaagaacagagcaagttacgtggaagcaaggaaggttttgaggacagaggtttgggtctcctaacttctagtcgggactgtgagaagggcgtgagagagtgttggcacctgtaaggtaagagaggagagcggaagagcgcagtacgggagcggcaccagaggggctggagttgggggggagtgctgtggatgagcgggagaacaatgacacaccaactcctgcactggctgtttccagaaatacgagttggacagccgccctgagccacccactgtgccctgccccacccccgcaccttagctgcttcccgcgtcccatcctcatttaagtaccctgcaccaaaaagtaaatcaatattaagtttaaagaaaaaaaaacccacgtagtcttagtgctgtttacccacttccttcgaaaaggcgtgtggtgtgacctgttgctgcgagaggggatacaaaggtttctcagtggctggcaggctggctctgggagcctcctccccctcctcgcctgccccctcctcccccggcctcccccgcgcggccggcggcgcgggaggccccgccccctttcatgcaaaacccggcagcgaggctgggctcgagtggaggagccgccgcgcgctgattggtcgctagaaacccatttattccctgacagcccccgtcacatg |
| SOX2 promoter-binding site1+2+3-mut |
| agaactaaaacaagccataacttgagagaaaaaggagaaccttcggggggcaggaaggttgattggaaataacttaaggaaagtctgcagaattcttttttttacaacttttctgagtttccagtgggtatatttagtgtgagtttgacagtaacaggctagggagggcagagattggagaaattgggggtcgggggagtgattatgggaagaaggttagtaaggaacaaaacaatgcaccgttttgtaaagataataaatggaacgtggctggtagatactattcagtacattttcttttttttttttttggtagaccaggggaggagggggcggagagagtgttacagaagaaagaaaataagtaaccctgatggtttaagccctttataaaaaagaattttttttttttttttttttctttattcccccccaccccaccctttgtagtcaagtgcattttagccacaaagatcccaacaagagagtggaaggaaacttagacgaggctttgtttgactccgtgtagcgacaacaagagaaacaaaactacctatttgtaacggacgtgctgccattgccctccgcattgagcgcctacctattgaaatctttacgtcgggacaatgggagagcggctaaaattaccctcttgggtcctgggcgggcaagattcctgagcccctacccccgcccccatctcatcctcctctaacccgggccttgctgggctcccccttccccagtcccggccgccttctcccagtgtgcgctgcctgcacctgtgcctggagagcatcgaccccgcctcccaggccttgagcccctttgcggcgcagccccagccttgcgcggcctgggctttgcggccaccacaatggaaatctacggggaaaatgccagggctggttctgctggagtcctgggaactctgcgtgggagggagtttgtgactgcggcccaaaagccacctccatacagtgccgtgggatgccaggaagttgaaatcaccctcccccatcgcctgcacttttgagcgcccttccgtctgtgtctttccccagcccccatttgaaagccgcacgaccgaaacccttcttacggggaggcatgggatgggaatggggagtgggggcagacagtagaagcatcccctttgctacggttgaatgaagacagtctagtgggagatgtggctggggctaagaggaagagctgcagtttcctgggccaaagagctgagttggacagggagatggcagcttaccaaggcctgctggttctcagctctagagtctgccttatggtccgagcaggatttatttttaagaacagagcaagttacgtggaagcaaggaaggttttgaggacagaggtttgggtctcctaacttctagtcgggactgtgagaagggcgtgagagagtgttggcacctgtaaggtaagagaggagagcggaagagcgcagtacgggagcggcaccagaggggctggagttgggggggagtgctgtggatgagcgggattttttttttttttcaactcctgcactggctgtttccagaaatacgagttggacagccgccctgagccacccactgtgccctgccccacccccgcaccttagctgcttcccgcgtcccatcctcatttaagtaccctgcaccaaaaagtaaatcaatattaagtttaaagaaaaaaaaacccacgtagtcttagtgctgtttacccacttccttcgaaaaggcgtgtggtgtgacctgttgctgcgagaggggatacaaaggtttctcagtggctggcaggctggctctgggagcctcctccccctcctcgcctgccccctcctcccccggcctcccccgcgcggccggcggcgcgggaggccccgccccctttcatgcaaaacccggcagcgaggctgggctcgagtggaggagccgccgcgcgctgattggtcgctagaaacccatttattccctgacagcccccgtcacatg |
| OCT4 promoter-binding site1-wt |
| gtcgacagaggtcagcgtgcccagtccagacctggccttctggccttcgaagctgtggggagccctggcccagagccccctctggagcccccagacttaccccaggccctccactgagatcaagttttgggagcagacagacaaacatcatccctcacagacaggcattccgttggctattctcttgcaaacagaatcaagcactagaccagcagcatgagcctcaggatactcaggccaggcccagaaaaacagaccctgaaggggagcttagggcagccttcctgcacgcctccacaaatcactctccacctcctctgcgtctttctgccagccagccccactaaacaaagcacatccctcaatctgccaggctcggggagggacgcacgatgaagctggacgcctgagtcccccagaggaaggaggaactagatacctaggtccctgtggggggcccttggtgcccgtctgaggctcagtctttgaggggattgcagaggggggttgctggagctccttttagcgtctctgaaggggattctgtgtgaggggattgggactggggggttggggagcaggaagcagtccccaggggagccatccaggcccattcaagggttgagcacttgtttagggttagagctgccccctctggggaccgggattgtccagccaaggccattgtcctgcccccttcccccagtccctcccaggcttctttgaacctgaagtcagatattttttctccacaccccccaccccctggttttccccacccagggcctagggctggaggcctgggccagggaggtgggggagggagaacggggcctaccgtggtattagatgtctgagttttggttgagaggggagcaaggaacctgatgtgcaggttccatagtggagggggcccaaagcgggtgtcttatcactctgtttcagcaaaggttgggaaactgaggcccagtcagtccaaagtctggtcccttgaaggggaagtagggaccaaccccttagtctgttagatgaggagagtctggagtctgattctggaagacggaggggtggggggatgggggggtggggggatatagcacggaggccttgtctggcagtctactcttgaagatggggtgaaatttggcaggctgggcagatggtgccaggcacccaggctgcggggtggctggatttggccagtatcgggatgggaatgcctaggattctggatggatcgggggaaggcataagggagcagctggccattgtgcttatggctgttgatgcattgagggatagcgccacacacacattcaataaatttgaggagctgagagggtgactggcccctgaaggcacagtgccagaggtctgtggagagggggtcaagcacctgggttcctgaagaacatggaggtgtgggagtgattccagacagctgggatgtgcagagcctgagagagtgccagggagcgggttgggagttgaaagttgggtgtggtggctcacgcctttaatcatgacactgggcggcagaggcgggaggatttcttgaggacaggaattcaagaccagcctgggtaacatagcaaggccccatctctactaaaaataaaaaaactaacagggcacagtggtccaagcctgtagtcccagccacttaggaggctggagcagaaggattgctttggcccagtagatcgaggctacattgagccatcattgtactccactgcactccagtctgggcaacaaagtgagaccctgtcttaaaaaataaaaataaaaaaagtttctgtgggggacctgcactgaggtcctggaggggcgccagttgtgtctcccggttttccccttccacagacaccattgccaccaccattaggcaaacatccttcgcctcagtttctccccccacctccctctcctccacccatccagggggcggggccagaggtcaaggctagtgggtgggactggggagggagagaggggtt |
| OCT4 promoter-binding site1-mut |
| gtcgacagaggtcagcgtgcccagtccagacctggccttctggccttcgaagctgtggggagccctggcccagagccccctctggagcccccagacttaccccaggccctccactgagatcaagttttgggagcagacagacaaacatcatccctcacagacaggcattccgttggctattctcttgcaaacagaatcaagcactagaccagcagcatgagcctcaggatactcaggccaggcccagaaaaacagaccctgaaggggagcttagggcagccttcctgcacgcctccacaaatcactctccacctcctctgcgtctttctgccagccagccccactaaacaaagcacatccctcaatctgccaggctcggggagggacgcacgatgaagctggacgcctgagtcccccagaggaaggaggaactagatacctaggtccctgtggggggcccttggtgcccgtctgaggctcagtctttgaggggattgcagaggggggttgctggagctccttttagcgtctctgaaggggattctgtgtgaggggattgggactggggggttggggagcaggaagcagtccccaggggagccatccaggcccattcaagggttgagcacttgtttagggttagagctgccccctctggggaccgggattgtccagccaaggccattgtcctgcccccttcccccagtccctcccaggcttctttgaacctgaagtcagatattttttctccacaccccccaccccctggttttccccacccagggcctagggctggaggcctgggccagggaggtgggggagggagaacggggcctaccgtggtattagatgtctgagttttggttgagaggggagcaaggaacctgatgtgcaggttccatagtggagggggcccaaagcgggtgtcttatcactctgtttcagcaaaggttgggaaactgaggcccagtcagtccaaagtctggtcccttgaaggggaagtagggaccaaccccttagtctgttagatgaggagagtctggagtctgattctggaagacggaggggtggggggatgggggggtggggggatatagcacggaggccttgtctggcagtctactcttgaagatggggtgaaatttggcaggctgggcagatggtgccaggcacccaggctgcggggtggctggatttggccagtatcgggatgggaatgcctaggattctggatggatcgggggaaggcataagggagcagctggccattgtgcttatggctgttgatgcattgagggatagcgccacacacacattcaataaatttgaggagctgagagggtgactggcccctgaaggcacagtgccagaggtctgtggagagggggtcaagcacctgggttcctgaagaacatggaggtgtgggagtgattccagacagctgggatgtgcagagcctgagagagtgccagggagcgggttgggagttgaaagttggttttttttttttttgcctttaatcatgacactgggcggcagaggcgggaggatttcttgaggacaggaattcaagaccagcctgggtaacatagcaaggccccatctctactaaaaataaaaaaactaacagggcacagtggtccaagcctgtagtcccagccacttaggaggctggagcagaaggattgctttggcccagtagatcgaggctacattgagccatcattgtactccactgcactccagtctgggcaacaaagtgagaccctgtcttaaaaaataaaaataaaaaaagtttctgtgggggacctgcactgaggtcctggaggggcgccagttgtgtctcccggttttccccttccacagacaccattgccaccaccattaggcaaacatccttcgcctcagtttctccccccacctccctctcctccacccatccagggggcggggccagaggtcaaggctagtgggtgggactggggagggagagaggggtt |
| c-Jun promoter-full length sequence |
| tacaacagaaaatgattcagggcaacagacagaggagaatgttctctccttgaggaagcaactggatcttgtcatcactgtatacctacctaccccaccccctccccagctcagtgcctggctcacagtaggctttcagttaccctctgcagatcagtgaaagctaggtgagtgcccggagtgaagaaaagttggcaggtttcccactgataccagctgctgttggtttctgaacactcaaagccgcaaataccttagggctgggggcaatgaacccaaggctgaattccaagttcagaagcagcgaagtctgaatttagaacctaggaacttaaactgctgcaggtccaacttcaagccccagttttagacagaggcttgggaaagatctgacttctaacccggttccccctcccctcctcccctcgatgcttctcacaggaaagtacacctggtcctgccaaatcgcactcttatatcctggcatcctatccaggctctgcgaggatggaaactgcgaggcaggggagggaagcgggctgtttggccaccacctccctagtgctgcaggcgaccctgtcacactaactcctggcagcccagtgaggtggacggcaccggccccacctgcagatgagggaaatgaagctcggaggagttccgtgatttgcttgcttcacactgtggtagcctggccacgaaagaaccaggattcccgacttcgggattctttccaccacacactttcgtccctaaggggtggggggcggggggagaataaaataaccgcggaaaaggaaccacttacatgtgtctagcgcttcctagaggctacccaggatatgcgcccaccacccggccgggagtgcagagatttgaagtccaggttctaccccgggctccgagtactactgcgtgactttatgcgagtgtccgccgccttctgggcttgttttcccggaagcaactcggcgcggatggagtgtgtgtgtgcgcgcgcgcgcgcgttatgttgtgcgtgttgtgttaagcgtgtgcgtgttgtccgggggcgggagggggagtagactaacaccggggttccccgagtttcggatcgcctacacgcttgttcccatctggaccctgttacccaccaattgcgcccactataaaaactgcccctccgaggcaaagctgtgaacccccgcgccctttcccccacggtcccggaggatgaagtggggtgcaacggagactcagctgagcgtccagtttcgggcaatacaaatctctcggcttctacgagcagccagacgaccccgcggaccgtcgctcctgaacttgaccgagatgcaaacttcggagtgttctcaacgtggggggccgactctcgggagaccgcccctaaacttaagtccccttaggctcgcccccacctgggacttcacagagccaccttaagggcggtattcccgcccccccggaagtgcgggggggtggcagcgtacttggattctcagcctccagccccgcgcggtggcggccgccggtggatgacttcgggccccacaagtggggaaacaacaaccacccctcgcccgcacccctggcccaaaacaactggccaggttccctggcctcccgggtccctgcatcccccgcatccccgtccgcagccgtgaacttgagcccccctccatcagaggttgcgagcgtccgcccgctcgcggcagccaccgtcactagacagtcaaaccccaagacgtcagcccacaatgcaccgggcgggccgggaaaaacggcccggggaggggaccggggaagagagggccgagaggcgtgcggcaggggggagggtaggagaaagaagggcccgactgtaggagggcagcggagcattacctcatcccgtgagcctccgcgggcccagagaagaatcttctagggtggagtctccatggtgacgggcgggcccgcccccctgagagcgacgcgagccaatgggaaggccttggggt |
| c-Jun promoter-TEAD binding site1-mut |
| tacaacagaaaatgattcagggcaacagacagaggagaatgttctctccttgaggaagcaactggatcttgtcatcactgtatacctacctaccccaccccctccccagctcagtgcctggctcacagtaggctttcagttaccctctgcagatcagtgaaagctaggtgagtgcccggagtgaagaaaagttggcaggtttcccactgataccagctgctgttggtttctgaacactcaaagccgcaaataccttagggctgggggcaatgaacccaaggcaaaaaaaaaagttcagaagcagcgaagtctgaatttagaacctaggaacttaaactgctgcaggtccaacttcaagccccagttttagacagaggcttgggaaagatctgacttctaacccggttccccctcccctcctcccctcgatgcttctcacaggaaagtacacctggtcctgccaaatcgcactcttatatcctggcatcctatccaggctctgcgaggatggaaactgcgaggcaggggagggaagcgggctgtttggccaccacctccctagtgctgcaggcgaccctgtcacactaactcctggcagcccagtgaggtggacggcaccggccccacctgcagatgagggaaatgaagctcggaggagttccgtgatttgcttgcttcacactgtggtagcctggccacgaaagaaccaggattcccgacttcgggattctttccaccacacactttcgtccctaaggggtggggggcggggggagaataaaataaccgcggaaaaggaaccacttacatgtgtctagcgcttcctagaggctacccaggatatgcgcccaccacccggccgggagtgcagagatttgaagtccaggttctaccccgggctccgagtactactgcgtgactttatgcgagtgtccgccgccttctgggcttgttttcccggaagcaactcggcgcggatggagtgtgtgtgtgcgcgcgcgcgcgcgttatgttgtgcgtgttgtgttaagcgtgtgcgtgttgtccgggggcgggagggggagtagactaacaccggggttccccgagtttcggatcgcctacacgcttgttcccatctggaccctgttacccaccaattgcgcccactataaaaactgcccctccgaggcaaagctgtgaacccccgcgccctttcccccacggtcccggaggatgaagtggggtgcaacggagactcagctgagcgtccagtttcgggcaatacaaatctctcggcttctacgagcagccagacgaccccgcggaccgtcgctcctgaacttgaccgagatgcaaacttcggagtgttctcaacgtggggggccgactctcgggagaccgcccctaaacttaagtccccttaggctcgcccccacctgggacttcacagagccaccttaagggcggtattcccgcccccccggaagtgcgggggggtggcagcgtacttggattctcagcctccagccccgcgcggtggcggccgccggtggatgacttcgggccccacaagtggggaaacaacaaccacccctcgcccgcacccctggcccaaaacaactggccaggttccctggcctcccgggtccctgcatcccccgcatccccgtccgcagccgtgaacttgagcccccctccatcagaggttgcgagcgtccgcccgctcgcggcagccaccgtcactagacagtcaaaccccaagacgtcagcccacaatgcaccgggcgggccgggaaaaacggcccggggaggggaccggggaagagagggccgagaggcgtgcggcaggggggagggtaggagaaagaagggcccgactgtaggagggcagcggagcattacctcatcccgtgagcctccgcgggcccagagaagaatcttctagggtggagtctccatggtgacgggcgggcccgcccccctgagagcgacgcgagccaatgggaaggccttggggt |
| c-Jun promoter-TEAD binding site2-mut |
| tacaacagaaaatgattcagggcaacagacagaggagaatgttctctccttgaggaagcaactggatcttgtcatcactgtatacctacctaccccaccccctccccagctcagtgcctggctcacagtaggctttcagttaccctctgcagatcagtgaaagctaggtgagtgcccggagtgaagaaaagttggcaggtttcccactgataccagctgctgttggtttctgaacactcaaagccgaaaaaaaaaaagggctgggggcaatgaacccaaggctgaattccaagttcagaagcagcgaagtctgaatttagaacctaggaacttaaactgctgcaggtccaacttcaagccccagttttagacagaggcttgggaaagatctgacttctaacccggttccccctcccctcctcccctcgatgcttctcacaggaaagtacacctggtcctgccaaatcgcactcttatatcctggcatcctatccaggctctgcgaggatggaaactgcgaggcaggggagggaagcgggctgtttggccaccacctccctagtgctgcaggcgaccctgtcacactaactcctggcagcccagtgaggtggacggcaccggccccacctgcagatgagggaaatgaagctcggaggagttccgtgatttgcttgcttcacactgtggtagcctggccacgaaagaaccaggattcccgacttcgggattctttccaccacacactttcgtccctaaggggtggggggcggggggagaataaaataaccgcggaaaaggaaccacttacatgtgtctagcgcttcctagaggctacccaggatatgcgcccaccacccggccgggagtgcagagatttgaagtccaggttctaccccgggctccgagtactactgcgtgactttatgcgagtgtccgccgccttctgggcttgttttcccggaagcaactcggcgcggatggagtgtgtgtgtgcgcgcgcgcgcgcgttatgttgtgcgtgttgtgttaagcgtgtgcgtgttgtccgggggcgggagggggagtagactaacaccggggttccccgagtttcggatcgcctacacgcttgttcccatctggaccctgttacccaccaattgcgcccactataaaaactgcccctccgaggcaaagctgtgaacccccgcgccctttcccccacggtcccggaggatgaagtggggtgcaacggagactcagctgagcgtccagtttcgggcaatacaaatctctcggcttctacgagcagccagacgaccccgcggaccgtcgctcctgaacttgaccgagatgcaaacttcggagtgttctcaacgtggggggccgactctcgggagaccgcccctaaacttaagtccccttaggctcgcccccacctgggacttcacagagccaccttaagggcggtattcccgcccccccggaagtgcgggggggtggcagcgtacttggattctcagcctccagccccgcgcggtggcggccgccggtggatgacttcgggccccacaagtggggaaacaacaaccacccctcgcccgcacccctggcccaaaacaactggccaggttccctggcctcccgggtccctgcatcccccgcatccccgtccgcagccgtgaacttgagcccccctccatcagaggttgcgagcgtccgcccgctcgcggcagccaccgtcactagacagtcaaaccccaagacgtcagcccacaatgcaccgggcgggccgggaaaaacggcccggggaggggaccggggaagagagggccgagaggcgtgcggcaggggggagggtaggagaaagaagggcccgactgtaggagggcagcggagcattacctcatcccgtgagcctccgcgggcccagagaagaatcttctagggtggagtctccatggtgacgggcgggcccgcccccctgagagcgacgcgagccaatgggaaggccttggggt |
